# A New Concept of Enhancing the Anticancer Activity of Manganese Terpyridine Complex by Oxygen-Containing Substituent Modification

**DOI:** 10.3390/ijms24043903

**Published:** 2023-02-15

**Authors:** Jiahe Li, Min Chen, Jinzhang Jiang, Jieyou Huang, Hailan Chen, Lixia Pan, Dmytro S. Nesterov, Zhen Ma, Armando J. L. Pombeiro

**Affiliations:** 1 School of Chemistry and Chemical Engineering, Guangxi University, Nanning 530004, China; 2 National Engineering Research Center for Non-Food Biorefinery, State Key Laboratory of Non-Food Biomass and Enzyme Technology, Guangxi Academy of Sciences, Nanning 530007, China; 3 Centro de Química Estrutural, Institute of Molecular Sciences, Instituto Superior Técnico, Universidade de Lisboa, 1049-001 Lisbon, Portugal; 4 School of Animal Science and Technology, Guangxi University, Nanning 530004, China; 5 Research Institute of Chemistry, Peoples’ Friendship University of Russia (RUDN University), Moscow 117198, Russia

**Keywords:** terpyridine, manganese complex, anticancer activity, DNA interaction, molecular docking

## Abstract

Eleven manganese 4′-substituted-2,2′:6′,2″-terpyridine complexes (**1a**–**1c** and **2a**–**2h**) with three non-oxygen-containing substituents (**L^1a^**–**L^1c^**: phenyl, naphthalen-2-yl and naphthalen-1-yl, **L^1a^**–**L^1c^**) and eight oxygen-containing substituents (**L^2a^**–**L^2h^**: 4-hydroxyl-phenyl, 3-hydroxyl-phenyl, 2-hydroxyl-phenyl, 4-methoxyl-phenyl, 4-carboxyl-phenyl, 4-(methylsulfonyl)phenyl, 4-nitrophenyl and furan-2-yl) were prepared and characterized by IR, elemental analysis or single crystal X-ray diffraction. In vitro data demonstrate that all of these show higher antiproliferative activities than cisplatin against five human carcinoma cell lines: A549, Bel-7402, Eca-109, HeLa and MCF-7. Compound **2d** presents the strongest antiproliferative effect against A549 and HeLa cells, with IC_50_ values being 0.281 μM and 0.356 μM, respectively. The lowest IC_50_ values against Bel-7402 (0.523 μM) Eca-109 (0.514 μM) and MCF-7 (0.356 μM) were obtained for compounds **2h**, **2g** and **2c**, respectively. Compound **2g** with a nitro group showed the best results on the whole, with relevantly low IC_50_ values against all the tested tumor cells. The DNA interactions with these compounds were studied by circular dichroism spectroscopic and molecular modeling methods. Spectrophotometric results revealed that the compounds have strong affinities in binding with DNA as intercalators, and the binding induces DNA conformational transition. Molecular docking studies indicate that the binding is contributed by the π–π stacking and hydrogen bonds. The anticancer activities of the compounds are correlated with their DNA binding ability, and the modification of oxygen-containing substituents significantly enhanced the anticancer activity, which could provide a new rationale for the future design of terpyridine-based metal complexes with antitumor potential.

## 1. Introduction

Cancer is considered the most deadly disease impacting the different countries of the world [1,2,3]. According to WHO reports, as of 2018, cancer was the leading cause of death, with a global estimate of around 9.6 million, and one out of every six cases of death is due to cancer worldwide [4]. Metal-based drugs have been known and widely used as early as in ancient medicine. In modern times, a new era in oncology has been reopened using platinum anticancer agents [5]. Platinum drugs attracted attention in cancer treatment once the antineoplastic activity of cisplatin was discovered in the 1960s [6,7,8,9]. Despite the broad anticancer potential of platinum drugs, their clinical use has regularly been constrained by renal toxicity, low solubility and high cross-resistance [10]. To overcome these adverse reactions, the design and development of new drugs that have high efficacy, good bioavailability and weak cross-resistance are hotspots in the research of anti-tumor metal complexes [11,12,13].

As very popular ligands in coordination chemistry, terpyridine and its derivatives are a kind of multidentate N-donor ligands with a strong coordination ability to transition metals, and the planar aromatic ring structure is conducive to the interaction with DNA [14,15,16,17,18,19,20,21,22,23,24,25]. The metal complexes of terpyridine and its derivatives have rich photophysical and electrical properties. Therefore, they have important research and application prospects in the fields of luminescent materials, photodynamic therapy and chemical sensors [26,27,28,29,30,31,32]. After the success of cisplatin and carboplatin in cancer chemotherapy, other transition metal ions have been of great interest, since they are involved in many biological processes [33,34,35,36,37,38,39,40,41,42,43,44,45]. Among them, manganese is used to synthesize new complexes that exhibit excellent antibacterial and antitumor activity [46,47,48].

Due to their multi-faceted biological activity, a clear-cut target is not always easy to identify for metal or metalloid drugs [49]. Clinically approved platinum drugs are widely accepted to target DNA, and their activities are known to be influenced by DNA damage response pathways or respective repair mechanisms [50,51]. Therefore, DNA is considered the primary target for most anticancer and antiviral therapeutics [52,53,54,55,56]. DNA Topoisomerases (Topo) are ubiquitous nuclear enzymes involved in regulating the topological state of DNA, and in eukaryotic organisms, Topo can be classified into two structurally and functionally different main classes: Topo I and Topo II. Both these enzymes are the cellular target of clinically important anticancer and antibacterial drugs, and their inhibition has been considered an effective strategy for the design of many anticancer agents. At the same time, Topo I or II inhibitors show considerable wide spectrum antitumor activities, an important feature to be included in many chemotherapeutic protocols [57,58,59,60,61,62,63].

Although many terpyridine complexes have shown an interesting antiproliferative activity against tumor cells [21,22,23,24], the factors that govern the activity and the biological target are unknown. In this work, a series of manganese dichloride-substituted terpyridine complexes were synthesized by the reaction of manganese dichloride with the corresponding 4′-substituted-2,2′:6′,2″-terpyridine-bearing phenyl (**L^1a^**), naphthalen-2-yl (**L^1b^**), naphthalen-1-yl (**L^1c^**), 4-hydroxyl-phenyl (**L^2a^**), 3-hydroxyl-phenyl (**L^2b^**), 2-hydroxyl-phenyl (**L^2c^**), 4-methoxyl-phenyl (**L^2d^**), 4-carboxyl-phenyl (**L^2e^**), 4-(methylsulfonyl)phenyl (**L^2f^**), 4-nitrophenyl (**L^2g^**) or furan-2-yl (**L^2h^**), which were characterized by IR, elemental analysis and X-ray single-crystal diffraction. Their antiproliferative potentials against five tumor cell lines were studied. The ability of the complexes to bind to DNA was investigated by spectrophotometric studies and molecular modeling methods.

## 2. Results and Discussion

### 2.1. Synthesis and Characterization

**Figure 1:**
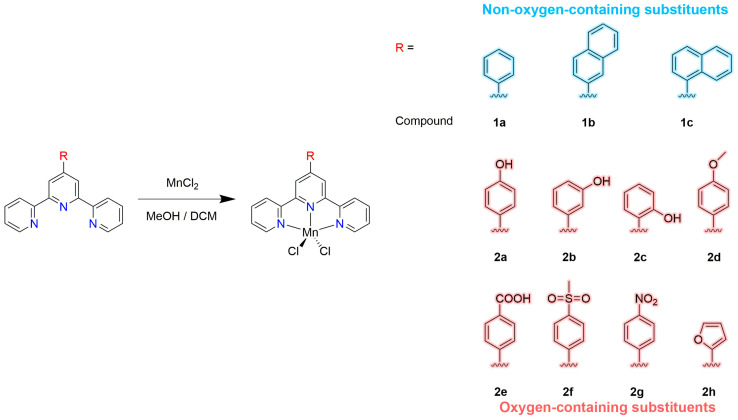
Synthesis of compounds **1a**–**1c** and **2a**–**2h**.

The complexes 4′-phenyl-terpyridine (**L^1a^**), 4′-(naphthalen-2-yl)-2,2′:6′,2″-terpyridine (**L^1b^**), 4′-(naphthalen-1-yl)-2,2′:6′,2″-terpyridine (**L^1c^**), 4′-(4-hydroxyl-phenyl)-2,2′:6′,2″-terpyridine (**L^2a^**), 4′-(3-hydroxyl-phenyl)-2,2′:6′,2″-terpyridine (**L^2b^**), 4′-(2-hydroxyl-phenyl)-2,2′:6′,2″-terpyridine (**L^2c^**), 4′-(4-methoxyl-phenyl)-2,2′:6′,2″-terpyridine (**L^2d^**), 4′-(4-carboxyl-phenyl)-2,2′:6′,2″-terpyridine (**L^2e^**), 4′-(4-(methylsulfonyl)phenyl)-2,2′:6′,2″-terpyridine (**L^2f^**), 4′-(4-nitrophenyl)-2,2′:6′,2″-terpyridine (**L^2g^**) and 4′-(furan-2-yl)-2,2′:6′,2″-terpyridine (**L^2h^**) were obtained using the general established protocol, and the syntheses of the ligands **L^1a^** and **L^2a^–L^2f^** were reported earlier [38,41]. The manganese compounds **1a**–**1c** and **2a**–**2h** (Figure 1) were synthesized by the reaction of **L^1a^**–**L^1c^** and **L^2a^**–**L^2h^** with MnCl_2_·4H_2_O in MeOH/DCM solution, giving yields in the range of 46–73%.

The structures of the target compounds were confirmed by IR, elemental analysis and single-crystal X-ray crystallography. Their IR spectra (Figures S1–S11) display the expected bands [43,64,65]. Namely, multiple bands in the range of 1650–1401 cm^−1^ and 1263–1011 cm^−1^ were observed for the C=C stretch and for in-plane C–H bending vibrations, as well as bands in the range of 911–673 cm^−1^ for out-of-plane C–H bending vibrations. The hydroxyl group of **2a**–**2c** gave a broad band in the range of 3300–3600 cm^−1^ for the O–H stretch, and the methoxyl group of **2d** showed a band at 1240 cm^−1^ for the C–O–C stretch. The carboxyl group of **2e** displayed a broad band in the range of 3400–3600 cm^−1^ with the O–H stretch and a band at 1726 cm^−1^ with the C=O stretch. The sulfonyl group of **2f** gave strong bands at 1308 and 1297 cm^−1^ for SO_2_ stretching, a band at 793 cm^−1^ for the S–O stretch and two bands at 532 and 548 cm^−1^ for SO_2_ bending. The nitro group of **2g** exhibited strong bands at 1519 and 1343 cm^−1^ for NO_2_ stretching, a band at 858 cm^−1^ for the CN stretch and a band at 754 cm^−1^ for CNO bending. The furyl group of **2h** displayed bands at 790 and 782 cm^−1^ for C–H bending vibrations.

### 2.2. Single Crystal X-ray Crystallography

The single-crystal X-ray crystallography of compounds **1a**, **1c**, **2a**–**2c, 2f** and **2g** confirmed their formulation as [MnCl_2_**L^n^**] (n = 1a, 1c, 2a–2c, 2f and 2g), and the manganese cation presented the common square pyramidal geometry. The crystal structures of compounds **1a** and **2c** have been reported [64,66]. Thermal ellipsoid plots and packing diagrams of **1a**, **1c**, **2a**–**2c, 2f** and **2g** are shown in Figure 2 and Figures S12–S24.

Compound **1c** is a mononuclear neutral manganese complex that crystallized in the centric symmetric space group *P*-1, as shown in Figure 1. Each mononuclear ion is coordinated by the three N atoms of the 4′-(naphthalen-1-yl)-2,2′:6′,2″-terpyridine (**L^1c^**) ligand and two chlorine atoms as two auxiliary ligands, therefore forming an irregular square-based pyramid with a N_3_Cl_2_ coordination environment (*τ* = 0.07) [67]. Three hydrogen bonds exist in the structure, involving the chloride atom Cl(1), the hydrogens at the carbon atoms of the terpyridine ligand (C(12) and C(25)) and the chloride atom Cl(2) and the hydrogens at the carbon atoms of the terpyridine ligand (C(27)) with a range of distances in 2.73–2.80 Å. The structure of **1c** shows two kinds of π-ring (Y-H...Cg) interactions, including one between one hydrogen at C27 and the ring formed by C(20)–C(21)–C(22)–C(23)–C(24)–C(25) of the naphthalen-1-yl, and the other one involving another hydrogen atom at C27 and the ring formed by C(16)–C(17)–C(18)–C(19)–C(20)–C(21) of the naphthalen-1-yl with atom-centroid distances (X..Cg) of 3.733(3) and 3.679(3) Å. Due to the specific arrangements of the ligands in **1c**, the compound molecules present two kinds of intermolecular π–π interactions, including one between the two pyridyl rings of the ligand and the other one between the ring formed by Mn(1)–N(1)–C(5)–C(6)–N(2) and one terminal pyridyl ring with centroid distances of 3.641 and 3.687 Å.

Similar to compound **1c**, complexes **1a**, **2a**–**2c**, **2f** and **2g** are also mononuclear species crystallized in the space groups *P*2_1_/*n* for **1a**, *P*2_1_/*n* for **2a**, *P*2_1_/*n* for **2b**, *P*-1 for **2c**, *P*2_1_/*n* for **2f** and *C*2/*c* for **2g**. The asymmetric units of the compounds present half of the molecule in **2g** and one molecule in the other compounds, because of the symmetry of the molecules. The atoms Mn1, N2, C9, C10, C13 and N3 are located on the twofold axis in **2g**. The coordination environment around the manganese ion in these compounds displays an irregular square-based pyramid (*τ* = 0.23 for **1a**, 0.24 for **2a**, 0.26 for **2b**, 0.13 for **2c**, 0.14 for **2f** and 0.08 for **2g**) [67], which results from the penta-coordination of manganese by three nitrogen atoms from the substituted terpyridine ligands and two chloride atoms as the auxiliary ligands in a monodentate mode. The average contacts between the central metal ion and the chloride atoms (Mn–Cl) is 2.360 Å in **1a**, being longer than those in **1c** (2.358 Å), **2c** (2.352 Å), **2f** (2.341 Å) and **2g** (2.326 Å), but shorter than those in **2a** (2.363 Å) and **2b** (2.365 Å). Such bond lengths are not only affected by the electrophilicity of the substituents, but also by other factors, including hydrogen bonds or even spatial environments determined by the positions of the substituent groups at the phenyl rings.

Hydrogen bonds are observed in compounds **1a**, **2a**–**2c**, **2f** and **2g**, and the bond lengths are 2.81, 2.73, 2.80 and 2.78 Å for **1a**; 2.33 Å for **2a**; 2.31, 2.81 and 2.82 Å for **2b**; 2.02, 1.83 and 1.82 Å for **2c**; 2.57, 2.47, 2.45 and 2.68 Å for **2f**; and 2.82, 2.59 and 2.60 Å for **2g**. Several π-ring interactions exist in the structure of compound **9,** but there was no such interaction in **2a**–**2c** and **2g**. In **2f**, there are two kinds of π-ring (Y-X…Cg) interactions, one between an oxygen at S(1) of the methylsulfonyl and the rings formed by Mn(1)–N(1)–C(5)–C(6)–N(2) with atom-centroid distances (X…Cg) of 3.399 Å, and another between a hydrogen at C(22) of the methylsulfonyl and the terminal pyridyl ring N(1)–C(1)–C(2)–C(3)–C(4)–C(5) with atom-centroid distances (X…Cg) of 2.990 Å. In these structures, the different packing patterns lead to the different π–π stackings: two in **1a**, **2a** and **2b** (in the range of 3.538–3.646, 3.572–3.736 and 3.561–3.718 Å, respectively), three in **2c** (3.684–3.773 Å) and one for **2f** and **2g** (with a distance of 3.610 Å and 3.668 Å, respectively), involving the ring formed by Mn(1)–N(1)–C(5)–C(6)–N(2) and a terminal pyridyl ring or a terminal and a middle pyridyl ring.

### 2.3. Antiproliferative Properties against Tumor Cells

**Figure 3:**
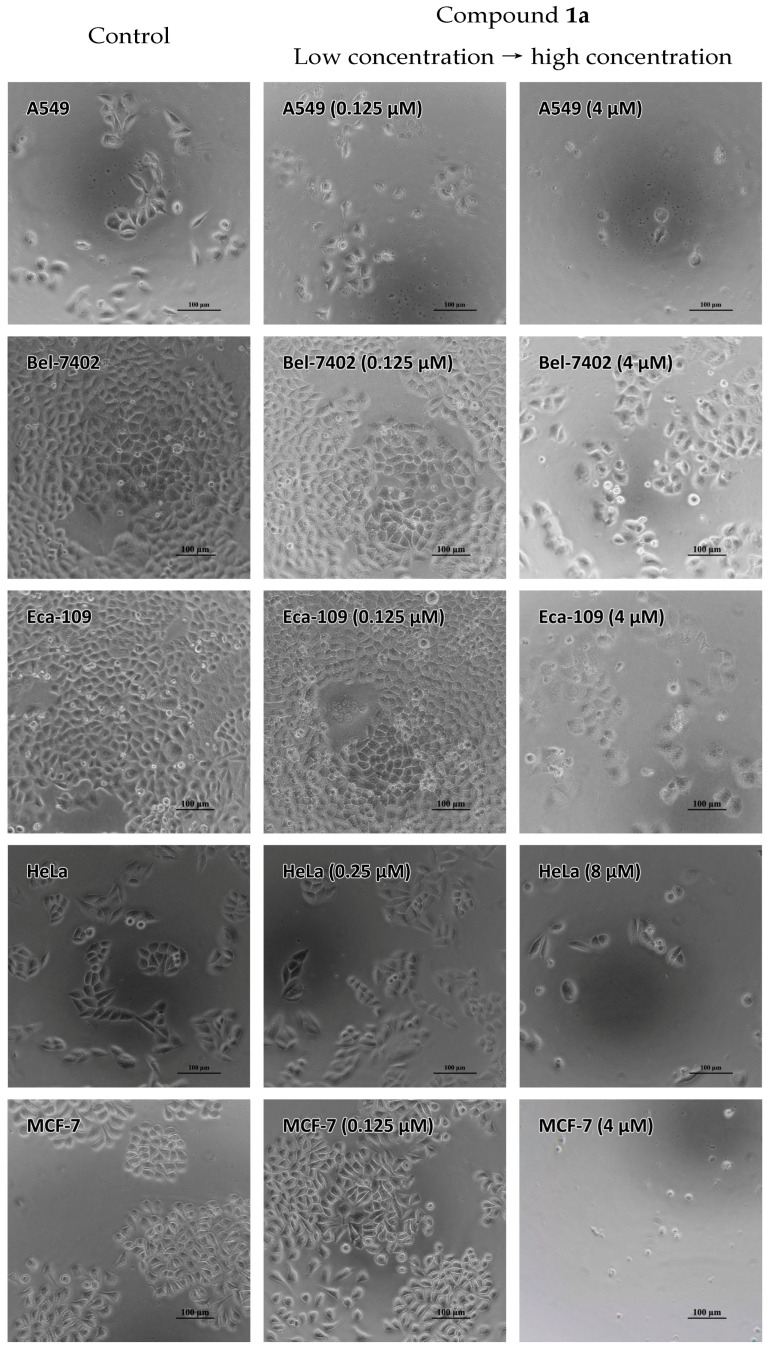
Microscopic photographs of the A-549, Bel-7402, Eca-109, HeLa and MCF-7 cancer cells treated with increasing concentrations of compound **1a**.

Five human carcinoma cell lines, including A549, Bel-7402, Eca-109, HeLa and MCF-7, were treated with various concentrations of compounds **1a**–**1c** and **2a**–**2h** (0.125–4 µM for A-549, Bel-7402, Eca-109 and MCF-7 cells or 0.25–8µM for HeLa cell line) to evaluate the in vitro antiproliferative activities of the eleven compounds. Figure 3 shows the live-cell images treated with different concentrations of compound **1a**, and the microscopic photographs of the five cells treated with various concentrations of compounds **1b**, **1c** and **2a**–**2h** were collected in the supporting information as Figures S25–S34. With the increasing concentrations of the compounds, the number of cancer cells observed by microscope decreased significantly. Notably, different kinds of cells respond differently to these compounds. For A549 cells, slight swelling of the cell body to nuclei shrinkage and blurring of cell boundaries were observed as the concentrations of the compounds increased, which showed the death of the cells. Regarding the Bel-7402 cell line, the obvious swelling of the cell body was observed at low concentrations of the compounds. As the concentrations of the compounds increased, a further increase in the volume of the single cell, followed by shrinkage of the nuclei and the fragmentation of the cells can be observed, suggesting the necrosis of Bel-7402 cells. Swelling of the cell body at low concentrations of the tested compounds was also observed for Eca-109 cells, with cell contraction, blurring of cell boundaries and fragmentating of the cell body being observed at high concentrations of the compounds. At low concentrations, there was no significant change in the cell morphology of HeLa cells. However, as the concentration of the compounds increased, some cells changed from a fusiform to a spherical shape with shrinkage of the nucleus. Significant swelling of the cell body was also observed in MCF-7 cells, but the higher concentration of the compounds led to cell contraction, the blurring of cell boundaries and nuclei shrinkage.

**Figure 4:**
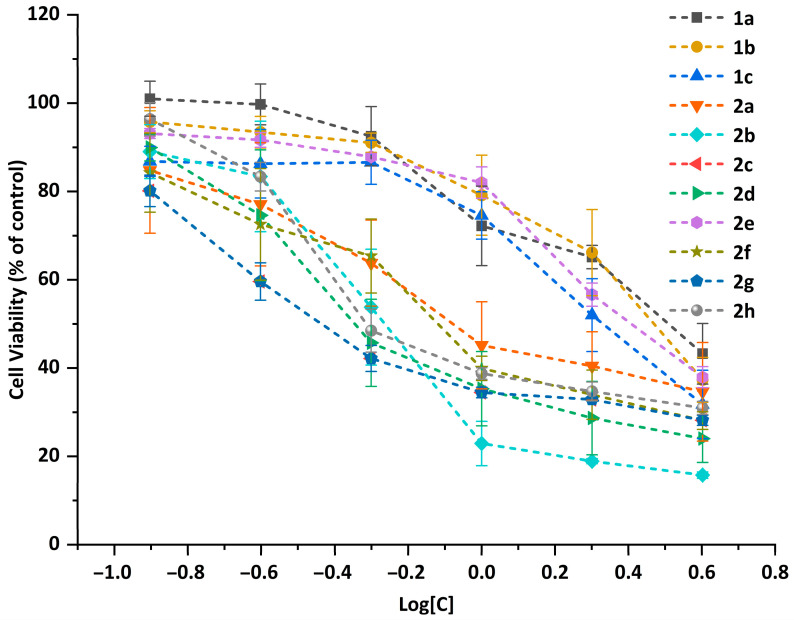
Plots of cell viability vs. the concentration of compounds **1a**–**1c** and **2a**–**2h** in different concentrations against Eca-109 cell line.

Cell viability of the five cell lines was determined by the CCK-8 assay. The plots of the cell viability vs. the concentration of compounds **1a**–**1c** and **2a**–**2h** against the Eca-109 cell line (Figure 4) showed that all the compounds exhibited a strong antiproliferative effect against the selected cell lines. The viability of Eca-109 cells decreases with the increase in the compound concentrations, exhibiting a dose-dependent manner. For the A549, Bel-7402, HeLa and MCF-7 cell lines, similar trends were observed and are shown in the curves of the antiproliferative activities (Figures S35–S38).

The half-maximal inhibitory concentrations (IC_50_) of the eleven compounds against the carcinoma cells were calculated and are listed in Table 1. The IC_50_ values of compounds **1a**–**1c** and **2a**–**2h** against the A549, Bel-7402, Eca-109, HeLa and MCF-7 cell lines were much lower than the widely used anti-tumor drug cisplatin. In the results, compound **2d** presented the lowest IC_50_ value against both A549 and HeLa cells; the values were 0.281 μM and 0.356 μM, respectively. Compound **2h** showed the best results (0.523 μM) on Bel-7402 cells, **2g** gave the lowest IC_50_ value (0.514 μM) against Eca-109 cells and **2c** had the lowest value (0.249 μM) on MCF-7 cells.

**Figure 5:**
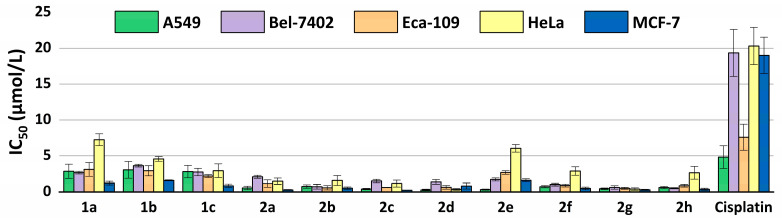
IC_50_ values (μM) from the dose-response assay of compounds **1a**–**1c** and **2a**–**2h** and the reference compound cisplatin in the A549, Bel-7402, Eca-109, HeLa and MCF-7 cell lines, after an incubation time of 48 h. The results shown are means ± SD of quadruplicate experiments.

A comparison of the antiproliferative activity of the compounds against different cell lines is shown in Figure 5. Among the five cell lines, A549, Bel-7402, MCF-7 and Eca-109 were more susceptible, and HeLa was more tolerant for the tested compounds. Among the eleven compounds, **2g** with the nitro group performed the best antiproliferative activities against the five cell lines on the whole, usually showing much lower IC_50_ values in the tested cells than the others. Since the compounds differ from each other only in the 4-substituents at the terpyridyl group, the relationship between the anticancer activity of all the compounds and their structures was further analyzed.

**Figure 6:**
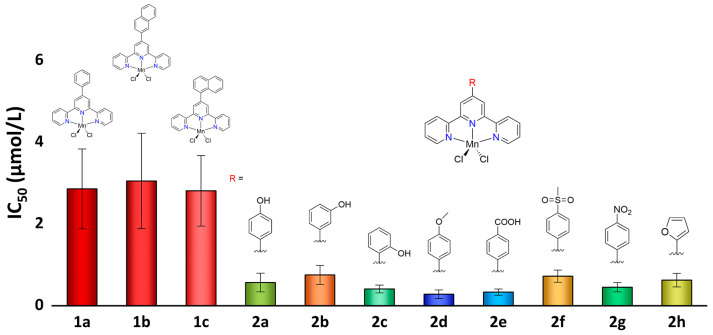
IC_50_ values (μM) of compounds **1a**–**1c** and **2a**–**2h** against the A549 cell line and the structures of the compounds.

As shown in Figure 6, when A549 cancer cells were used, the anticancer activity of the 2-naphthyl and 1-naphthyl-substituted terpyridine compounds was close to that of 4′-phenyl-terpyridine. When the substituent at terpyridine was an oxygen-containing group, more than four times higher anticancer activities were obtained in comparison with those of 4′-phenyl-terpyridine compounds. The electronegativity and stereochemical effects of the oxygen-containing substituents are critical for the anticancer activity of the complexes; they showed an increase in bioactivity with the increase in the electronegativity of the substituent and a decrease in bioactivity with the increase in steric hindrance, resulting in anticancer activity as 4-OMe-Ph– > 4-COOH-Ph– > 2-OH-Ph– > 4-NO_2_-Ph– > 4-OH-Ph > 4-(furan-2-yl)– > 4-Ms-Ph– > 3-OH-Ph. For the four other cell lines, Bel-7402, Eca-109, HeLa and MCF-7, the compounds with oxygen-containing substituents showed significantly better anticancer activity.

**Figure 7:**
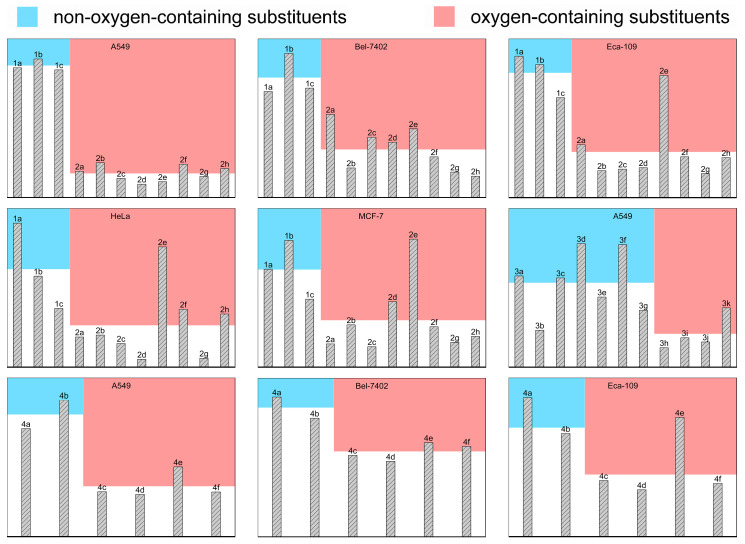
The in vitro anticancer activity of substituted 2,2′:6′,2″-terpyridine(tpy) complexes **1a**–**1c**, **2a**–**2h**, **3a**–**3k [65]** and **4a**–**4f [44]** against different cancer cell lines, including A549, Bel-7402, Eca-109, HeLa and MCF-7. The heights of the blue and red blocks on the background represent the average performance of the anticancer activity of the non-oxygen-containing substituents and oxygen-containing substituent-modified complexes, respectively. Here, **3a** = [Cu(tpy)Cl_2_], **3b** = [Cu(4′-(4′-cyano-phenyl)-tpy)Cl_2_], **3c** = [Cu(4′-(4′-iodo-phenyl)-tpy)Cl_2_], **3d** = [Cu(4′-(4′-bromo-phenyl)-tpy)Cl_2_], **3e** = [Cu(4′-(4′-chloro-phenyl)-tpy)Cl_2_], **3f** = [Cu(4′-(4′-fluoro-phenyl)-tpy)Cl_2_], **3g** = [Cu(4′-(*p*-hydroxyl-phenyl)-tpy)Cl_2_], **3h** = [Cu(4′-(*m*-hydroxyl-phenyl)-tpy)Cl_2_], **3i** = [Cu(4′-(*o*-hydroxyl-phenyl)-tpy)Cl_2_], **3j** = [Cu(4′-(methoxyl-phenyl)-tpy)Cl_2_], **3k** = [Cu(4′-(4′-methylsulfonyl-phenyl)-tpy)Cl_2_], **4a** = [Zn(4′-(4′-methyl-phenyl)-tpy)Br_2_], **4b** = [Zn(4′-(4′-methyl-phenyl)-tpy)I_2_], **4c** = [Zn(4′-(4′-methylsulfonyl-phenyl)-tpy)Br_2_], **4d** = [Zn(4′-(4′-methylsulfonyl-phenyl)-tpy)I_2_], **4e** = [Zn(4′-(4′-methoxyl-phenyl)-tpy)Br_2_], **4f** = [Zn(4′-(4′-methoxyl-phenyl)-tpy)I_2_].

In order to clarify the effect of oxygen-containing substituent modification on the anticancer activity of terpyridine complexes, we summarized the anticancer activities of a total of 28 substituted terpyridine complexes against different cancer cells in Figure 7 from this work and previously reported works [44,65]. When the average activity of the non-oxygen-containing substituent-modified terpyridine complexes is compared with that of the terpyridine complexes modified with oxygen-containing substituents, we can clearly find that oxygen-containing substituents brought a significant increase in anticancer activity. Although the electronegativity, steric hindrance and ability to form hydrogen bonds of substituents can also affect the order of anticancer activity of compounds, modification with oxygen-containing substituents is still an effective strategy to enhance the anticancer activity of terpyridine complexes.

### 2.4. Circular Dichroism Spectroscopic Studies

CD spectroscopy is one of the most sensitive techniques for monitoring structural changes in DNA in solution [68,69,70]. The reliance on CD spectroscopy to study DNA conformations has stemmed from the sensitivity and ease of CD measurements, the nondestructive nature of such measurements, the fact that conformations can be studied in solution and the requirement for relatively small amounts of material [71].

**Figure 8:**
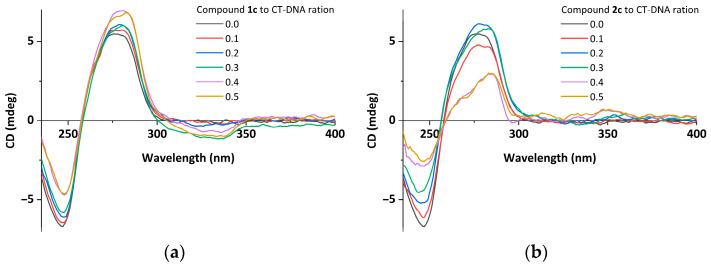
(**a**) Circular dichroism spectra of CT-DNA (6.0 × 10^−4^ mol/L) in the presence or absence of compound **1a** in Tris-HCl buffer (pH 7.2) at 20 °C. (**b**) Circular dichroism spectra of CT-DNA (6.0 × 10^−4^ mol/L) in the presence or absence of compound **2c** in Tris-HCl buffer (pH 7.2), at 20 °C.

The CD spectra of CT-DNA with increased concentrations of compounds **1a**–**1c** and **2a**–**2h** were measured and are shown in Figure 8 and Figures S39–S47. For reference, when no compound was added, the CD spectrum of CT-DNA showed two peaks at 277 nm and 246 nm, respectively. The two bands are the known features of a right-handed B-form DNA. Specifically, the positive band at 277 nm is due to base stacking, and the negative band at 246 nm is from the right-handed helicity. When the different concentrations of the compounds were added and incubated with CT-DNA, obvious changes in both positive and negative bands were observed. All the CD spectral bands of the DNA with the different concentrations of the compounds are tabulated in Table S1.

As the concentrations of the compounds increase to 300 µmol/L, the intensities of the positive bands decrease by 12–62% compared to CT-DNA, with slight shifts in the *λ*_max,_ and the intensities of the negative bands decrease by 5–46%. Notably, when compound **2h** at a concentration of 60 µmol/L interacts with CT-DNA, the band attributed to base stacking (at 277 nm) shows a 10% increase in positive ellipticity with no considerable shift in its position. For compound **1c**, the positive band shows a 26% increase as the compound concentration increases, which may be due to the participation and facilitation of the compounds with high planarity in the π–π stacking of the base pairs of DNA.

The changes in the ellipticity and wavelength of the CD signals around 277 nm are important for elucidating their ability to intercalate between DNA base pairs [72,73]. By comparing the hypochromism caused by compounds **1a**–**1c** and **2a**–**2h** at the same ratio, compound **2c** exhibited the strongest intercalation ability. This indicates that the different substituents at the terpyridine significantly affect the interaction between the compound and DNA, and the *o*-hydroxyl group seems to promote the intercalation.

### 2.5. Molecular Docking Studies

Molecular docking techniques have shown great promise as a new tool in the discovery of novel small-molecule drugs for predicting the plausible interactions between the drug and nucleic acid in a non-covalent fashion. Most anti-tumor drugs have functions by incorporating into the base pairs of the DNA of tumor cells to interrupt their replication and transcription [74,75]. The syntheses of important proteins is terminated or disrupted, resulting in the termination of cell division and growth, cell swelling, cell necrosis, etc., which was observed in this study. In order to explore the mechanism of their anticancer activity, the DNA interaction with the compounds was studied using the molecular modeling method.

#### 2.5.1. Molecular Docking with DNA

**Figure 9:**
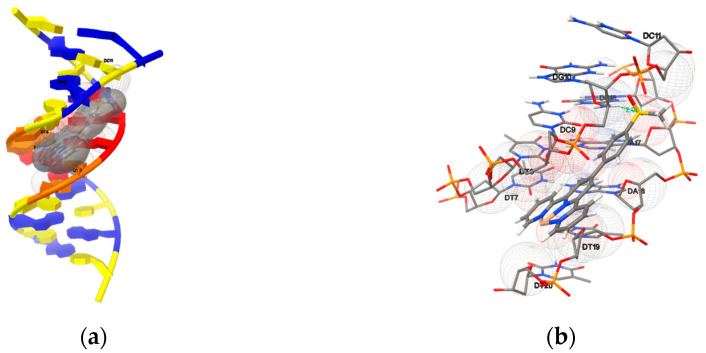
(**a**) The most favorable orientation and (**b**) enlarged view of compound **2f** bound with the minor groove of the B-DNA (PDB ID: 1BNA). The formed hydrogen bond and distance have been marked.

In this study, the AutoDock program was used to examine the compound–DNA interactions by investigating the potential binding modes and calculating the binding energies. At first, rigid molecular docking studies of compounds **1a**–**1c** and **2a**–**2h** with the DNA duplex of sequence d(CGCGAATTCGCG)_2_ dodecamer (PDB ID: 1BNA) were performed in order to predict the binding site along with the preferred orientation of the ligand. Detailed simulations of the compound–DNA interactions were carried out and are presented in Figure 9 and Figures S48–S57. The free energies of the binding were calculated and are shown in Table 2.

It was observed that the ligand fits into the minor groove perfectly, involving outside edge interactions without disrupting the double-helical structure of the DNA. The bindings were stabilized by van der Waals interaction and hydrophobic contacts with DNA functional groups, which define the stability of groove, and the binding energies were between –8.55 and –10.86 kcal mol^−1^. Hydrogen bonds were found between the compounds and the DNA, and the detailed bond distances and bond energies are listed in Table 3. Compounds **2a**–**2c**, **2f** and **2g** formed hydrogen bonds with B-DNA benefitting from the presence of the substituent groups, whereas the other compounds showed no hydrogen bond formation in their most favorable orientations.

**Figure 10:**
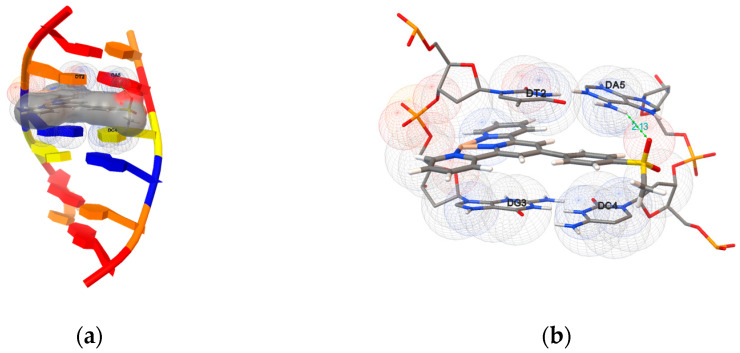
(**a**) The most favorable orientation and (**b**) enlarged view of compound **2f** intercalating with the DNA (4JD8). The formed hydrogen bond and distance have been marked.

Furthermore, compounds **1a**–**1c** and **2a**–**2h** were docked onto an oligonucleotide (ds(ATGCAT)_2_, PDB ID: 4JD8) to explore the potential binding mode and energy. The binding energies of the eleven compounds were calculated and are listed in Table 2. They were in the range between −8.07 and −10.23 kcal mol^−1^, and the detailed docking poses are presented in Figure 10 and Figures S58–S67. The results indicate that the docked compounds intercalate into the base pairs of the DNA, involving π–π stacking, van der Waals interaction and hydrophobic and hydrogen bonding. Compounds **2a**–**2c**, **2f** and **2g** formed hydrogen bonds with the oligonucleotide by the hydroxyl group. No hydrogen bond was detected in the most favorable poses of **1a**–**1c**, **2d**, **2e** and **2h**.

#### 2.5.2. Molecular Docking with Topoisomerase I

**Figure 11:**
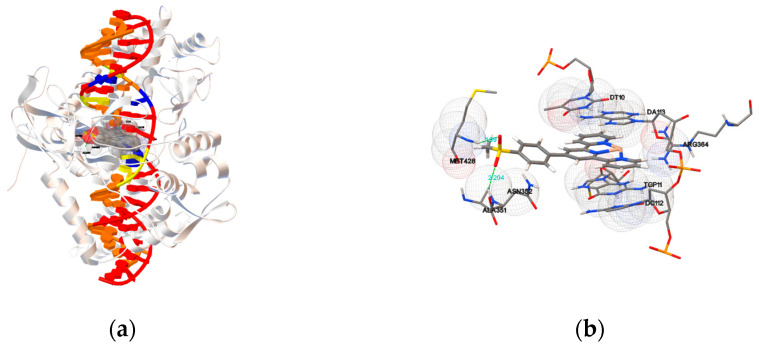
(**a**) Molecular docking models and (**b**) enlarged view of **2f** in the active site of DNA–Topo I complex (PDB ID: 1SC7). The formed hydrogen bond and distance have been marked.

To elucidate the interaction and locate the exact binding site between the compounds **1a**–**1c** and **2a**–**2h** and Topo-I, molecular docking studies were performed using the Human-DNA Topo-I complex (PDB ID: 1SC7). As seen in Figure 11 and Figures S68–S77, the terpyridyl group was intercalated into the base pairs of the DNA, and the substituents at the terpyridyl group formed hydrogen bonds with the residues of Topo-I. An in silico molecular docking experiment revealed that the docked compounds fit into the Topo I-DNA complex perfectly and resulted in the binding energy between –9.06 and −12.34 kcal mol^−1^. No hydrogen bond was detected in the most favorable pose of compounds **1a**–**1c**, **2d**, **2e** and **2h**. Meanwhile, compounds **2a**–**2c**, **2f** and **2g** showed a binding energy value from –9.58 to −12.34 kcal mol^−1^ with one or two hydrogen bonds. For compound **2a**, the O–H formed a hydrogen bond with an oxygen of the ASN722, and the bond length was 2.133 Å. The hydroxyl of compound **2b** formed two hydrogen bonds with the ASN722, and the O–H of **6** formed a hydrogen bond with an oxygen of the phosphate. For compound **2f**, one N–H at the MET428 and one N–H at the ALA351 interacted with the two oxygen atoms at the methylsulfonyl group to form two hydrogen bonds with bond lengths of 1.990 and 2.204 Å, respectively. For **2g**, one N–H at the LYS425 interacted with the oxygen at the nitro group and formed a hydrogen bond with a bond length of 1.877 Å. The details of the hydrogen bonds have been summarized in Table 3. The results proved that the substituents at the terpyridyl group contribute to forming a stable complex in the DNA–Topo I active site through π–π stacking with the purine ring of DNA, van der Waals, hydrophobic bonding and hydrogen bonding with the residues of Topo-I. It can be inferred from the present docking studies that some subtle change in the structure of the drug molecule alters the ligand-binding domain in the drug target. These phenomena are very interesting, as well as desirable for drug designing, because repeated application of the same drug/compound leads to the development of resistance to the action of the drug, due to unavoidable conformational modifications in the drug target.

## 3. Methods and Materials

### 3.1. Chemicals and Reagents

All common reagents employed in this work were of analytical grade.

### 3.2. Instruments and Apparatus

The infrared spectra were obtained with a Thermo Scientific Nicolet iS10 spectrophotometer, and elemental analyses (C, H, N) were performed on an Elementar vario EL cube.

### 3.3. Synthesis of the Compounds

The methanol solution of MnCl_2_ was added to dichloromethane solutions of the corresponding ligands, and the mixture was stirred for 24 h. The separation was performed by filtering the compound powders from the mother solution and drying them in a desiccator. Recrystallization from a methanol/acetonitrile solution upon slow evaporation led to the formation of crystals that were suitable for X-ray analysis of complexes **1a**, **1c**, **2a**–**2c, 2f** and **2g**.

[MnCl_2_**L^1a^**] (**1a**). Orange crystals. Yield: 0.30 g, 69%. Anal. calcd for C_21_H_15_Cl_2_N_3_Mn: C, 57.96; H, 3.47; N, 9.66%. Found: C 56.55, H 3.44, N 9.47%. IR (KBr disc, cm^−1^, s = strong, m = medium, w = weak): 3052 (m, *ν*_C–H_), 2988 (m, *ν*_C–H_), 2900 (w, *ν*_C–H_), 1600 (s, *ν*_C=C_), 1549 (m, *ν*_C=C_), 1475 (s, *ν*_C=C_), 1434 (m, *ν*_C=C_), 1401 (m, *ν*_C=C_), 1303 (m), 1232 (m, *β*_C–H_), 1163 (m, *β*_C–H_), 1077 (m, *β*_C–H_), 1066 (m, *β*_C–H_), 1014 (m, *β*_C–H_), 898 (m, *γ*_C–H_), 886 (m, *γ*_C–H_), 832 (m, *γ*_C–H_), 791 (m, *γ*_C–H_), 773 (m, *γ*_C–H_), 746 (s, *γ*_C–H_), 731 (m, *γ*_C–H_), 695 (m, *γ*_C–H_), 657 (m), 638 (m), 619 (m), 581 (m) and 560 (m).

[MnCl_2_**L^1b^**] (**1b**). Yield: 0.27 g, 56%. Anal. calcd for C_25_H_17_Cl_2_N_3_Mn·0.5H_2_O: C, 60.75; H, 3.67; N, 8.50%. Found: C 60.71, H 3.45, N 8.42%. IR (KBr disc, cm^−1^): 3057 (m, *ν*_C–H_), 1608 (s, *ν*_C=C_), 1570 (m, *ν*_C=C_), 1545 (m, *ν*_C=C_), 1475 (s, *ν*_C=C_), 1440 (m, *ν*_C=C_), 1417 (m, *ν*_C=C_), 1250 (m, *β*_C–H_), 1160 (m, *β*_C–H_), 1131 (m, *β*_C–H_), 1067 (m, *β*_C–H_), 1012 (s, *β*_C–H_), 896 (m, *γ*_C–H_), 869 (m, *γ*_C–H_), 824 (m, *γ*_C–H_), 794 (s, *γ*_C–H_), 785 (m, *γ*_C–H_), 769 (m, *γ*_C–H_), 741 (s, *γ*_C–H_), 728 (m, *γ*_C–H_), 686 (m, *γ*_C–H_), 657 (m), 638 (m), 604 (m), 566 (m) and 556 (m).

[MnCl_2_**L^1c^**] (**1c**). Yellow crystals. Yield: 0.28 g, 58%. Anal. calcd for C_25_H_17_Cl_2_N_3_Mn: C, 61.88; H, 3.53; N, 8.66%. Found: C 61.64, H 3.49, N 8.53%. IR (KBr disc, cm^−1^): 3099 (m, *ν*_C–H_), 3065 (m, *ν*_C–H_), 3022 (m, *ν*_C–H_), 1606 (m, *ν*_C=C_), 1595 (m, *ν*_C=C_), 1567 (m, *ν*_C=C_), 1541 (m, *ν*_C=C_), 1468 (m, *ν*_C=C_), 1439 (m, *ν*_C=C_), 1413 (m, *ν*_C=C_), 1293 (m), 1244 (m, *β*_C–H_), 1011 (m, *β*_C–H_), 911 (m, *γ*_C–H_), 886 (m, *γ*_C–H_), 868 (m, *γ*_C–H_), 802 (s, *γ*_C–H_), 791 (s, *γ*_C–H_), 781 (s, *γ*_C–H_), 741 (m, *γ*_C–H_), 734 (m, *γ*_C–H_), 689 (w, *γ*_C–H_), 664 (m), 651 (m), 639 (m), 632 (m) and 589 (m).

[MnCl_2_**L^2a^**] (**2a**). Orange crystals. Yield: 0.30 g, 66%. Anal. calcd for C_21_H_15_Cl_2_N_3_Mn·H_2_O·CH_2_Cl_2_: C, 47.68; H, 3.46; N, 7.58%. Found: C 47.91, H 3.16, N 7.28%. IR (KBr disc, cm^−1^): 3419 (m, *ν*_O–H_), 3273 (m, *ν*_C–H_), 1598 (s, *ν*_C=C_), 1586 (s, *ν*_C=C_), 1573 (m, *ν*_C=C_), 1547 (m, *ν*_C=C_), 1522 (m, *ν*_C=C_), 1475 (s, *ν*_C=C_), 1442 (s, *ν*_C=C_), 1410 (m, *ν*_C=C_), 1362 (m, *δ*_O–H_), 1279 (m), 1245 (s, *ν*_C=C_), 1218 (m, *β*_C–H_), 1182 (m, *ν*_C–OH_), 1120 (m, *β*_C–H_), 1068 (m, *β*_C–H_), 1012 (m, *β*_C–H_), 850 (m, *γ*_C–H_), 824 (m, *γ*_C–H_), 790 (m, *γ*_C–H_), 748 (m, *γ*_C–H_), 730 (m, *γ*_C–H_), 658 (m), 638 (m), 623 (m), 573 (m).

[MnCl_2_**L^2b^**] (**2b**). Brown crystals. Yield: 0.28 g, 62%. Anal. calcd for C_21_H_15_Cl_2_N_3_OMn·2H_2_O·CH_2_Cl_2_: C, 46.18; H, 3.70; N, 7.34%. Found: C 46.03, H 3.63, N 7.05%. IR (KBr disc, cm^−1^): 3419 (s, *ν*_O–H_), 3285 (m, *ν*_C–H_), 2935 (m, *ν*_C–H_), 2841 (m, *ν*_C–H_), 1650 (m, *ν*_C=C_), 1599 (s, *ν*_C=C_), 1571 (m, *ν*_C=C_), 1545 (m, *ν*_C=C_), 1520 (m, *ν*_C=C_), 1475 (s, *ν*_C=C_), 1416 (s, *ν*_C=C_), 1364 (m, *δ*_O–H_), 1283 (m), 1243 (s, *ν*_C=C_), 1187 (m, *ν*_C–OH_), 1164 (m, *β*_C–H_), 1103 (m, *β*_C–H_), 1070 (m, *β*_C–H_), 1012 (s, *β*_C–H_), 892 (m, *γ*_C–H_), 839 (m, *γ*_C–H_), 791 (s, *γ*_C–H_), 781 (s, *γ*_C–H_), 729 (m, *γ*_C–H_), 685 (m, *γ*_C–H_), 658 (m), 638 (m) and 576 (m).

[MnCl_2_**L^2c^**] (**2c**). Orange crystals. Yield: 0.27 g, 60%. Anal. calcd for C_21_H_15_Cl_2_N_3_OMn·H_2_O·CH_2_Cl_2_: C, 47.68; H, 3.46; N, 7.58%. Found: C 47.41, H 3.16, N 7.76%. IR (KBr disc, cm^−1^): 3478 (s, *ν*_O–H_), 2960 (m, *ν*_C–H_), 2926 (m, *ν*_C–H_), 2852 (m, *ν*_C–H_), 1608 (s, *ν*_C=C_), 1598 (s, *ν*_C=C_), 1570 (m, *ν*_C=C_), 1546 (m, *ν*_C=C_), 1475 (m, *ν*_C=C_), 1456 (m, *ν*_C=C_), 1411 (s, *ν*_C=C_), 1307 (m), 1263 (m, *ν*_C=C_), 1244 (m, *ν*_C=C_), 1163 (m, *β*_C–H_), 1097 (m, *ν*_C–OH_), 1013 (s, *β*_C–H_), 889 (s, *γ*_C–H_), 859 (m, *γ*_C–H_), 789 (s, *γ*_C–H_), 764 (s, *γ*_C–H_), 750 (m, *γ*_C–H_), 731 (m, *γ*_C–H_), 657 (m), 637 (m), 621 (m) and 547 (m).

[MnCl_2_**L^2d^**] (**2d**). Yield: 0.34 g, 73%. Anal. calcd for C_22_H_17_Cl_2_N_3_OMn·H_2_O: C, 54.68; H, 3.96; N, 8.70%. Found: C 54.28, H 3.65, N 8.56%. IR (KBr disc, cm^−1^): 3058 (m, *ν*_C–H_), 2937 (m, *ν*_C–H_), 2840 (m, *ν*_C–H_), 1598 (s, *ν*_C=C_), 1545 (m, *ν*_C=C_), 1520 (m, *ν*_C=C_), 1475 (s, *ν*_C=C_), 1433 (m, *ν*_C=C_), 1406 (m, *ν*_C=C_), 1363(m, *δ*_C–H_), 1309 (m), 1285 (m), 1240 (s, *ν*_C–O–C_), 1186 (m, *β*_C–H_), 1240 (m, *β*_C–H_), 1240 (m, *β*_C–H_), 1186 (m, *β*_C–H_), 1069 (m, *β*_C–H_), 1013 (m, *β*_C–H_), 891 (m, *γ*_C–H_), 839 (m, *γ*_C–H_), 791 (s, *γ*_C–H_), 748 (m, *γ*_C–H_), 729 (m, *γ*_C–H_), 658 (m), 639 (m) and 576 (m).

[MnCl_2_**L^2e^**] (**2e**). Yield: 0.23 g, 48%. Anal. calcd for C_22_H_15_Cl_2_N_3_O_2_Mn·H_2_O·CH_2_Cl_2_: C, 47.45; H, 3.29; N, 7.22%. Found: C 47.39, H 3.25, N 7.55%. IR (KBr disc, cm^−1^): 3445 (s, *ν*_OH_), 2987 (m, *ν*_C–H_), 2970 (m, *ν*_C–H_), 2901 (m, *ν*_C–H_), 1726 (m, *ν*_C=O_), 1609 (m, *ν*_C=C_), 1598 (s, *ν*_C=C_), 1571 (m, *ν*_C=C_), 1544 (m, *ν*_C=C_), 1476 (s, *ν*_C=C_), 1429 (s, *ν*_C=C_), 1397 (m, *δ*_O–H_), 1251 (m, *ν*_C–O_), 1187 (m, *β*_C–H_), 1121 (m, *β*_C–H_), 1105 (m, *β*_C–H_), 1068 (s, *β*_C–H_), 1013 (s, *β*_C–H_), 893 (m, *γ*_C–H_), 857 (m, *γ*_C–H_), 795 (s, *γ*_C–H_), 769 (s, *γ*_C–H_), 729 (m, *γ*_C–H_), 694 (m, *γ*_C–H_), 659 (m) and 638 (m).

[MnCl_2_**L^2f^**] (**2f**). Orange crystals. Yield: 0.28 g, 55%. Anal. calcd for C_22_H_17_Cl_2_N_3_O_2_SMn·H_2_O·1.5CH_2_Cl_2_: C, 42.85; H, 3.37; N, 6.38%. Found: C 42.56, H 3.07, N 6.33%. IR (KBr, cm^−1^): 3058 (w, *ν*_C–H_), 2936 (w, *ν*_C–H_), 2908(w, *ν*_C–H_), 2841(w, *ν*_C–H_), 1598 (s, *ν*_C=C_), 1520 (m, *ν*_C=C_), 1475 (s, *ν*_C=C_), 1433 (m, *ν*_C=C_), 1407 (m, *ν*_C=C_), 1308 (s, *ν*_SO2_), 1297 (s, *ν*_SO2_), 1241 (m, *β*_C–H_), 1187 (m, *β*_C–H_), 1147 (s, *ν*_SO2_), 1092 (m, *β*_C–H_), 1069 (m, *β*_C–H_), 1012 (m, *β*_C–H_), 965 (m, *γ*_C–H_), 890 (m, *γ*_C–H_), 835 (m, *γ*_C–H_), 801 (s, *γ*_C–H_), 793 (s, *ν*_S–O_), 768 (m, *γ*_C–H_), 729 (m, *γ*_C–H_), 673 (m, *γ*_C–H_), 657 (m), 638 (m), 577 (s, *δ*_SO2_) and 549 (s, *δ*_SO2_).

[MnCl_2_**L^2g^**] (**2g**). Orange crystals. Yield: 0.22 g, 46%. Anal. calcd for C_21_H_14_Cl_2_N_4_O_2_Mn·H_2_O: C, 50.63; H, 3.24; N, 11.25%. Found: C 50.90, H 3.09, N 11.19%. IR (KBr disc, cm^−1^): 3059 (m, *ν*_C–H_), 2937 (w, *ν*_C–H_), 2840 (m, *ν*_C–H_), 1597 (s, *ν*_C=C_), 1571 (m, *ν*_C=C_), 1546 (m, *ν*_C=C_), 1519 (s, *ν*_NO2_), 1475 (s, *ν*_C=C_), 1431 (m, *ν*_C=C_), 1343 (s, *ν*_NO2_), 1241 (m, *β*_C–H_), 1187 (m, *β*_C–H_), 1163 (m, *β*_C–H_), 1069 (m, *β*_C–H_), 1013 (s, *β*_C–H_), 892 (m, *γ*_C–H_), 858 (m, *γ*_CN_), 819 (m, *γ*_C–H_), 791 (s, *γ*_C–H_), 754 (m, *γ*_CNO_), 730 (m, *γ*_C–H_), 658 (m), 638 (m) and 576 (m).

[MnCl_2_**L^2h^**] (**2h**). Yield: 0.22 g, 52%. Anal. calcd for C_19_H_13_Cl_2_N_3_OMn·1.5H_2_O: C, 50.47; H, 3.57; N, 9.29%. Found: C 50.36, H 3.46, N 9.16%. IR (KBr disc, cm^−1^): 3094 (m, *ν*_C–H_), 3060 (m, *ν*_C–H_), 3032 (m, *ν*_C–H_), 1615 (s, *ν*_C=C_), 1600 (s, *ν*_C=C_), 1544 (m, *ν*_C=C_), 1589 (s, *ν*_C=C_), 1459 (m, *ν*_C=C_), 1427 (s, *ν*_C=C_), 1383 (m), 1254 (m, *β*_C–H_), 1231 (m, *β*_C–H_), 1160 (m, *β*_C–H_), 1072 (m, *β*_C–H_), 1030 (m, *β*_C–H_), 1012 (s, *β*_C–H_), 1003 (s, *β*_C–H_), 925 (m), 883 (m, *γ*_C–H_), 839 (m, *γ*_C–H_), 790 (s, *δ*_C–H_), 782 (s, *δ*_C–H_), 743 (s, *γ*_C–H_), 727 (s, *γ*_C–H_), 682 (s, *γ*_C–H_), 654 (m), 638 (m) and 584 (m).

### 3.4. Crystallography

Compound **1c** was initially crystallized from acetonitrile, and the crystal structure was found to contain solvent molecules. For cell viability and DNA interaction studies, **1c** was re-synthesized from MeOH/DCM to prevent the incorporation of solvent molecules into the structure of the complex. Similarly, the crystal structure of compound **2c** was found to contain water molecules, and then the compound **2c** was re-synthesized from MeOH/DCM solution. Single crystals of complexes **1a**, **2a**–**2c** and **2f** were mounted on glass fibers, and **1c** and **2g** were mounted in loops and fixed with Fomblin^®^ Y LVAC 25/6 oil. Intensity data were collected using a Bruker D8 Quest (for **1c**), Bruker AXS KAPPA APEX II (for **2g**) and Agilent SuperNova (for the other complexes) diffractometers with graphite monochromated Mo-Kα (λ = 0.71073 Å) radiation. The data were collected using phi and omega scans in a conventional (for **2g**) and shutterless (for the other complexes) mode using 0.5° per frame (for the other complexes), and the full sphere of data were obtained. Cell parameters were retrieved and refined by Bruker SAINT (for **1c** and **2g**) and Agilent CrysAlisPro (for the other complexes) software on all of the observed reflections [76]. Absorption corrections were applied using SADABS [77] (for **1c** and **2g**) and ABSPACK [77] (for the other complexes). The structures were solved by direct methods using the SHELX-2018/3 [78] (for **1c** and **2g**) and SHELX–97 [79] (for the other complexes) packages and refined with SHELXL–97 [79]. The thermal ellipsoid plots were drawn by Diamond 3.2 [80]. The hydrogen atoms were inserted in calculated positions theoretically. Least square refinements with anisotropic thermal motion parameters for all of the non-hydrogen atoms and isotropic for the remaining atoms were employed. CCDC 2062271–2062277 for compounds **1a**, **1c**, **2a**–**2c, 2f** and **2g** contain the supplementary crystallographic data of this paper. These data can be obtained free of charge from the Cambridge Crystallographic Data Centre via www.ccdc.cam.ac.uk/data_request/cif, accessed on 1 December 2022. Crystal data and details of data collections are reported in Table 4.

### 3.5. Antiproliferative Activity against Tumor Cells

Five different cell lines, human lung carcinoma cell line (A549), human hepatocellular carcinoma cell line (Bel-7402), human esophageal squamous carcinoma cell line (Eca-109), human cervix carcinoma cell line (HeLa) and human breast cancer cell line (MCF-7), purchased from the American Type Culture Collection (ATCC), were used to evaluate the antiproliferative activity of the synthesized compounds. All the cells were cultured with a completed Dulbecco’s Modified Eagle Medium (DMEM) supplemented with 10% fetal bovine serum, 100 U/mL penicillin and 100 U/mL streptomycin in a humidified atmosphere at 37 °C with 5% CO_2_. The cells were seeded in 96-well plates with 3000 cells per well. After 12 h, series concentrations of compounds **1a**–**1c** and **2a**–**2h** were added into the predefined wells and then incubated for 48 h. Cell morphology was observed and imaged with an inverted microscope (Nikon eclipses TS100) equipped with a Nikon digital camera (DXM 1200F). The cell viability was measured using CCK-8 assay (cell counting kit-8, Beyotime Biotechnology, China) following the manufacturer’s instructions. The cell viability (% of control) was expressed as the percentage of (OD_test_ − OD_blank_)/(OD_control_ − OD_blank_). GraphPad Prism V5.0 for Windows (Graphpad Software, San Diego, CA, USA) was used to calculate the 50% inhibitive concentration (IC_50_) of the tested compounds against tumor cells.

### 3.6. Circular Dichroism (CD) Spectropolarimetry

Circular dichroism spectra (differential absorption of left and right circularly polarized light) of CT-DNA in the presence or absence of compounds **1a**–**1c** and **2a**–**2h** at different concentrations were recorded using a Chirascan spectropolarimeter (Applied Photophysics, UK). The CD measurements were carried out using 2 mm Suprasil quartz cells from Hellma Analytics and maintained at a temperature of 20 °C using a TC125 temperature controller from Quantum Northwestern running on the Chirascan spectrophotometer. The spectra were recorded between 230 and 400 nm, with a bandwidth of 1 nm and time per point of 1 s. The spectra of 5 mM Tris-HCl and 50 mM NaCl buffer (pH 7.2) were used as the baselines, and they were automatically subtracted from the CD spectra of the samples.

### 3.7. Molecular Docking

The coordination sphere of the manganese compounds was generated from their X-ray crystal structures as CIF files. Subsequently, the CIF file was converted to the PDB format using Mercury software (http://www.ccdc.cam.ac.uk/, accessed on 1 December 2022). The X-ray crystallographic structure of B-DNA dodecamer d(CGCGAATTCGCG)2 (PDB ID: 1BNA) and human DNA–topoisomerase I complex (PDB ID: 1SC7) were retrieved and modified from the protein Data Bank (https://www.rcsb.org/, accessed on 1 December 2022). Topo I is bound to the oligonucleotide sequence 5′-AAAAAGACTTsX-GAAAATTTTT-3′, where ‘s’ is 5′-bridging phosphorothiolate of the cleaved strand, and ‘X’ represents any of the four bases A, G, C or T. The SH of G11 on the scissile strand was changed to OH, and the phosphoester bond of G12 in 1SC7 was rebuilt [81]. A molecular docking study was performed with AutoDock Tools (ADT) version 1.5.6 and AutoDock version 4.2.6 programs while using the implemented empirical free energy function and the Lamarckian Genetic Algorithm. The structures of the receptors were kept rigid during the docking, whereas the metal compound was allowed to have rotatable bonds. Prior to performing docking, all of the water molecules were charged, and polar hydrogen atoms were added. The size of the grid was set to 80 × 80 × 120 or 80 × 80 × 80 with a spacing of 0.375 Å. All of the other parameters were kept as the defaults. Among them, the conformation having the lowest energy was selected to depict the mode of interaction between the compounds and DNA. The results were visualized using the PyMol Molecular Viewer package [82,83].

## 4. Conclusions

Eight oxygen-containing substituent-modified terpyridine manganese complexes and three non-oxygen-containing substituent-modified terpyridine manganese complexes were synthesized, and their structures were characterized by IR, elemental analysis and single-crystal X-ray diffraction. The in vitro cell viability studies illustrated the anticancer potential of the compounds, with the lowest IC_50_ values of 0.281 μM (**2d**) against A549, 0.523 μM (**2h**) against Bel-7402, 0.514 μM (**2g**) against Eca-109, 0.356 μM (**2d**) against HeLa and 0.249 μM (**2c**) against MCF-7. They were more active than cisplatin against the five tested cell lines and, compound **2g** with a nitro group had the best antiproliferative activities against five cell lines on the whole. When comparing the complexes with different types of substituents, we found that complexes modified with oxygen-containing substituents showed lower IC_50_ values against all five cell lines. Then, the circular dichroism spectroscopy studies revealed a strong affinity between the compounds and the DNA in an intercalative mode. The CD spectra showed that the secondary structure of the DNA was changed by the addition of the compounds, the different substituents at the terpyridine significantly affect the interaction between the compound and the DNA, and oxygen-containing substituents seem to promote the intercalation. The molecular docking studies confirmed the interaction between the compounds and DNA or DNA–topoisomerase I complex, and the models showed that the interactions were stabilized by π–π stacking and hydrogen bonding. The results of these experiments indicated that both the steric and electrostatic effects of the compounds had strong influences on their interactions with DNA. It appears that less steric hindrance and higher electronegativity can enhance the DNA-binding affinity of the compounds. The correlation between anticancer activity and DNA-binding affinity suggests that DNA binding is a plausible mechanism. Notably, in this study and some published anticancer activity data of complexes with similar structures, the anticancer activity of the non-oxygen-containing substituent-modified terpyridine complexes was significantly lower than that of the terpyridine complexes modified with oxygen-containing substituents. Combined with the results of circular dichroism and molecular docking studies, we believe that the introduction of oxygen-containing substituents enhances the binding affinity to DNA through hydrogen bonding and π–π stacking, promotes intercalation and thus enhances their anticancer activity. Accordingly, the current results propose an effective strategy to enhance the anticancer activity of terpyridine complexes by introducing oxygen-containing substituents, which form a promising base for future in vitro and in vivo investigations of anticancer manganese metallodrugs.

## Figures and Tables

**Figure 2:**
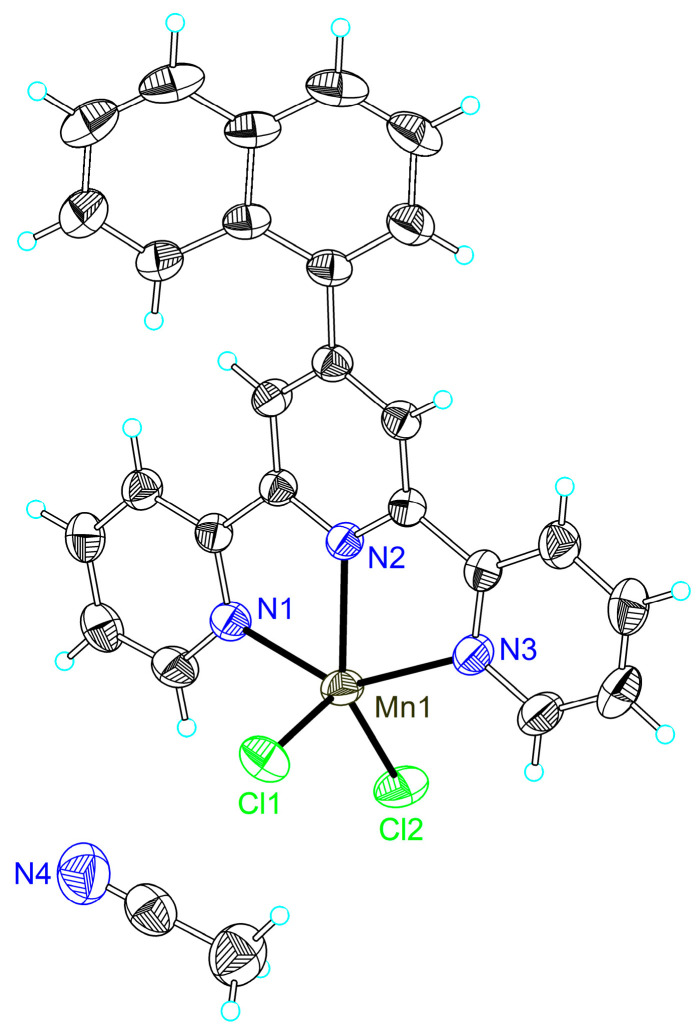
Thermal ellipsoid plot, drawn at the 50% probability level, of [MnCl_2_**L^3^**]·CH_3_CN (**3**·CH_3_CN) with atomic numbering scheme. Selected bond lengths (Å) and angles (°): Mn(1)-N(2) 2.2028(13), Mn(1)-N(1) 2.2541(15), Mn(1)-N(3) 2.2595(15), Mn(1)-Cl(1) 2.3603(6), Mn(1)-Cl(2) 2.3558(5), N(2)-Mn(1)-N(1) 71.51(5), N(2)-Mn(1)-N(3) 71.71(5), N(1)-Mn(1)-N(3) 141.23(5), N(2)-Mn(1)-Cl(1) 108.08(4), N(1)-Mn(1)-Cl(1) 103.45(4), N(3)-Mn(1)-Cl(1) 99.15(4), N(2)-Mn(1)-Cl(2) 137.27(4), N(1)-Mn(1)-Cl(2) 100.29(4), N(3)-Mn(1)-Cl(2) 98.33(4), Cl(1)-Mn(1)-Cl(2) 114.57(2).

**Table 1:** IC_50_ values (μM) from the dose-response assay of compounds **1a**–**1c** and **2a**–**2h** and the reference compound cisplatin in the A549, Bel-7402, Eca-109, HeLa and MCF-7 cell lines, after an incubation time of 48 h.

Compounds	A549		Bel-7402		Eca-109		HeLa		MCF-7	
	IC_50_ (μmol/L)	95% Confidence Intervals (μmol/L)	IC_50_ (μmol/L)	95% Confidence Intervals (μmol/L)	IC_50_ (μmol/L)	95% Confidence Intervals (μmol/L)	IC_50_ (μmol/L)	95% Confidence Intervals (μmol/L)	IC_50_ (μmol/L)	95% Confidence Intervals (μmol/L)
**1a**	2.858	2.142–3.813	2.667	0.455–0.718	3.115	2.575–3.769	7.249	6.118–8.589	1.229	0.980–1.540
**1b**	3.052	1.628–5.724	3.631	0.434–0.650	2.927	2.222–3.857	4.566	3.857–5.406	1.594	1.244–2.042
**1c**	2.811	2.034–3.883	2.751	0.308–0.450	2.196	1.818–2.652	2.935	1.765–4.883	0.850	0.671–1.077
**2a**	0.567	0.398–0.808	2.088	1.329–1.590	1.153	0.714–1.863	1.486	1.086–2.031	0.284	0.227–0.355
**2b**	0.754	0.519–1.096	0.730	0.616–0.796	0.579	0.486–0.688	1.583	1.085–2.310	0.525	0.399–0.691
**2c**	0.409	0.242–0.692	1.510	1.043–1.227	0.614	0.488–0.774	1.160	0.969–1.389	0.249	0.207–0.301
**2d**	0.281	0.217–0.364	1.382	0.304–0.471	0.645	0.502–0.829	0.356	0.280–0.452	0.819	0.583–1.151
**2e**	0.334	0.287–0.390	1.720	0.656–0.876	2.690	2.452–2.950	6.047	5.304–6.894	1.610	1.240–2.090
**2f**	0.723	0.538–0.970	1.011	0.441–0.605	0.886	0.717–1.094	2.889	1.815–4.600	0.501	0.360–0.698
**2g**	0.453	0.313–0.655	0.626	0.616–0.796	0.514	0.414–0.638	0.412	0.266–0.639	0.304	0.249–0.372
**2h**	0.626	0.455–0.862	0.523	1.043–1.227	0.868	0.677–1.111	2.648	1.683–4.167	0.379	0.317–0.454
Cisplatin	4.832	3.501–6.669	19.34	16.27–22.98	7.594	5.879–9.809	20.30	17.87–23.06	19.00	16.19–22.30

**Table 2:** The calculated free energy of binding of compounds **1a**–**1c** and **2a**–**2h** with B-DNA (1BNA), oligonucleotide (4JD8M) and DNA-Topo I complex (1SC7).

Compounds	Affinity (kcal/mol)		
	B-DNA (1BNA)	Oligonucleotide (4JD8)	DNA-Topo I Complex (1SC7)
**1a**	−9.11	−9.32	−9.97
**1b**	−10.34	−9.61	−10.23
**1c**	−10.27	−9.93	−10.89
**2a**	−9.07	−9.28	−9.58
**2b**	−9.21	−8.78	−10.26
**2c**	−9.29	−8.97	−10.39
**2d**	−9.16	−8.41	−9.97
**2e**	−8.55	−8.07	−9.75
**2f**	−10.86	−10.23	−12.34
**2g**	−9.38	−9.20	−10.53
**2h**	−8.67	−8.11	−9.06

**Table 3:** Hydrogen bond interactions for compounds **2a**–**2c**, **2f** and **2g**.

Compounds	Receptor	Bonds Formed	Bond Distance (Å)	Bond Energy (kcal/mol)
**2a**	B-DNA (1BNA)	O–H…O (DA18)	2.105	−4.619
	Oligonucleotide (4JD8)	O–H…O (DT2)	1.993	−0.824
	DNA-Topo I complex (1SC7)	O–H…O (ASN722)	2.133	−3.223
**2b**	B-DNA (1BNA)	O–H…O (DT19)	2.175	−4.074
	Oligonucleotide (4JD8)	O–H…O (DT2)	2.073	−4.052
	DNA-Topo I complex (1SC7)	O–H…O (ASN722)	2.081	−0.401
	DNA-Topo I complex (1SC7)	O…H–N (ASN722)	1.956	−3.016
**2c**	B-DNA (1BNA)	O–H…O (DT7)	1.804	−1.485
	Oligonucleotide (4JD8)	O–H…O (DG3)	2.231	−5.235
	DNA-Topo I complex (1SC7)	O–H…O (TGP11)	2.134	−6.073
**2f**	B-DNA (1BNA)	O…H–N(DG16)	2.061	−5.108
	Oligonucleotide (4JD8)	O…H–N(DA5)	2.130	−4.107
	DNA-Topo I complex (1SC7)	O…H–N (MET428)	1.990	−4.015
	DNA-Topo I complex (1SC7)	O…H–N (ALA351)	2.204	−1.600
**2g**	B-DNA (1BNA)	O…H–N (DG14)	2.079	−1.999
	Oligonucleotide (4JD8)	O…H–N(DA5)	2.201	−2.900
	DNA-Topo I complex (1SC7)	O…H–N (LYS425)	1.877	−4.568

**Table 4:** Hydrogen bond interactions for compounds **1a**, **1c**, **2a**–**2c**, **2f** and **2g**.

Compound	1a	1c	2a	2b	2c	2f	2g
Empirical formula	C_21_H_15_Cl_2_MnN_3_	C_27_H_20_Cl_2_MnN_4_	C_21_H_15_Cl_2_MnN_3_O	C_21_H_15_Cl_2_MnN_3_O	C_21_H_19_Cl_2_MnN_3_O	C_22_H_17_Cl_2_MnN_3_O_2_S	C_21_H_14_N_4_O_2_Cl_2_Mn
Formula weight	435.20	526.31	451.2	451.2	455.23	513.29	480.2
Temperature	123(2) K	293(2) K	293(2) K	293(2) K	293(2) K	293(2) K	150(2) K
Crystal system	Monoclinic	Triclinic	Monoclinic	Monoclinic	Triclinic	Monoclinic	Monoclinic
Space group	P2_1_/*n*	P-1	P2_1_/*n*	P2_1_/*n*	P-1	P2_1_/*n*	C2/*c*
*a* (Å)	12.1515(5)	8.6474(6)	12.0365(11)	12.2520(15)	8.9693(18)	10.2754(15)	11.1314(16)
*b* (Å)	9.7548(4)	11.0270(7)	9.8576(10)	9.7098(10)	10.584(2)	18.416(2)	22.013(3)
*c* (Å)	16.6109(6)	13.3718(10)	17.1166(17)	17.083(2)	12.780(3)	12.7472(17)	8.6607(11)
*α* (°)	90.00	85.629(3)	90	90	103.63(3)	90	90
*β* (°)	106.581(2)	74.006(3)	107.487(11)	107.349(14)	108.46(3)	110.752(16)	110.471(8)
*γ* (°)	90.00	87.326(3)	90	90	96.63(3)	90	90
Volume (Å^3^)	1887.10(13)	1221.70(15)	1937.1(3)	1939.8(4)	1094.4(4)	2255.7(5)	1988.1(5)
*Z*	4	2	4	4	2	4	4
Calculated density (Mg/m^3^)	1.532	1.431	1.547	1.545	1.382	1.511	1.604
Absorption coefficient (mm^−1^)	0.993	0.782	0.974	0.973	0.863	0.939	0.96
*F*(000)	884	538	916	916	466	1044	972
Crystal size (mm^−1^)	0.41 × 0.33 × 0.29	0.40 × 0.36 × 0.36	0.48 × 0.33 × 0.31	0.48 × 0.46 × 0.26	0.41 × 0.23 × 0.16	0.45 × 0.40 × 0.38	0.26 × 0.22 × 0.15
*θ*_max_, *θ*_min_ (°)	30.69, 1.85	26.45, 2.36	29.40, 3.24	29.47, 3.21	29.27, 2.84	29.54, 2.80	30.07, 2.75
Index range *h*	–17 → 17	–10 → 10	–16 → 14	–15 → 14	–8 → 12	–13 → 13	–11 → 15
*K*	–13 → 13	–13 → 13	–13 → 12	–13 → 10	–14 → 13	–25 → 17	–27 → 30
*L*	–23 → 23	–16 → 16	–23 → 22	–16 → 23	–17 → 16	–16 → 17	–11 → 12
Reflections collected /unique	36,786/5834 [*R*(int) = 0.0378]	41,099/5022 [*R*(int) = 0.0555]	10,821/4560 [*R*(int) = 0.0214]	11,912/4640 [*R*(int) = 0.0228]	9493/5049 [*R*(int) = 0.0164]	12,816/5327 [*R*(int) = 0.0248]	10,177/2910 [*R*(int) = 0.0558]
Data/restraints/parameters	5834/0/244	5022/0/376	4560/0/254	4640/0/253	5049/0/287	5327/0/280	2910/0/177
GOF on *F*^2^	1.034	1.003	1.031	1.002	1.034	1.030	1.031
Final *R* indices [*I*>2σ(*I*)]	*R*_1_ = 0.0248 *wR*_2_ = 0.0683	*R*_1_ = 0.0304 *wR*_2_ = 0.0804	*R*_1_ = 0.0314 *wR*_2_ = 0.0777	*R*_1_ = 0.0320 *wR*_2_ = 0.0801	*R*_1_ = 0.0368 *wR*_2_ = 0.0949	*R*_1_ = 0.0364 *wR*_2_ = 0.0957	*R*_1_ = 0.0492 *wR*_2_ = 0.1123
*R* indices (all data)	*R*_1_ = 0.0283 *wR*_2_ = 0.0704	*R*_1_ = 0.0424 *wR*_2_ = 0.0899	*R*_1_ = 0.0423 *wR*_2_ = 0.0866	*R*_1_ = 0.0457 *wR*_2_ = 0.0910	*R*_1_ = 0.0476 *wR*_2_ = 0.1046	*R*_1_ = 0.0540 *wR*_2_ = 0.1078	*R*_1_ = 0.0862 *wR*_2_ = 0.1296
Largest diff. peak and hole (e Å^–3^)	0.41 and −0.25	0.19 and −0.32	0.26 and −0.27	0.20 and −0.29	0.38 and −0.41	0.79 and −0.34	0.93 and −0.64
CCDC number	2062271	2062273	2062274	2062275	2062276	2062277	2062272

## Data Availability

Crystallographic data for the structures reported in this manuscript have been deposited with the Cambridge Crystallographic Data Centre under the CCDC numbers: 2062271 (Compound **1a**), 2062273 (Compound **1c**), 2062274 (Compound **2a**), 2062275 (Compound **2b**), 2062276 (Compound **2c**), 2062277 (Compound **2f**) and 2062272 (Compound **2g**). Copies of these data can be obtained free of charge from https://www.ccdc.cam.ac.uk/structures/, accessed on 1 December 2022.

## Supplementary Materials

The following supporting information can be downloaded at: https://www.mdpi.com/article/10.3390/ijms24043903/s1.
