# Supplementary material for: A New Concept of Enhancing the Anticancer Activity of Manganese Terpyridine Complex by Oxygen-Containing Substituent Modification
Source: Int J Mol Sci. 2023 Feb 15;24(4):3903. doi: 10.3390/ijms24043903 (PMC9963696; doi:10.3390/ijms24043903)
Supplement: Supplementary file 1 [file ijms-24-03903-s001.zip › ijms-2191296-supplementary.pdf]

Supporting Information for

A new concept of enhancing anticancer activity of terpyridine complex by oxygen-containing substituents modification

Jiahe Li, [1,2,3] Min Chen,[1] Jinzhang Jiang,[1] Jieyou Huang,[1] Hailan Chen,[4] Lixia Pan,[2] Dmytro S. Nesterov,[3] Zhen Ma, [1,3] and Armando J. L. Pombeiro[3,5]

[1] School of Chemistry and Chemical Engineering, Guangxi University, Nanning 530004, People's Republic of China.
[2] National Engineering Research Center for Non-Food Biorefinery, State Key Laboratory of Non-Food Biomass and Enzyme Technology, Guangxi Academy of Sciences, Nanning 530007, People’s Republic of China.
[3] Centro de Química Estrutural, Institute of Molecular Sciences, Instituto Superior Técnico, Universidade de Lisboa, Lisbon 1049-001, Portugal.
[4] School of Animal Science and Technology, Guangxi University, Nanning 530004, People's Republic of China.
[5] Research Institute of Chemistry, Peoples’ Friendship University of Russia (RUDN University), Moscow 117198, Russian Federation.

INDEX

Figure S1 The IR spectrum of complex **1a**. S7

Figure S2 The IR spectrum of complex **1b**. S7

Figure S3 The IR spectrum of complex **1c**. S8

Figure S4 The IR spectrum of complex **2a**. S8

Figure S5 The IR spectrum of complex **2b**. S9

Figure S6 The IR spectrum of complex **2c**. S9

Figure S7 The IR spectrum of complex **2d**. S10

Figure S8 The IR spectrum of complex **2e**. S10

Figure S9 The IR spectrum of complex **2f**. S11

Figure S10 The IR spectrum of complex **2g**. S11

Figure S11 The IR spectrum of complex **2e**. S12

Figure S12 Thermal ellipsoid plot, drawn at the 50% probability level, of [$MnCl_2$**L**^{1a}] (**1a**) with atomic numbering scheme. S12

Figure S13 The packing diagram of [$MnCl_2$**L**^{1a}] (**1a**). S13

Figure S14 The packing diagram of [$MnCl_2$**L**^{1c}]·CH_3CN (**1c**·CH_3CN). S13

Figure S15 Thermal ellipsoid plot, drawn at the 50% probability level, of [$MnCl_2$**L**^{2a}] (**2a**) with atomic numbering scheme. S14

Figure S16 The packing diagram of [$MnCl_2$**L**^{2a}] (**2a**). S14

Figure S17 Thermal ellipsoid plot, drawn at the 50% probability level, of [$MnCl_2$**L**^{2b}] (**2b**) with atomic numbering scheme. S15

Figure S18 The packing diagram of [$MnCl_2$**L**^{2b}] (**2b**). S15

Figure S19 Thermal ellipsoid plot, drawn at the 50% probability level, of [$MnCl_2$**L**^{2c}]·$2H_2O$ (**2c**·$2H_2O$) with atomic numbering scheme. S16

Figure S20 The packing diagram of [$MnCl_2$**L**^{2c}]·$2H_2O$ (**2c**·$2H_2O$). S16

Figure S21 Thermal ellipsoid plot, drawn at the 50% probability level, of [$MnCl_2$**L**^{2f}] (**2f**) with atomic numbering scheme. S17

Figure S22 The packing diagram of [$MnCl_2$**L**^{2f}] (**2f**). S17

Figure S23 Thermal ellipsoid plot, drawn at the 50% probability level, of [$MnCl_2$**L**^{2g}]

(**2g**) with atomic numbering scheme..S18
Figure S24 The packing diagram of [$MnCl_2$**L**^{2g}] (**2g**). S18
Figure S25 The microscopic images of A549 cells treated with increased concentrations of the compounds **1b**, **1c** and **2a**–**2c**...S35
Figure S26 The microscopic images of A549 cells treated with increased concentrations of the compounds **2d**–**2h** and cisplatin..S36
Figure S27 The microscopic images of Bel-7402 cells treated with increased concentrations of the compounds **1b**, **1c** and **2a**–**2c**...S37
Figure S28 The microscopic images of A549 cells treated with increased concentrations of the compounds **2d**–**2h** and cisplatin..S38
Figure S29 The microscopic images of Eca-109 cells treated with increased concentrations of the compounds **1b**, **1c** and **2a**–**2c**...S39
Figure S30 The microscopic images of Eca-109 cells treated with increased concentrations of the compounds **2d**–**2h** and cisplatin..S40
Figure S31 The microscopic images of HeLa cells treated with increased concentrations of the compounds **1b**, **1c** and **2a**–**2c**...S41
Figure S32 The microscopic images of HeLa cells treated with increased concentrations of the compounds **2d**–**2h** and cisplatin..S42
Figure S33 The microscopic images of MCF-7 cells treated with increased concentrations of the compounds **1b**, **1c** and **2a**–**2c**...S43
Figure S34 The microscopic images of MCF-7 cells treated with increased concentrations of the compounds **2d**–**2h** and cisplatin..S44
Figure S35 The plots of cell viability *vs.* the concentration of compounds **1a–1c** and **2a–2h** against A549 cells...S45
Figure S36 The plots of cell viability *vs.* the concentration of compounds **1a–1c** and **2a–2h** against Bel-7402 cells..S45
Figure S37 The plots of cell viability *vs.* the concentration of compounds **1a–1c** and **2a–2h** against HeLa cells...S46
Figure S38 The plots of cell viability *vs.* the concentration of compounds **1a–1c** and **2a–2h** against MCF-7 cells..S46

Figure S39 Circular dichroism spectra of CT-DNA (6.0×10^{-4} mol/L) in the presence or absence of compounds **1a** in Tris-HCl buffer (pH 7.2), at 20 °C. S47
Figure S40 Circular dichroism spectra of CT-DNA (6.0×10^{-4} mol/L) in the presence or absence of compounds **1b** in Tris-HCl buffer (pH 7.2), at 20 °C. S47
Figure S41 Circular dichroism spectra of CT-DNA (6.0×10^{-4} mol/L) in the presence or absence of compounds **2a** in Tris-HCl buffer (pH 7.2), at 20 °C. S48
Figure S42 Circular dichroism spectra of CT-DNA (6.0×10^{-4} mol/L) in the presence or absence of compounds **2b** in Tris-HCl buffer (pH 7.2), at 20 °C. S48
Figure S43 Circular dichroism spectra of CT-DNA (6.0×10^{-4} mol/L) in the presence or absence of compounds **2d** in Tris-HCl buffer (pH 7.2), at 20 °C. S49
Figure S44 Circular dichroism spectra of CT-DNA (6.0×10^{-4} mol/L) in the presence or absence of compounds **2e** in Tris-HCl buffer (pH 7.2), at 20 °C..............................S49
Figure S45 Circular dichroism spectra of CT-DNA (6.0×10^{-4} mol/L) in the presence or absence of compounds **2f** in Tris-HCl buffer (pH 7.2), at 20 °C. S50
Figure S46 Circular dichroism spectra of CT-DNA (6.0×10^{-4} mol/L) in the presence or absence of compounds **2g** in Tris-HCl buffer (pH 7.2), at 20 °C. S50
Figure S47 Circular dichroism spectra of CT-DNA (6.0×10^{-4} mol/L) in the presence or absence of compounds **2h** in Tris-HCl buffer (pH 7.2), at 20 °C. S51
Figure S48 The most favorable orientation of compound **1a** with the minor groove of the B-DNA (PDB ID: 1BNA). S54
Figure S49 The most favorable orientation of compound **1b** with the minor groove of the B-DNA (PDB ID: 1BNA). S54
Figure S50 The most favorable orientation of compound **1c** with the minor groove of the B-DNA (PDB ID: 1BNA). S54
Figure S51 The most favorable orientation of compound **2b** with the minor groove of the B-DNA (PDB ID: 1BNA). The formed hydrogen bond and distance have been marked...S55
Figure S52 The most favorable orientation of compound **2c** with the minor groove of the B-DNA (PDB ID: 1BNA). The formed hydrogen bond and distance have been marked...S55

Figure S53 The most favorable orientation of compound **2d** with the minor groove of the B-DNA (PDB ID: 1BNA). S55
Figure S54 The most favorable orientation of compound **2e** with the minor groove of the B-DNA (PDB ID: 1BNA). The formed hydrogen bond and distance have been marked..S56
Figure S55 The most favorable orientation of compound **2f** with the minor groove of the B-DNA (PDB ID: 1BNA). The formed hydrogen bond and distance have been marked..S56
Figure S56 The most favorable orientation of compound **2g** with the minor groove of the B-DNA (PDB ID: 1BNA). The formed hydrogen bond and distance have been marked..S56
Figure S57 The most favorable orientation of compound **2h** with the minor groove of the B-DNA (PDB ID: 1BNA). S57
Figure S58 The most favorable orientation of compound **1a** intercalating with the DNA (4JD8). S57
Figure S59 The most favorable orientation of compound **1b** intercalating with the DNA (4JD8). S57
Figure S60 The most favorable orientation of compound **1c** intercalating with the DNA (4JD8). S58
Figure S61 The most favorable orientation of compound **2b** intercalating with the DNA (4JD8). The formed hydrogen bond and distance have been marked.......................S58
Figure S62 The most favorable orientation of compound **2c** intercalating with the DNA (4JD8). The formed hydrogen bond and distance have been marked.......................S58
Figure S63 The most favorable orientation of compound **2d** intercalating with the DNA (4JD8). S59
Figure S64 The most favorable orientation of compound **2e** intercalating with the DNA (4JD8). S59
Figure S65 The most favorable orientation of compound **2f** intercalating with the DNA (4JD8). S59
Figure S66 The most favorable orientation of compound **2g** intercalating with the DNA

(4JD8). S60

Figure S67 The most favorable orientation of compound **2h** intercalating with the DNA (4JD8). S60

Figure S68 Molecular docking models of compound **1a** in the active site of DNA–Topo I complex (PDB ID: 1SC7). S60

Figure S69 Molecular docking models of compound **1b** in the active site of DNA–Topo I complex (PDB ID: 1SC7). S61

Figure S70 Molecular docking models of compound **1c** in the active site of DNA–Topo I complex (PDB ID: 1SC7). S61

Figure S71 Molecular docking models of compound **2b** in the active site of DNA–Topo I complex (PDB ID: 1SC7). The formed hydrogen bond and distance have been marked. S61

Figure S72 Molecular docking models of compound **2c** in the active site of DNA–Topo I complex (PDB ID: 1SC7). The formed hydrogen bond and distance have been marked. S62

Figure S73 Molecular docking models of compound **2d** in the active site of DNA–Topo I complex (PDB ID: 1SC7). The formed hydrogen bond and distance have been marked. S62

Figure S74 Molecular docking models of compound **2e** in the active site of DNA–Topo I complex (PDB ID: 1SC7). The formed hydrogen bond and distance have been marked. S62

Figure S75 Molecular docking models of compound **2f** in the active site of DNA–Topo I complex (PDB ID: 1SC7). S63

Figure S76 Molecular docking models of compound **2g** in the active site of DNA–Topo I complex (PDB ID: 1SC7). The formed hydrogen bond and distance have been marked. S63

Figure S77 Molecular docking models of compound **2h** in the active site of DNA–Topo I complex (PDB ID: 1SC7). S63

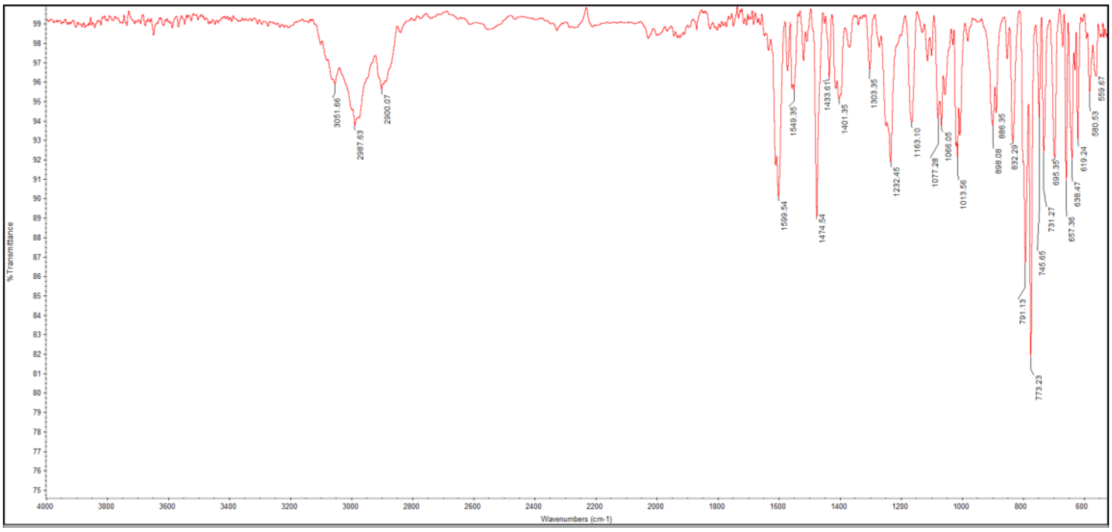

Figure S1 The IR spectrum of complex **1a**.

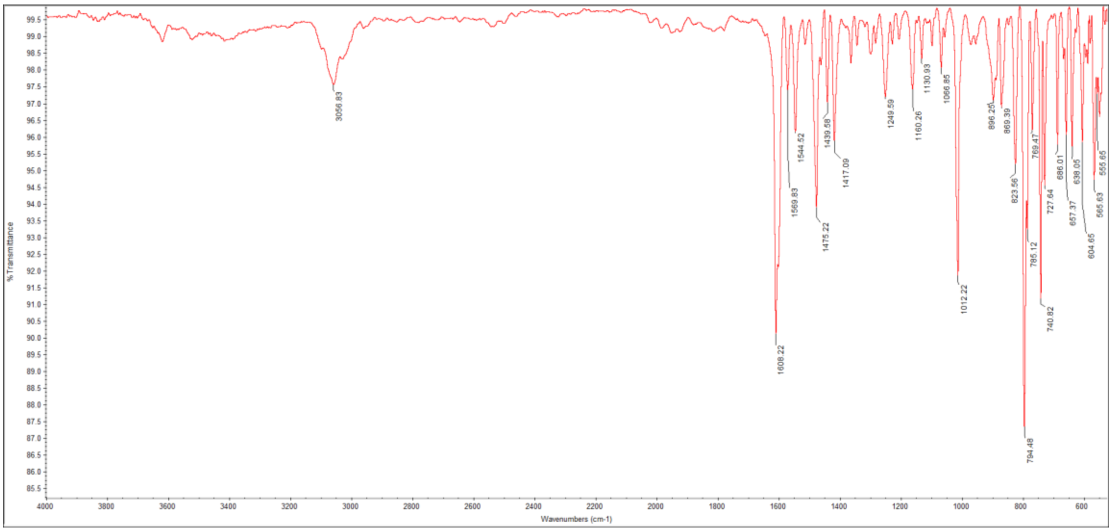

Figure S2 The IR spectrum of complex **1b**.

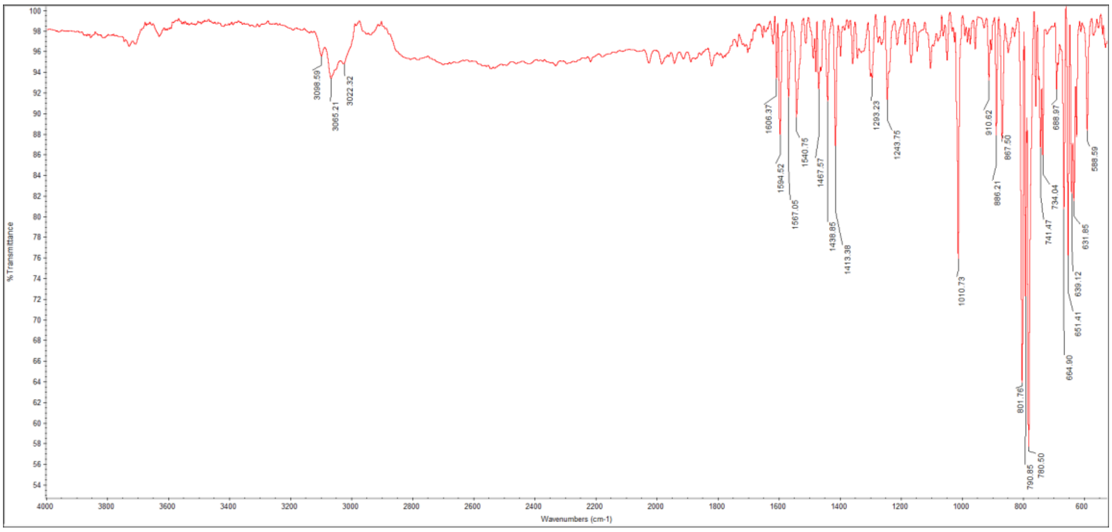

Figure S3 The IR spectrum of complex **1c**.

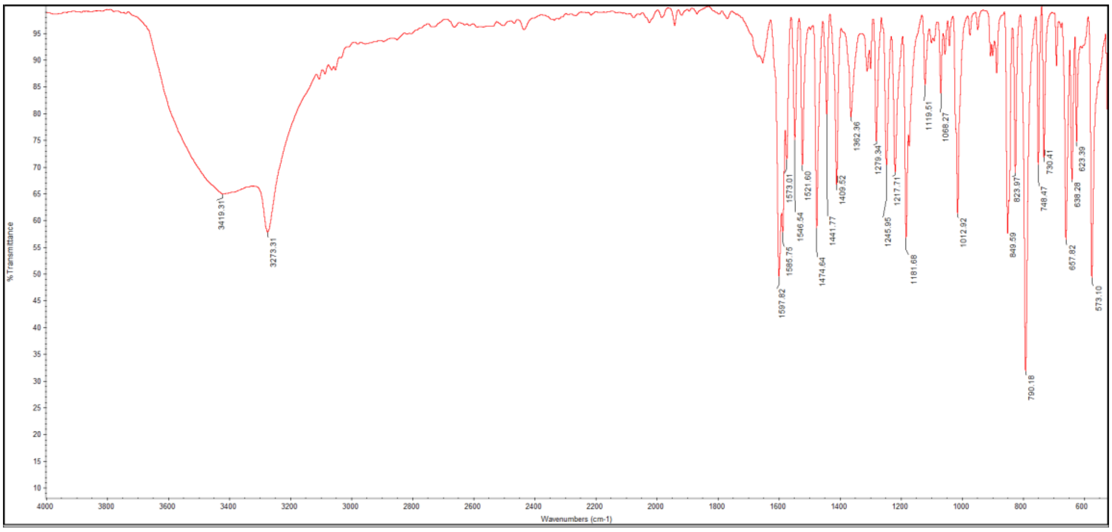

Figure S4 The IR spectrum of complex **2a**.

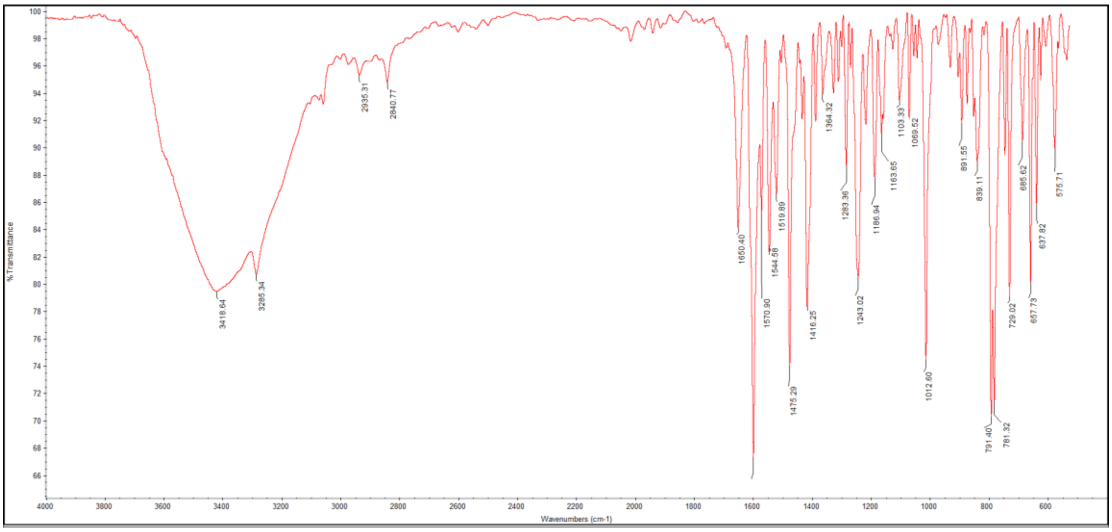

Figure S5 The IR spectrum of complex **2b**.

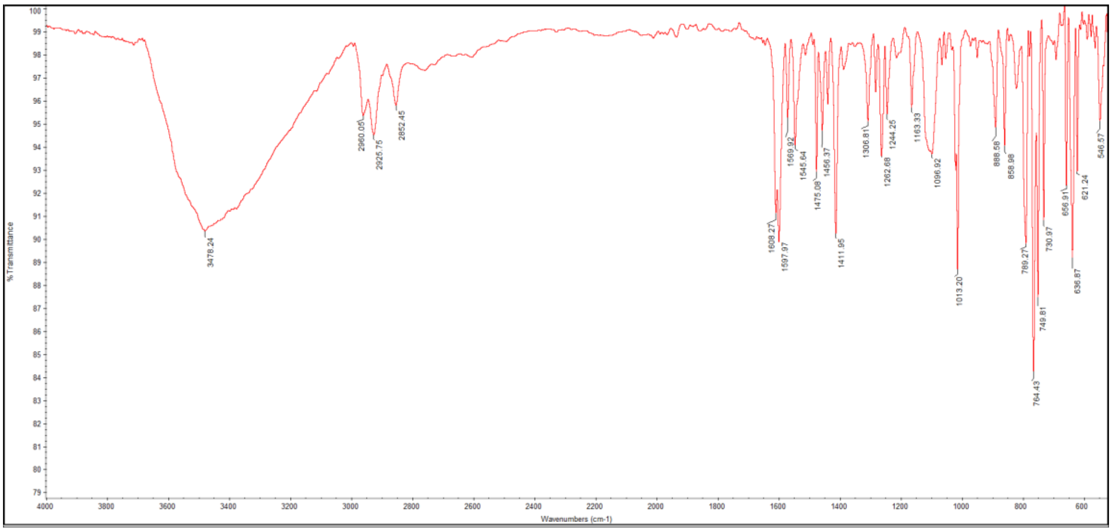

Figure S6 The IR spectrum of complex **2c**.

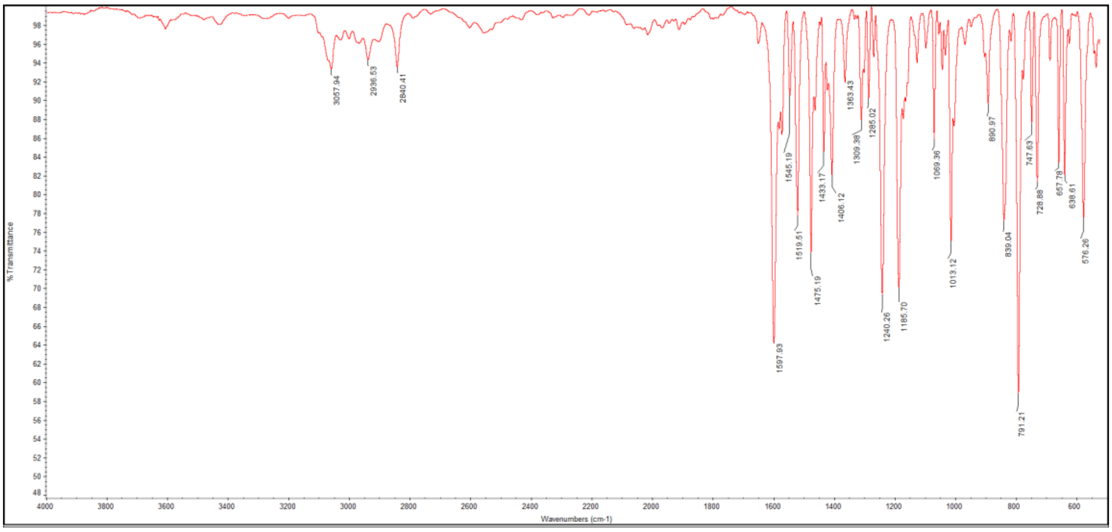

Figure S7 The IR spectrum of complex **2d**.

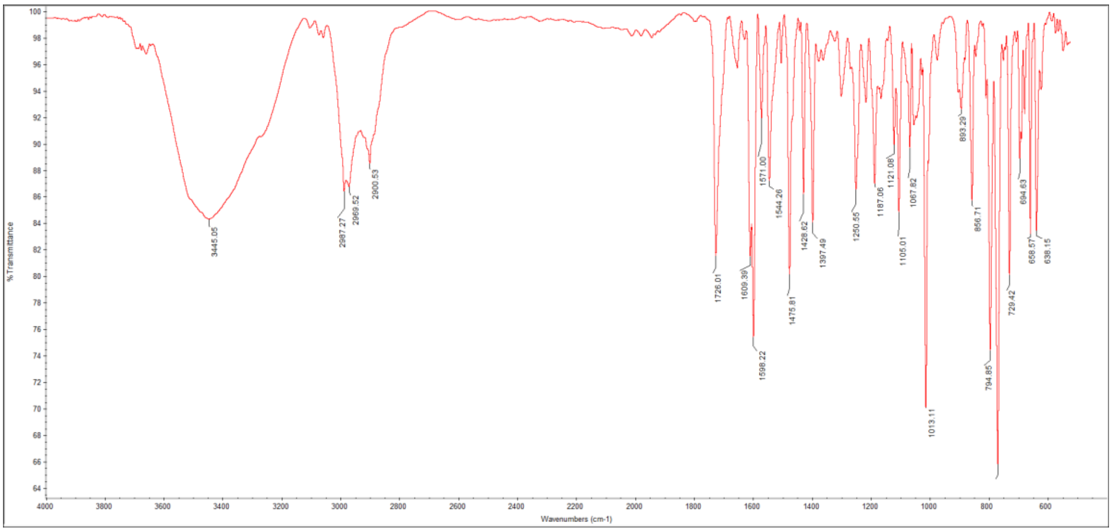

Figure S8 The IR spectrum of complex **2e**.

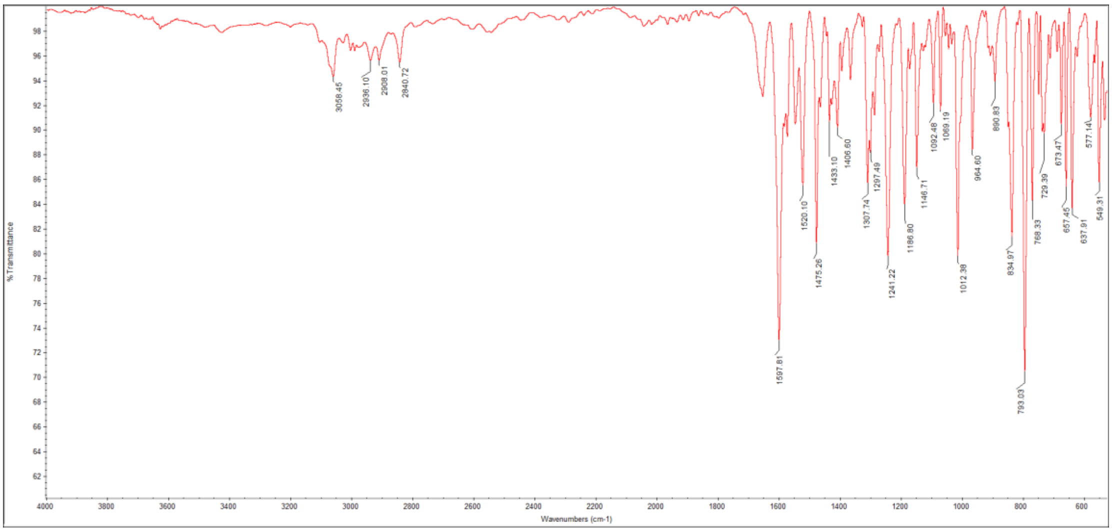

Figure S9 The IR spectrum of complex **2f**.

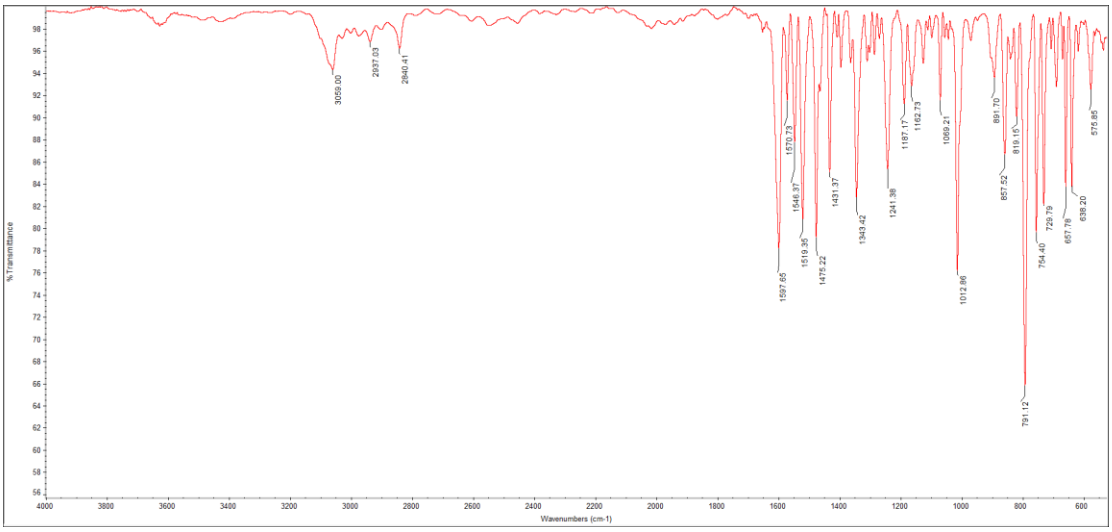

Figure S10 The IR spectrum of complex **2g**.

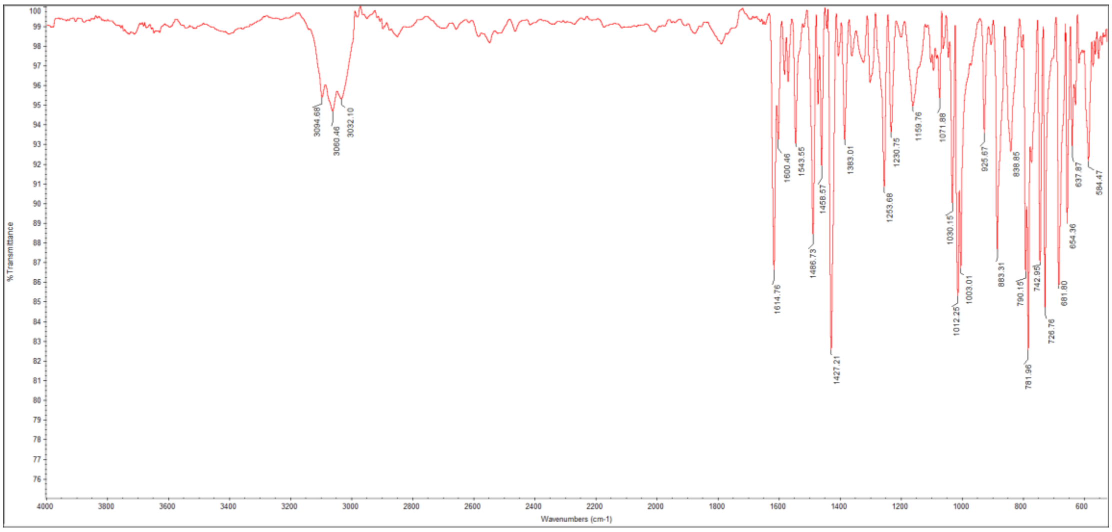

Figure S11 The IR spectrum of complex **2e**.

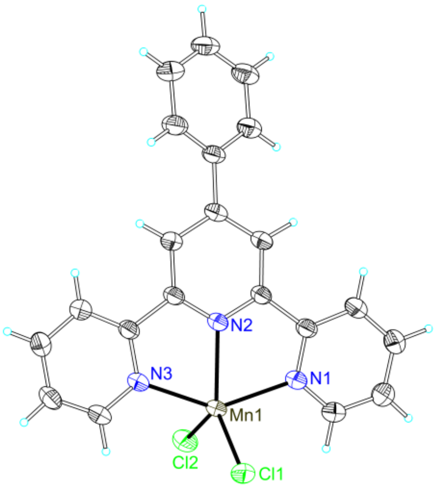

Figure S12 Thermal ellipsoid plot, drawn at the 50% probability level, of [$MnCl_2$**L**[**1a**]] (**1a**) with atomic numbering scheme.

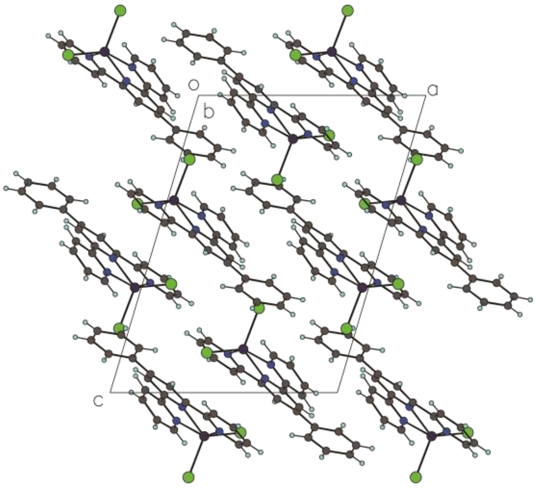

Figure S13 The packing diagram of [$MnCl_2$**L**^{1a}] (**1a**).

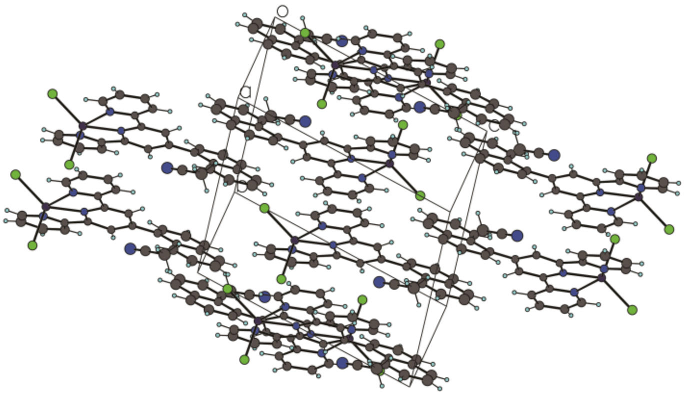

Figure S14 The packing diagram of [$MnCl_2$**L**^{1c}]·CH_3CN (**1c**·CH_3CN).

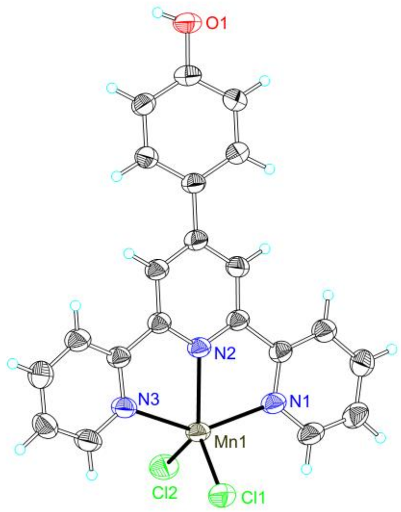

Figure S15 Thermal ellipsoid plot, drawn at the 50% probability level, of [$MnCl_2$**L**$^{\mathbf{2a}}$] (**2a**) with atomic numbering scheme.

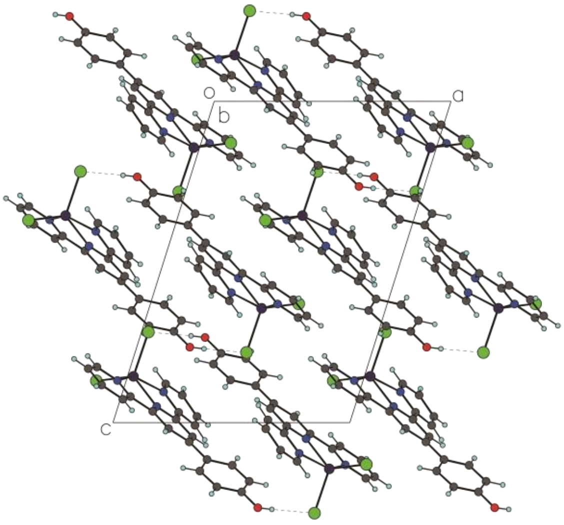

Figure S16 The packing diagram of [$MnCl_2$**L**$^{\mathbf{2a}}$] (**2a**).

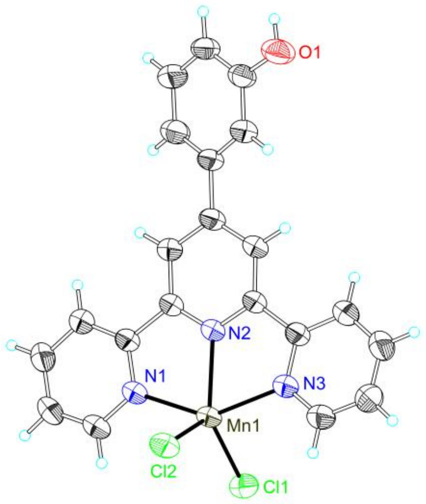

Figure S17 Thermal ellipsoid plot, drawn at the 50% probability level, of [$MnCl_2$**L**$^{\mathbf{2b}}$] (**2b**) with atomic numbering scheme.

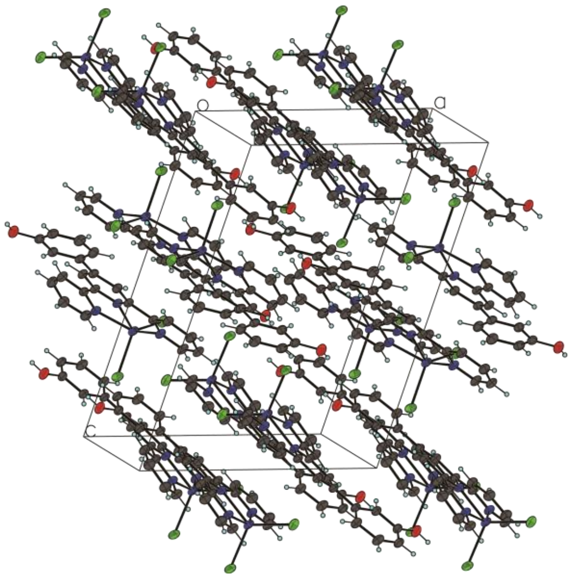

Figure S18 The packing diagram of [$MnCl_2$**L**$^{\mathbf{2b}}$] (**2b**).

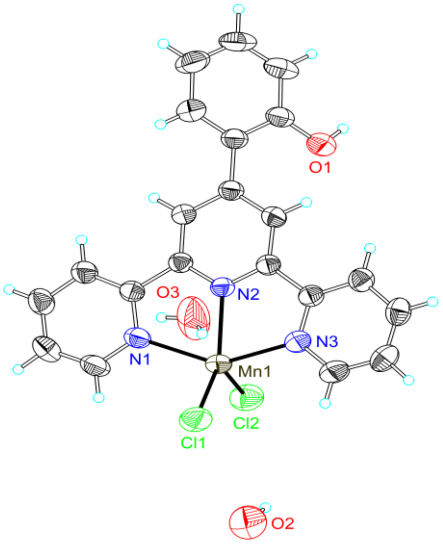

Figure S19 Thermal ellipsoid plot, drawn at the 50% probability level, of [$MnCl_2$**L**^{2c}]·$2H_2O$ (**2c**·$2H_2O$) with atomic numbering scheme.

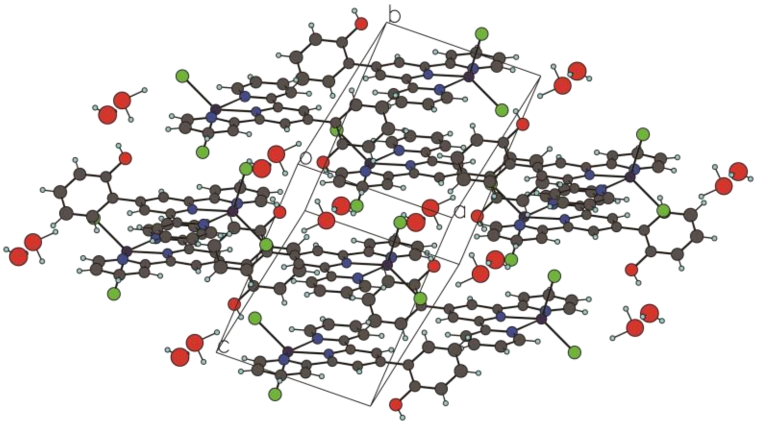

Figure S20 The packing diagram of [$MnCl_2$**L**^{2c}]·$2H_2O$ (**2c**·$2H_2O$).

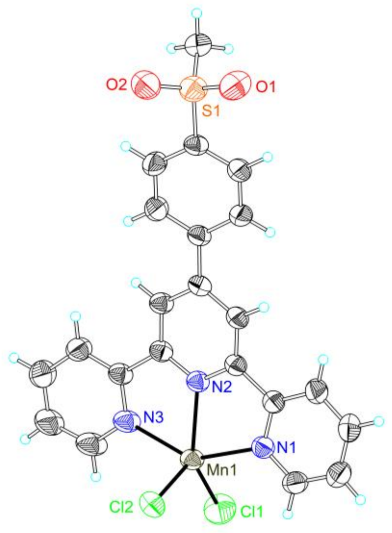

Figure S21 Thermal ellipsoid plot, drawn at the 50% probability level, of [$MnCl_2$**L**^{2f}] (**2f**) with atomic numbering scheme.

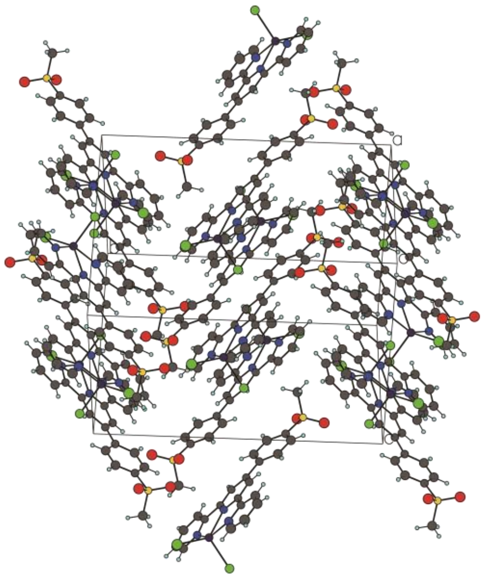

Figure S22 The packing diagram of [$MnCl_2$**L**^{2f}] (**2f**).

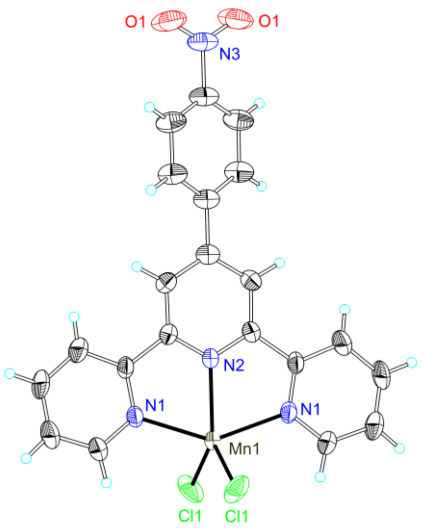

Figure S23 Thermal ellipsoid plot, drawn at the 50% probability level, of [$MnCl_2$**L**$^{\mathbf{2g}}$] (**2g**) with atomic numbering scheme.

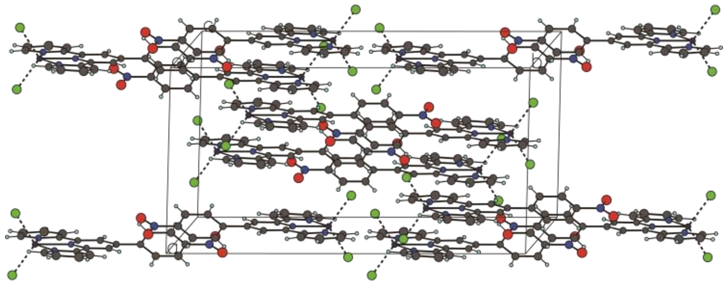

Figure S24 The packing diagram of [$MnCl_2$**L**$^{\mathbf{2g}}$] (**2g**).

Table S1 Selected bond lengths of complex **1a**

	Bond length (Å)		Bond length (Å)		Bond length (Å)
Mn(1)-N(2)	2.2079(9)	C(3)-H(3A)	0.95	C(13)-H(13A)	0.95
Mn(1)-N(1)	2.2508(10)	C(4)-C(5)	1.3917(16)	C(14)-C(15)	1.3836(19)
Mn(1)-N(3)	2.2621(11)	C(4)-H(4A)	0.95	C(14)-H(14A)	0.95
Mn(1)-Cl(3)	2.3595(3)	C(5)-C(6)	1.4839(16)	C(15)-H(15A)	0.95
Mn(1)-Cl(2)	2.3614(4)	C(6)-C(7)	1.3892(15)	C(16)-C(17)	1.4003(17)
N(1)-C(1)	1.3358(16)	C(7)-C(8)	1.3965(17)	C(16)-C(21)	1.4006(16)
N(1)-C(5)	1.3489(14)	C(7)-H(7A)	0.95	C(17)-C(18)	1.3943(17)
N(2)-C(10)	1.3410(15)	C(8)-C(9)	1.4003(15)	C(17)-H(17A)	0.95
N(2)-C(6)	1.3435(14)	C(8)-C(16)	1.4839(15)	C(18)-C(19)	1.385(2)
N(3)-C(15)	1.3414(14)	C(9)-C(10)	1.3914(15)	C(18)-H(18A)	0.95
N(3)-C(11)	1.3521(14)	C(9)-H(9A)	0.95	C(19)-C(20)	1.387(2)
C(1)-C(2)	1.3849(17)	C(10)-C(11)	1.4870(15)	C(19)-H(19A)	0.95
C(1)-H(1A)	0.95	C(11)-C(12)	1.3892(17)	C(20)-C(21)	1.3885(16)
C(2)-C(3)	1.3851(17)	C(12)-C(13)	1.3906(17)	C(20)-H(20A)	0.95
C(2)-H(2A)	0.95	C(12)-H(12A)	0.95	C(21)-H(21A)	0.95
C(3)-C(4)	1.3894(18)	C(13)-C(14)	1.3870(18)		

Table S2 Selected bond angles of complex **1a**

	Bond angles (°)		Bond angles (°)
N(2)-Mn(1)-N(1)	71.86(4)	C(7)-C(8)-C(16)	121.39(10)
N(2)-Mn(1)-N(3)	71.80(4)	C(9)-C(8)-C(16)	120.95(11)
N(1)-Mn(1)-N(3)	143.56(4)	C(10)-C(9)-C(8)	119.59(11)
N(2)-Mn(1)-Cl(3)	129.54(3)	C(10)-C(9)-H(9A)	120.2
N(1)-Mn(1)-Cl(3)	98.38(3)	C(8)-C(9)-H(9A)	120.2
N(3)-Mn(1)-Cl(3)	102.57(3)	N(2)-C(10)-C(9)	121.77(10)
N(2)-Mn(1)-Cl(2)	115.49(3)	N(2)-C(10)-C(11)	114.67(9)
N(1)-Mn(1)-Cl(2)	99.85(3)	C(9)-C(10)-C(11)	123.55(10)

N(3)-Mn(1)-Cl(2)	97.87(3)	N(3)-C(11)-C(12)	121.89(10)
Cl(3)-Mn(1)-Cl(2)	114.948(13)	N(3)-C(11)-C(10)	115.14(10)
C(1)-N(1)-C(5)	119.01(10)	C(12)-C(11)-C(10)	122.97(10)
C(1)-N(1)-Mn(1)	122.94(8)	C(11)-C(12)-C(13)	119.17(11)
C(5)-N(1)-Mn(1)	118.05(8)	C(11)-C(12)-H(12A)	120.4
C(10)-N(2)-C(6)	119.45(10)	C(13)-C(12)-H(12A)	120.4
C(10)-N(2)-Mn(1)	120.05(7)	C(14)-C(13)-C(12)	118.98(12)
C(6)-N(2)-Mn(1)	120.17(8)	C(14)-C(13)-H(13A)	120.5
C(15)-N(3)-C(11)	118.24(11)	C(12)-C(13)-H(13A)	120.5
C(15)-N(3)-Mn(1)	124.04(8)	C(15)-C(14)-C(13)	118.49(11)
C(11)-N(3)-Mn(1)	117.53(7)	C(15)-C(14)-H(14A)	120.8
N(1)-C(1)-C(2)	122.67(11)	C(13)-C(14)-H(14A)	120.8
N(1)-C(1)-H(1A)	118.7	N(3)-C(15)-C(14)	123.23(11)
C(2)-C(1)-H(1A)	118.7	N(3)-C(15)-H(15A)	118.4
C(1)-C(2)-C(3)	118.41(12)	C(14)-C(15)-H(15A)	118.4
C(1)-C(2)-H(2A)	120.8	C(17)-C(16)-C(21)	118.53(11)
C(3)-C(2)-H(2A)	120.8	C(17)-C(16)-C(8)	120.93(11)
C(2)-C(3)-C(4)	119.46(11)	C(21)-C(16)-C(8)	120.49(11)
C(2)-C(3)-H(3A)	120.3	C(18)-C(17)-C(16)	120.53(12)
C(4)-C(3)-H(3A)	120.3	C(18)-C(17)-H(17A)	119.7
C(3)-C(4)-C(5)	118.64(11)	C(16)-C(17)-H(17A)	119.7
C(3)-C(4)-H(4A)	120.7	C(19)-C(18)-C(17)	120.14(13)
C(5)-C(4)-H(4A)	120.7	C(19)-C(18)-H(18A)	119.9
N(1)-C(5)-C(4)	121.65(11)	C(17)-C(18)-H(18A)	119.9
N(1)-C(5)-C(6)	115.07(10)	C(18)-C(19)-C(20)	119.84(11)
C(4)-C(5)-C(6)	123.25(10)	C(18)-C(19)-H(19A)	120.1
N(2)-C(6)-C(7)	121.81(11)	C(20)-C(19)-H(19A)	120.1
N(2)-C(6)-C(5)	114.48(9)	C(19)-C(20)-C(21)	120.36(12)
C(7)-C(6)-C(5)	123.65(10)	C(19)-C(20)-H(20A)	119.8

C(6)-C(7)-C(8)	119.69(10)	C(21)-C(20)-H(20A)	119.8
C(6)-C(7)-H(7A)	120.2	C(20)-C(21)-C(16)	120.54(12)
C(8)-C(7)-H(7A)	120.2	C(20)-C(21)-H(21A)	119.7
C(7)-C(8)-C(9)	117.62(10)	C(16)-C(21)-H(21A)	119.7

Table S3 Selected bond lengths of complex **1c**

	Bond length (Å)		Bond length (Å)		Bond length (Å)
Mn(1)-Cl(1)	2.3603(6)	C(6)-C(7)	1.385(2)	C(21)-C(22)	1.413(3)
Mn(1)-Cl(2)	2.3558(5)	C(10)-C(11)	1.487(2)	C(9)-H(9)	0.93
Mn(1)-N(2)	2.2028(13)	C(10)-C(9)	1.384(2)	C(3)-H(3)	0.93
Mn(1)-N(1)	2.2541(15)	C(7)-H(7)	0.93	C(3)-C(2)	1.371(3)
Mn(1)-N(3)	2.2595(15)	C(7)-C(8)	1.398(2)	C(2)-H(2)	0.93
N(2)-C(6)	1.341(2)	C(16)-C(8)	1.488(2)	C(2)-C(1)	1.372(3)
N(2)-C(10)	1.340(2)	C(16)-C(21)	1.435(3)	C(1)-H(1)	0.93
N(1)-C(5)	1.345(2)	C(16)-C(17)	1.371(3)	C(20)-C(19)	1.400(3)
N(1)-C(1)	1.343(2)	C(11)-C(12)	1.380(2)	C(20)-C(25)	1.416(3)
N(3)-C(11)	1.345(2)	C(4)-H(4)	0.93	C(22)-H(22)	0.93
N(3)-C(15)	1.336(2)	C(4)-C(3)	1.382(2)	C(22)-C(23)	1.363(3)
C(5)-C(6)	1.489(2)	C(8)-C(9)	1.396(2)	C(17)-H(17)	0.93
C(5)-C(4)	1.381(2)	C(21)-C(20)	1.428(2)	C(17)-C(18)	1.406(3)

Table S4 Selected bond angles of complex **1c**

	Bond angles (°)		Bond angles (°)
Cl(2)-Mn(1)-Cl(1)	114.57(2)	C(9)-C(8)-C(7)	117.41(14)
N(2)-Mn(1)-Cl(1)	108.08(4)	C(9)-C(8)-C(16)	119.31(15)
N(2)-Mn(1)-Cl(2)	137.27(4)	C(20)-C(21)-C(16)	118.06(17)
N(2)-Mn(1)-N(1)	71.51(5)	C(22)-C(21)-C(16)	124.57(15)
N(2)-Mn(1)-N(3)	71.71(5)	C(22)-C(21)-C(20)	117.31(17)
N(1)-Mn(1)-Cl(1)	103.45(4)	C(10)-C(9)-C(8)	119.82(15)

N(1)-Mn(1)-Cl(2)	100.29(4)	C(10)-C(9)-H(9)	120.1
N(1)-Mn(1)-N(3)	141.23(5)	C(8)-C(9)-H(9)	120.1
N(3)-Mn(1)-Cl(1)	99.15(4)	C(4)-C(3)-H(3)	120.4
N(3)-Mn(1)-Cl(2)	98.33(4)	C(2)-C(3)-C(4)	119.22(17)
C(6)-N(2)-Mn(1)	120.46(10)	C(2)-C(3)-H(3)	120.4
C(10)-N(2)-Mn(1)	119.59(10)	C(3)-C(2)-H(2)	120.6
C(10)-N(2)-C(6)	119.48(13)	C(3)-C(2)-C(1)	118.76(16)
C(5)-N(1)-Mn(1)	118.16(10)	C(1)-C(2)-H(2)	120.6
C(1)-N(1)-Mn(1)	123.38(12)	N(1)-C(1)-C(2)	122.91(17)
C(1)-N(1)-C(5)	118.24(15)	N(1)-C(1)-H(1)	118.5
C(11)-N(3)-Mn(1)	117.51(11)	C(2)-C(1)-H(1)	118.5
C(15)-N(3)-Mn(1)	124.12(13)	C(19)-C(20)-C(21)	119.96(19)
C(15)-N(3)-C(11)	118.36(16)	C(19)-C(20)-C(25)	121.10(19)
N(1)-C(5)-C(6)	114.90(14)	C(25)-C(20)-C(21)	118.9(2)
N(1)-C(5)-C(4)	121.68(15)	C(21)-C(22)-H(22)	119.1
C(4)-C(5)-C(6)	123.42(15)	C(23)-C(22)-C(21)	121.76(19)
N(2)-C(6)-C(5)	114.15(14)	C(23)-C(22)-H(22)	119.1
N(2)-C(6)-C(7)	121.71(14)	C(16)-C(17)-H(17)	119.2
C(7)-C(6)-C(5)	124.14(15)	C(16)-C(17)-C(18)	121.6(2)
N(2)-C(10)-C(11)	114.40(14)	C(18)-C(17)-H(17)	119.2
N(2)-C(10)-C(9)	121.63(15)	N(3)-C(15)-H(15)	118.6
C(9)-C(10)-C(11)	123.96(14)	N(3)-C(15)-C(14)	122.87(19)
C(6)-C(7)-H(7)	120.2	C(14)-C(15)-H(15)	118.6
C(6)-C(7)-C(8)	119.66(15)	C(11)-C(12)-H(12)	120.6
C(8)-C(7)-H(7)	120.2	C(11)-C(12)-C(13)	118.89(19)
C(21)-C(16)-C(8)	123.31(15)	C(13)-C(12)-H(12)	120.6
C(17)-C(16)-C(8)	117.48(16)	C(22)-C(23)-H(23)	119.7
C(17)-C(16)-C(21)	119.20(16)	C(22)-C(23)-C(24)	120.6(2)
N(3)-C(11)-C(10)	115.08(14)	C(24)-C(23)-H(23)	119.7

N(3)-C(11)-C(12)	121.73(16)	C(12)-C(13)-H(13)	120.3
C(12)-C(11)-C(10)	123.15(16)	C(14)-C(13)-C(12)	119.39(19)
C(5)-C(4)-H(4)	120.4	C(14)-C(13)-H(13)	120.3
C(5)-C(4)-C(3)	119.18(17)	C(20)-C(19)-H(19)	119.5
C(3)-C(4)-H(4)	120.4	C(18)-C(19)-C(20)	120.97(18)
C(7)-C(8)-C(16)	123.22(15)	C(18)-C(19)-H(19)	119.5

Table S5 Selected bond lengths of complex **2a**

	Bond length (Å)		Bond length (Å)		Bond length (Å)
Mn(1)-N(2)	2.1973(14)	C(2)-H(2A)	0.93	C(12)-H(12A)	0.93
Mn(1)-N(3)	2.2540(15)	C(3)-C(4)	1.383(3)	C(13)-C(14)	1.373(3)
Mn(1)-N(1)	2.2555(16)	C(3)-H(3A)	0.93	C(13)-H(13A)	0.93
Mn(1)-Cl(1)	2.3476(6)	C(4)-C(5)	1.378(3)	C(14)-C(15)	1.378(3)
Mn(1)-Cl(2)	2.3787(6)	C(4)-H(4A)	0.93	C(14)-H(14A)	0.93
O(1)-C(19)	1.364(2)	C(5)-C(6)	1.492(2)	C(15)-H(15A)	0.93
O(1)-H(1A)	0.82	C(6)-C(7)	1.386(2)	C(16)-C(21)	1.394(3)
N(1)-C(1)	1.338(2)	C(7)-C(8)	1.399(2)	C(16)-C(17)	1.396(2)
N(1)-C(5)	1.349(2)	C(7)-H(7A)	0.93	C(17)-C(18)	1.379(2)
N(2)-C(6)	1.337(2)	C(8)-C(9)	1.393(3)	C(17)-H(17A)	0.93
N(2)-C(10)	1.346(2)	C(8)-C(16)	1.479(2)	C(18)-C(19)	1.382(3)
N(3)-C(15)	1.328(2)	C(9)-C(10)	1.383(2)	C(18)-H(18A)	0.93
N(3)-C(11)	1.346(2)	C(9)-H(9A)	0.93	C(19)-C(20)	1.383(3)
C(1)-C(2)	1.377(3)	C(10)-C(11)	1.480(3)	C(20)-C(21)	1.380(3)
C(1)-H(1B)	0.93	C(11)-C(12)	1.385(3)	C(20)-H(20A)	0.93
C(2)-C(3)	1.371(3)	C(12)-C(13)	1.375(3)	C(21)-H(21A)	0.93

Table S6 Selected bond angles of complex **2a**

	Bond angles (°)		Bond angles (°)
N(2)-Mn(1)-N(3)	72.08(5)	C(9)-C(8)-C(16)	121.62(15)

N(2)-Mn(1)-N(1)	71.98(5)	C(7)-C(8)-C(16)	121.51(16)
N(3)-Mn(1)-N(1)	144.04(5)	C(10)-C(9)-C(8)	120.40(16)
N(2)-Mn(1)-Cl(1)	129.78(4)	C(10)-C(9)-H(9A)	119.8
N(3)-Mn(1)-Cl(1)	97.74(4)	C(8)-C(9)-H(9A)	119.8
N(1)-Mn(1)-Cl(1)	104.18(4)	N(2)-C(10)-C(9)	121.47(17)
N(2)-Mn(1)-Cl(2)	117.22(4)	N(2)-C(10)-C(11)	114.48(14)
N(3)-Mn(1)-Cl(2)	99.19(4)	C(9)-C(10)-C(11)	124.04(15)
N(1)-Mn(1)-Cl(2)	98.05(4)	N(3)-C(11)-C(12)	121.01(17)
Cl(1)-Mn(1)-Cl(2)	112.92(2)	N(3)-C(11)-C(10)	115.31(15)
C(19)-O(1)-H(1A)	109.5	C(12)-C(11)-C(10)	123.69(16)
C(1)-N(1)-C(5)	117.93(16)	C(13)-C(12)-C(11)	119.15(18)
C(1)-N(1)-Mn(1)	124.28(13)	C(13)-C(12)-H(12A)	120.4
C(5)-N(1)-Mn(1)	117.70(11)	C(11)-C(12)-H(12A)	120.4
C(6)-N(2)-C(10)	119.41(14)	C(14)-C(13)-C(12)	119.78(19)
C(6)-N(2)-Mn(1)	120.37(10)	C(14)-C(13)-H(13A)	120.1
C(10)-N(2)-Mn(1)	120.09(12)	C(12)-C(13)-H(13A)	120.1
C(15)-N(3)-C(11)	118.88(16)	C(13)-C(14)-C(15)	117.96(19)
C(15)-N(3)-Mn(1)	123.42(12)	C(13)-C(14)-H(14A)	121
C(11)-N(3)-Mn(1)	117.40(12)	C(15)-C(14)-H(14A)	121
N(1)-C(1)-C(2)	123.43(18)	N(3)-C(15)-C(14)	123.13(18)
N(1)-C(1)-H(1B)	118.3	N(3)-C(15)-H(15A)	118.4
C(2)-C(1)-H(1B)	118.3	C(14)-C(15)-H(15A)	118.4
C(3)-C(2)-C(1)	118.33(17)	C(21)-C(16)-C(17)	117.20(15)
C(3)-C(2)-H(2A)	120.8	C(21)-C(16)-C(8)	121.71(15)
C(1)-C(2)-H(2A)	120.8	C(17)-C(16)-C(8)	121.07(16)
C(2)-C(3)-C(4)	119.23(19)	C(18)-C(17)-C(16)	121.55(17)
C(2)-C(3)-H(3A)	120.4	C(18)-C(17)-H(17A)	119.2
C(4)-C(3)-H(3A)	120.4	C(16)-C(17)-H(17A)	119.2
C(5)-C(4)-C(3)	119.38(18)	C(17)-C(18)-C(19)	119.98(16)

C(5)-C(4)-H(4A)	120.3	C(17)-C(18)-H(18A)	120
C(3)-C(4)-H(4A)	120.3	C(19)-C(18)-H(18A)	120
N(1)-C(5)-C(4)	121.70(15)	O(1)-C(19)-C(18)	122.77(16)
N(1)-C(5)-C(6)	114.92(16)	O(1)-C(19)-C(20)	117.48(18)
C(4)-C(5)-C(6)	123.37(16)	C(18)-C(19)-C(20)	119.74(16)
N(2)-C(6)-C(7)	121.68(15)	C(21)-C(20)-C(19)	119.86(18)
N(2)-C(6)-C(5)	114.62(14)	C(21)-C(20)-H(20A)	120.1
C(7)-C(6)-C(5)	123.70(16)	C(19)-C(20)-H(20A)	120.1
C(6)-C(7)-C(8)	120.15(17)	C(20)-C(21)-C(16)	121.64(17)
C(6)-C(7)-H(7A)	119.9	C(20)-C(21)-H(21A)	119.2
C(8)-C(7)-H(7A)	119.9	C(16)-C(21)-H(21A)	119.2
C(9)-C(8)-C(7)	116.86(15)		

Table S7 Selected bond lengths of complex **2b**

	Bond length (Å)		Bond length (Å)		Bond length (Å)
Mn(1)-N(2)	2.1971(15)	C(2)-H(2A)	0.93	C(12)-H(12A)	0.93
Mn(1)-N(1)	2.2494(16)	C(3)-C(4)	1.381(3)	C(13)-C(14)	1.368(3)
Mn(1)-N(3)	2.2550(16)	C(3)-H(3A)	0.93	C(13)-H(13A)	0.93
Mn(1)-Cl(1)	2.3442(6)	C(4)-C(5)	1.379(3)	C(14)-C(15)	1.376(3)
Mn(1)-Cl(2)	2.3867(7)	C(4)-H(4A)	0.93	C(14)-H(14A)	0.93
O(1)-C(18)	1.366(2)	C(5)-C(6)	1.486(2)	C(15)-H(15A)	0.93
O(1)-H(1A)	0.82	C(6)-C(7)	1.388(2)	C(16)-C(21)	1.387(3)
N(1)-C(1)	1.342(2)	C(7)-C(8)	1.393(3)	C(16)-C(17)	1.392(3)
N(1)-C(5)	1.348(2)	C(7)-H(7A)	0.93	C(17)-C(18)	1.385(3)
N(2)-C(6)	1.334(2)	C(8)-C(9)	1.389(3)	C(17)-H(17A)	0.93
N(2)-C(10)	1.343(2)	C(8)-C(16)	1.488(2)	C(18)-C(19)	1.378(3)
N(3)-C(15)	1.332(3)	C(9)-C(10)	1.384(2)	C(19)-C(20)	1.377(3)
N(3)-C(11)	1.348(2)	C(9)-H(9A)	0.93	C(19)-H(19A)	0.93
C(1)-C(2)	1.369(3)	C(10)-C(11)	1.480(3)	C(20)-C(21)	1.389(3)

C(1)-H(1B)	0.93	C(11)-C(12)	1.380(3)	C(20)-H(20A)	0.93
C(2)-C(3)	1.371(3)	C(12)-C(13)	1.379(3)	C(21)-H(21A)	0.93

Table S8 Selected bond angles of complex **2b**

	Bond angles (°)		Bond angles (°)
N(2)-Mn(1)-N(1)	72.05(5)	C(9)-C(8)-C(16)	121.15(17)
N(2)-Mn(1)-N(3)	72.00(6)	C(7)-C(8)-C(16)	121.47(17)
N(1)-Mn(1)-N(3)	144.05(5)	C(10)-C(9)-C(8)	119.98(17)
N(2)-Mn(1)-Cl(1)	128.26(5)	C(10)-C(9)-H(9A)	120
N(1)-Mn(1)-Cl(1)	102.98(5)	C(8)-C(9)-H(9A)	120
N(3)-Mn(1)-Cl(1)	99.25(5)	N(2)-C(10)-C(9)	121.66(17)
N(2)-Mn(1)-Cl(2)	116.90(5)	N(2)-C(10)-C(11)	114.82(15)
N(1)-Mn(1)-Cl(2)	96.47(5)	C(9)-C(10)-C(11)	123.46(16)
N(3)-Mn(1)-Cl(2)	99.39(5)	N(3)-C(11)-C(12)	121.54(18)
Cl(1)-Mn(1)-Cl(2)	114.84(2)	N(3)-C(11)-C(10)	114.89(15)
C(18)-O(1)-H(1A)	109.5	C(12)-C(11)-C(10)	123.56(16)
C(1)-N(1)-C(5)	118.12(17)	C(13)-C(12)-C(11)	118.94(19)
C(1)-N(1)-Mn(1)	124.19(13)	C(13)-C(12)-H(12A)	120.5
C(5)-N(1)-Mn(1)	117.63(12)	C(11)-C(12)-H(12A)	120.5
C(6)-N(2)-C(10)	119.45(15)	C(14)-C(13)-C(12)	119.6(2)
C(6)-N(2)-Mn(1)	120.08(11)	C(14)-C(13)-H(13A)	120.2
C(10)-N(2)-Mn(1)	120.05(12)	C(12)-C(13)-H(13A)	120.2
C(15)-N(3)-C(11)	118.38(17)	C(13)-C(14)-C(15)	118.3(2)
C(15)-N(3)-Mn(1)	123.82(13)	C(13)-C(14)-H(14A)	120.8
C(11)-N(3)-Mn(1)	117.68(12)	C(15)-C(14)-H(14A)	120.8
N(1)-C(1)-C(2)	123.04(19)	N(3)-C(15)-C(14)	123.08(19)
N(1)-C(1)-H(1B)	118.5	N(3)-C(15)-H(15A)	118.5
C(2)-C(1)-H(1B)	118.5	C(14)-C(15)-H(15A)	118.5
C(1)-C(2)-C(3)	118.78(19)	C(21)-C(16)-C(17)	118.57(17)

C(1)-C(2)-H(2A)	120.6	C(21)-C(16)-C(8)	121.58(18)
C(3)-C(2)-H(2A)	120.6	C(17)-C(16)-C(8)	119.82(18)
C(2)-C(3)-C(4)	119.16(19)	C(18)-C(17)-C(16)	120.87(19)
C(2)-C(3)-H(3A)	120.4	C(18)-C(17)-H(17A)	119.6
C(4)-C(3)-H(3A)	120.4	C(16)-C(17)-H(17A)	119.6
C(3)-C(4)-C(5)	119.32(19)	O(1)-C(18)-C(19)	123.46(17)
C(3)-C(4)-H(4A)	120.3	O(1)-C(18)-C(17)	116.31(18)
C(5)-C(4)-H(4A)	120.3	C(19)-C(18)-C(17)	120.23(19)
N(1)-C(5)-C(4)	121.59(17)	C(18)-C(19)-C(20)	119.19(18)
N(1)-C(5)-C(6)	115.06(16)	C(18)-C(19)-H(19A)	120.4
C(4)-C(5)-C(6)	123.35(17)	C(20)-C(19)-H(19A)	120.4
N(2)-C(6)-C(7)	121.53(16)	C(19)-C(20)-C(21)	121.1(2)
N(2)-C(6)-C(5)	114.76(15)	C(19)-C(20)-H(20A)	119.5
C(7)-C(6)-C(5)	123.68(17)	C(21)-C(20)-H(20A)	119.5
C(6)-C(7)-C(8)	120.03(17)	C(20)-C(21)-C(16)	120.0(2)
C(6)-C(7)-H(7A)	120	C(20)-C(21)-H(21A)	120
C(8)-C(7)-H(7A)	120	C(16)-C(21)-H(21A)	120
C(9)-C(8)-C(7)	117.33(16)		

Table S9 Selected bond lengths of complex **2c**

	Bond length (Å)		Bond length (Å)		Bond length (Å)
Mn(1)-N(2)	2.1838(18)	N(3)-C(11)	1.346(3)	C(9)-H(9A)	0.93
Mn(1)-N(3)	2.2444(19)	C(1)-C(2)	1.374(3)	C(10)-C(11)	1.486(3)
Mn(1)-N(1)	2.2456(18)	C(1)-H(1B)	0.93	C(11)-C(12)	1.384(3)
Mn(1)-Cl(1)	2.3392(11)	C(2)-C(3)	1.369(3)	C(12)-C(13)	1.382(3)
Mn(1)-Cl(2)	2.3651(9)	C(2)-H(2A)	0.93	C(12)-H(12A)	0.93
O(1)-C(17)	1.354(3)	C(3)-C(4)	1.382(3)	C(13)-C(14)	1.372(4)
O(1)-H(1A)	0.82	C(3)-H(3A)	0.93	C(13)-H(13A)	0.93
O(2)-H(02E)	1.05(4)	C(4)-C(5)	1.379(3)	C(14)-C(15)	1.361(3)

O(2)-H(02F)	1.22(10)	C(4)-H(4A)	0.93	C(14)-H(14A)	0.93
O(3)-H(03E)	0.86(5)	C(5)-C(6)	1.487(3)	C(15)-H(15A)	0.93
O(3)-H(03F)	0.77(4)	C(6)-C(7)	1.384(3)	C(16)-C(21)	1.388(3)
N(1)-C(1)	1.339(3)	C(7)-C(8)	1.395(3)	C(16)-C(17)	1.398(3)
N(1)-C(5)	1.347(2)	C(7)-H(7A)	0.93	C(17)-C(18)	1.387(3)
N(2)-C(6)	1.342(2)	C(8)-C(9)	1.396(3)	C(18)-C(19)	1.370(4)
N(2)-C(10)	1.345(2)	C(8)-C(16)	1.479(3)	C(18)-H(18A)	0.93
N(3)-C(15)	1.340(3)	C(9)-C(10)	1.380(3)	C(19)-C(20)	1.373(4)

Table S10 Selected bond angles of complex **2c**

	Bond angles (°)		Bond angles (°)
N(2)-Mn(1)-N(3)	72.30(7)	C(8)-C(7)-H(7A)	119.9
N(2)-Mn(1)-N(1)	72.43(6)	C(7)-C(8)-C(9)	117.45(17)
N(3)-Mn(1)-N(1)	143.68(6)	C(7)-C(8)-C(16)	120.54(18)
N(2)-Mn(1)-Cl(1)	135.65(5)	C(9)-C(8)-C(16)	121.99(17)
N(3)-Mn(1)-Cl(1)	98.52(6)	C(10)-C(9)-C(8)	119.86(17)
N(1)-Mn(1)-Cl(1)	100.55(5)	C(10)-C(9)-H(9A)	120.1
N(2)-Mn(1)-Cl(2)	110.29(5)	C(8)-C(9)-H(9A)	120.1
N(3)-Mn(1)-Cl(2)	99.96(5)	N(2)-C(10)-C(9)	121.58(17)
N(1)-Mn(1)-Cl(2)	100.03(5)	N(2)-C(10)-C(11)	114.39(16)
Cl(1)-Mn(1)-Cl(2)	114.04(4)	C(9)-C(10)-C(11)	124.02(17)
C(17)-O(1)-H(1A)	109.5	N(3)-C(11)-C(12)	121.29(18)
H(02E)-O(2)-H(02F)	89(4)	N(3)-C(11)-C(10)	115.12(16)
H(03E)-O(3)-H(03F)	125(4)	C(12)-C(11)-C(10)	123.59(18)
C(1)-N(1)-C(5)	118.33(18)	C(13)-C(12)-C(11)	119.1(2)
C(1)-N(1)-Mn(1)	124.23(15)	C(13)-C(12)-H(12A)	120.5
C(5)-N(1)-Mn(1)	117.34(12)	C(11)-C(12)-H(12A)	120.5
C(6)-N(2)-C(10)	119.76(16)	C(14)-C(13)-C(12)	119.2(2)
C(6)-N(2)-Mn(1)	120.06(12)	C(14)-C(13)-H(13A)	120.4

C(10)-N(2)-Mn(1)	119.98(12)	C(12)-C(13)-H(13A)	120.4
C(15)-N(3)-C(11)	118.47(18)	C(15)-C(14)-C(13)	118.9(2)
C(15)-N(3)-Mn(1)	123.78(15)	C(15)-C(14)-H(14A)	120.6
C(11)-N(3)-Mn(1)	117.39(13)	C(13)-C(14)-H(14A)	120.6
N(1)-C(1)-C(2)	123.0(2)	N(3)-C(15)-C(14)	123.1(2)
N(1)-C(1)-H(1B)	118.5	N(3)-C(15)-H(15A)	118.5
C(2)-C(1)-H(1B)	118.5	C(14)-C(15)-H(15A)	118.5
C(3)-C(2)-C(1)	118.6(2)	C(21)-C(16)-C(17)	118.29(19)
C(3)-C(2)-H(2A)	120.7	C(21)-C(16)-C(8)	119.94(19)
C(1)-C(2)-H(2A)	120.7	C(17)-C(16)-C(8)	121.76(19)
C(2)-C(3)-C(4)	119.3(2)	O(1)-C(17)-C(18)	121.5(2)
C(2)-C(3)-H(3A)	120.3	O(1)-C(17)-C(16)	118.49(18)
C(4)-C(3)-H(3A)	120.3	C(18)-C(17)-C(16)	120.0(2)
C(5)-C(4)-C(3)	119.3(2)	C(19)-C(18)-C(17)	120.4(2)
C(5)-C(4)-H(4A)	120.4	C(19)-C(18)-H(18A)	119.8
C(3)-C(4)-H(4A)	120.4	C(17)-C(18)-H(18A)	119.8
N(1)-C(5)-C(4)	121.49(17)	C(18)-C(19)-C(20)	120.7(2)
N(1)-C(5)-C(6)	115.23(17)	C(18)-C(19)-H(19A)	119.6
C(4)-C(5)-C(6)	123.28(17)	C(20)-C(19)-H(19A)	119.6
N(2)-C(6)-C(7)	121.17(17)	C(19)-C(20)-C(21)	119.2(3)
N(2)-C(6)-C(5)	114.49(16)	C(19)-C(20)-H(20A)	120.4
C(7)-C(6)-C(5)	124.34(17)	C(21)-C(20)-H(20A)	120.4
C(6)-C(7)-C(8)	120.17(18)	C(20)-C(21)-C(16)	121.4(2)
C(6)-C(7)-H(7A)	119.9		

Table S11 Selected bond lengths of complex **2f**

	Bond length (Å)		Bond length (Å)		Bond length (Å)
Mn(1)-N(2)	2.2005(17)	C(2)-H(2B)	0.93	C(13)-H(13A)	0.93
Mn(1)-N(3)	2.252(2)	C(3)-C(4)	1.378(3)	C(14)-C(15)	1.362(4)

Mn(1)-N(1)	2.2550(18)	C(3)-H(3A)	0.93	C(14)-H(14A)	0.93
Mn(1)-Cl(1)	2.3243(7)	C(4)-C(5)	1.381(3)	C(15)-H(15A)	0.93
Mn(1)-Cl(2)	2.3582(7)	C(4)-H(4A)	0.93	C(16)-C(17)	1.386(3)
S(1)-O(1)	1.4314(18)	C(5)-C(6)	1.482(3)	C(16)-C(21)	1.391(3)
S(1)-O(2)	1.4437(19)	C(6)-C(7)	1.385(3)	C(17)-C(18)	1.380(3)
S(1)-C(22)	1.737(3)	C(7)-C(8)	1.391(3)	C(17)-H(17A)	0.93
S(1)-C(19)	1.767(2)	C(7)-H(7A)	0.93	C(18)-C(19)	1.375(3)
N(1)-C(1)	1.337(3)	C(8)-C(9)	1.396(3)	C(18)-H(18A)	0.93
N(1)-C(5)	1.343(3)	C(8)-C(16)	1.489(3)	C(19)-C(20)	1.380(3)
N(2)-C(6)	1.339(3)	C(9)-C(10)	1.380(3)	C(20)-C(21)	1.383(3)
N(2)-C(10)	1.342(3)	C(9)-H(9A)	0.93	C(20)-H(20A)	0.93
N(3)-C(15)	1.337(3)	C(10)-C(11)	1.487(3)	C(21)-H(21A)	0.93
N(3)-C(11)	1.347(3)	C(11)-C(12)	1.378(3)	C(22)-H(22A)	0.96
C(1)-C(2)	1.372(3)	C(12)-C(13)	1.383(3)	C(22)-H(22B)	0.96
C(1)-H(1B)	0.93	C(12)-H(12A)	0.93	C(22)-H(22C)	0.96
C(2)-C(3)	1.376(4)	C(13)-C(14)	1.373(4)		

Table S12 Selected bond angles of complex **2f**

	Bond angles (°)		Bond angles (°)
N(2)-Mn(1)-N(1)	72.05(5)	C(9)-C(8)-C(16)	121.15(17)
N(2)-Mn(1)-N(3)	72.00(6)	C(7)-C(8)-C(16)	121.47(17)
N(1)-Mn(1)-N(3)	144.05(5)	C(10)-C(9)-C(8)	119.98(17)
N(2)-Mn(1)-Cl(1)	128.26(5)	C(10)-C(9)-H(9A)	120
N(1)-Mn(1)-Cl(1)	102.98(5)	C(8)-C(9)-H(9A)	120
N(3)-Mn(1)-Cl(1)	99.25(5)	N(2)-C(10)-C(9)	121.66(17)
N(2)-Mn(1)-Cl(2)	116.90(5)	N(2)-C(10)-C(11)	114.82(15)
N(1)-Mn(1)-Cl(2)	96.47(5)	C(9)-C(10)-C(11)	123.46(16)
N(3)-Mn(1)-Cl(2)	99.39(5)	N(3)-C(11)-C(12)	121.54(18)
Cl(1)-Mn(1)-Cl(2)	114.84(2)	N(3)-C(11)-C(10)	114.89(15)

C(18)-O(1)-H(1A)	109.5	C(12)-C(11)-C(10)	123.56(16)
C(1)-N(1)-C(5)	118.12(17)	C(13)-C(12)-C(11)	118.94(19)
C(1)-N(1)-Mn(1)	124.19(13)	C(13)-C(12)-H(12A)	120.5
C(5)-N(1)-Mn(1)	117.63(12)	C(11)-C(12)-H(12A)	120.5
C(6)-N(2)-C(10)	119.45(15)	C(14)-C(13)-C(12)	119.6(2)
C(6)-N(2)-Mn(1)	120.08(11)	C(14)-C(13)-H(13A)	120.2
C(10)-N(2)-Mn(1)	120.05(12)	C(12)-C(13)-H(13A)	120.2
C(15)-N(3)-C(11)	118.38(17)	C(13)-C(14)-C(15)	118.3(2)
C(15)-N(3)-Mn(1)	123.82(13)	C(13)-C(14)-H(14A)	120.8
C(11)-N(3)-Mn(1)	117.68(12)	C(15)-C(14)-H(14A)	120.8
N(1)-C(1)-C(2)	123.04(19)	N(3)-C(15)-C(14)	123.08(19)
N(1)-C(1)-H(1B)	118.5	N(3)-C(15)-H(15A)	118.5
C(2)-C(1)-H(1B)	118.5	C(14)-C(15)-H(15A)	118.5
C(1)-C(2)-C(3)	118.78(19)	C(21)-C(16)-C(17)	118.57(17)
C(1)-C(2)-H(2A)	120.6	C(21)-C(16)-C(8)	121.58(18)
C(3)-C(2)-H(2A)	120.6	C(17)-C(16)-C(8)	119.82(18)
C(2)-C(3)-C(4)	119.16(19)	C(18)-C(17)-C(16)	120.87(19)
C(2)-C(3)-H(3A)	120.4	C(18)-C(17)-H(17A)	119.6
C(4)-C(3)-H(3A)	120.4	C(16)-C(17)-H(17A)	119.6
C(3)-C(4)-C(5)	119.32(19)	O(1)-C(18)-C(19)	123.46(17)
C(3)-C(4)-H(4A)	120.3	O(1)-C(18)-C(17)	116.31(18)
C(5)-C(4)-H(4A)	120.3	C(19)-C(18)-C(17)	120.23(19)
N(1)-C(5)-C(4)	121.59(17)	C(18)-C(19)-C(20)	119.19(18)
N(1)-C(5)-C(6)	115.06(16)	C(18)-C(19)-H(19A)	120.4
C(4)-C(5)-C(6)	123.35(17)	C(20)-C(19)-H(19A)	120.4
N(2)-C(6)-C(7)	121.53(16)	C(19)-C(20)-C(21)	121.1(2)
N(2)-C(6)-C(5)	114.76(15)	C(19)-C(20)-H(20A)	119.5
C(7)-C(6)-C(5)	123.68(17)	C(21)-C(20)-H(20A)	119.5
C(6)-C(7)-C(8)	120.03(17)	C(20)-C(21)-C(16)	120.0(2)

C(6)-C(7)-H(7A)	120	C(20)-C(21)-H(21A)	120
C(8)-C(7)-H(7A)	120	C(16)-C(21)-H(21A)	120
C(9)-C(8)-C(7)	117.33(16)		

Table S13 Selected bond lengths of complex **2g**

	Bond length (Å)		Bond length (Å)		Bond length (Å)
Mn(1)-N(2)	2.198(3)	C(9)-C(7)	1.394(3)	Mn(1)-N(2)	2.198(3)
Mn(1)-N(1)	2.251(2)	C(9)-C(7)	1.394(3)	Mn(1)-N(1)	2.251(2)
Mn(1)-N(1)	2.251(2)	C(9)-C(10)	1.479(5)	Mn(1)-N(1)	2.251(2)
Mn(1)-Cl(1B)	2.291(4)	C(7)-C(6)	1.384(4)	Mn(1)-Cl(1B)	2.291(4)
Mn(1)-Cl(1B)	2.291(4)	C(4)-C(3)	1.377(5)	Mn(1)-Cl(1B)	2.291(4)
Mn(1)-Cl(1A)	2.361(5)	C(10)-C(11)	1.385(4)	Mn(1)-Cl(1A)	2.361(5)
Mn(1)-Cl(1A)	2.361(5)	C(10)-C(11)	1.385(4)	Mn(1)-Cl(1A)	2.361(5)
N(2)-C(6)	1.345(3)	C(2)-C(3)	1.375(5)	N(2)-C(6)	1.345(3)
N(2)-C(6)	1.345(3)	C(11)-C(12)	1.389(4)	N(2)-C(6)	1.345(3)
N(1)-C(1)	1.333(3)	N(3)-O(1)	1.215(3)	N(1)-C(1)	1.333(3)
N(1)-C(5)	1.343(3)	N(3)-O(1)	1.215(3)	N(1)-C(5)	1.343(3)
C(1)-C(2)	1.377(4)	N(3)-C(13)	1.464(5)	C(1)-C(2)	1.377(4)
C(5)-C(4)	1.386(4)	C(13)-C(12)	1.371(4)	C(5)-C(4)	1.386(4)
C(5)-C(6)	1.487(3)	C(13)-C(12)	1.371(4)	C(5)-C(6)	1.487(3)

Table S14 Selected bond angles of complex **2g**

	Bond angles (°)		Bond angles (°)
N(2)-Mn(1)-N(1)	72.05(5)	C(9)-C(8)-C(16)	121.15(17)
N(2)-Mn(1)-N(3)	72.00(6)	C(7)-C(8)-C(16)	121.47(17)
N(1)-Mn(1)-N(3)	144.05(5)	C(10)-C(9)-C(8)	119.98(17)
N(2)-Mn(1)-Cl(1)	128.26(5)	C(10)-C(9)-H(9A)	120
N(1)-Mn(1)-Cl(1)	102.98(5)	C(8)-C(9)-H(9A)	120
N(3)-Mn(1)-Cl(1)	99.25(5)	N(2)-C(10)-C(9)	121.66(17)

N(2)-Mn(1)-Cl(2)	116.90(5)	N(2)-C(10)-C(11)	114.82(15)
N(1)-Mn(1)-Cl(2)	96.47(5)	C(9)-C(10)-C(11)	123.46(16)
N(3)-Mn(1)-Cl(2)	99.39(5)	N(3)-C(11)-C(12)	121.54(18)
Cl(1)-Mn(1)-Cl(2)	114.84(2)	N(3)-C(11)-C(10)	114.89(15)
C(18)-O(1)-H(1A)	109.5	C(12)-C(11)-C(10)	123.56(16)
C(1)-N(1)-C(5)	118.12(17)	C(13)-C(12)-C(11)	118.94(19)
C(1)-N(1)-Mn(1)	124.19(13)	C(13)-C(12)-H(12A)	120.5
C(5)-N(1)-Mn(1)	117.63(12)	C(11)-C(12)-H(12A)	120.5
C(6)-N(2)-C(10)	119.45(15)	C(14)-C(13)-C(12)	119.6(2)
C(6)-N(2)-Mn(1)	120.08(11)	C(14)-C(13)-H(13A)	120.2
C(10)-N(2)-Mn(1)	120.05(12)	C(12)-C(13)-H(13A)	120.2
C(15)-N(3)-C(11)	118.38(17)	C(13)-C(14)-C(15)	118.3(2)
C(15)-N(3)-Mn(1)	123.82(13)	C(13)-C(14)-H(14A)	120.8
C(11)-N(3)-Mn(1)	117.68(12)	C(15)-C(14)-H(14A)	120.8
N(1)-C(1)-C(2)	123.04(19)	N(3)-C(15)-C(14)	123.08(19)
N(1)-C(1)-H(1B)	118.5	N(3)-C(15)-H(15A)	118.5
C(2)-C(1)-H(1B)	118.5	C(14)-C(15)-H(15A)	118.5
C(1)-C(2)-C(3)	118.78(19)	C(21)-C(16)-C(17)	118.57(17)
C(1)-C(2)-H(2A)	120.6	C(21)-C(16)-C(8)	121.58(18)
C(3)-C(2)-H(2A)	120.6	C(17)-C(16)-C(8)	119.82(18)
C(2)-C(3)-C(4)	119.16(19)	C(18)-C(17)-C(16)	120.87(19)
C(2)-C(3)-H(3A)	120.4	C(18)-C(17)-H(17A)	119.6
C(4)-C(3)-H(3A)	120.4	C(16)-C(17)-H(17A)	119.6
C(3)-C(4)-C(5)	119.32(19)	O(1)-C(18)-C(19)	123.46(17)
C(3)-C(4)-H(4A)	120.3	O(1)-C(18)-C(17)	116.31(18)
C(5)-C(4)-H(4A)	120.3	C(19)-C(18)-C(17)	120.23(19)
N(1)-C(5)-C(4)	121.59(17)	C(18)-C(19)-C(20)	119.19(18)
N(1)-C(5)-C(6)	115.06(16)	C(18)-C(19)-H(19A)	120.4
C(4)-C(5)-C(6)	123.35(17)	C(20)-C(19)-H(19A)	120.4

N(2)-C(6)-C(7)	121.53(16)	C(19)-C(20)-C(21)	121.1(2)
N(2)-C(6)-C(5)	114.76(15)	C(19)-C(20)-H(20A)	119.5
C(7)-C(6)-C(5)	123.68(17)	C(21)-C(20)-H(20A)	119.5
C(6)-C(7)-C(8)	120.03(17)	C(20)-C(21)-C(16)	120.0(2)
C(6)-C(7)-H(7A)	120	C(20)-C(21)-H(21A)	120
C(8)-C(7)-H(7A)	120	C(16)-C(21)-H(21A)	120
C(9)-C(8)-C(7)	117.33(16)		

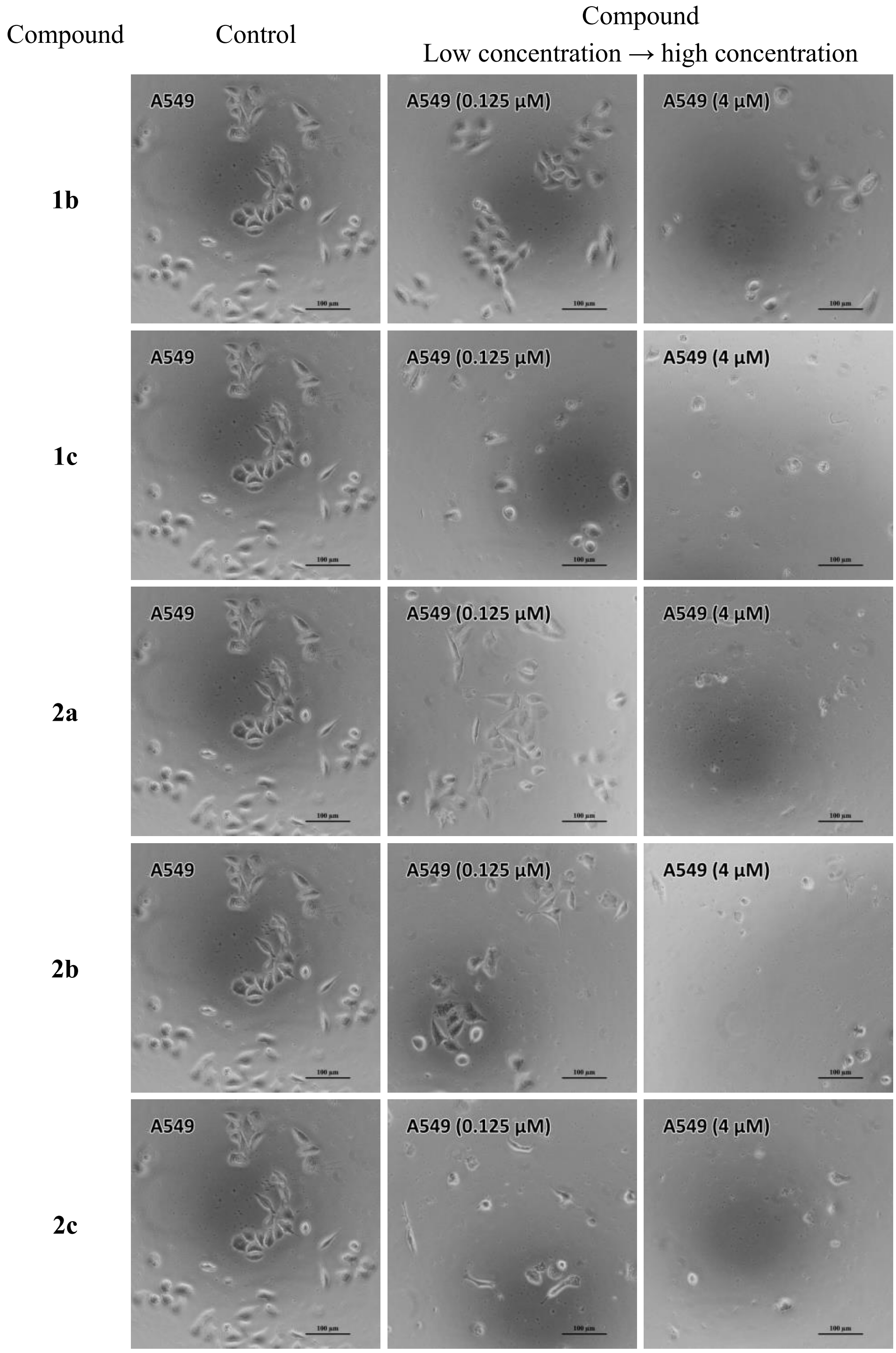

Figure S25 The microscopic images of A549 cells treated with increased concentrations of the compounds **1b**, **1c** and **2a**–**2c**.

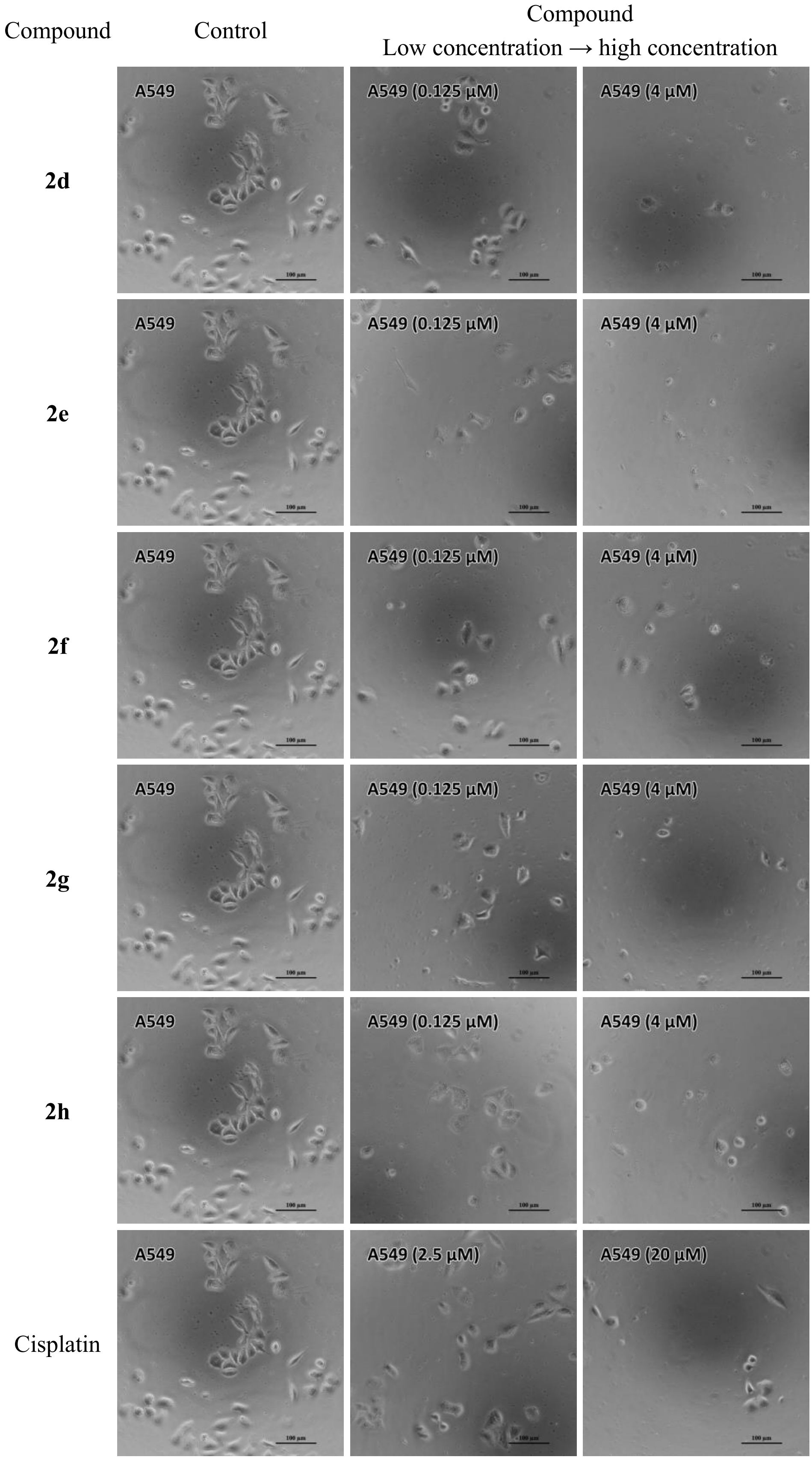

Figure S26 The microscopic images of A549 cells treated with increased concentrations of the compounds **2d**–**2h** and cisplatin.

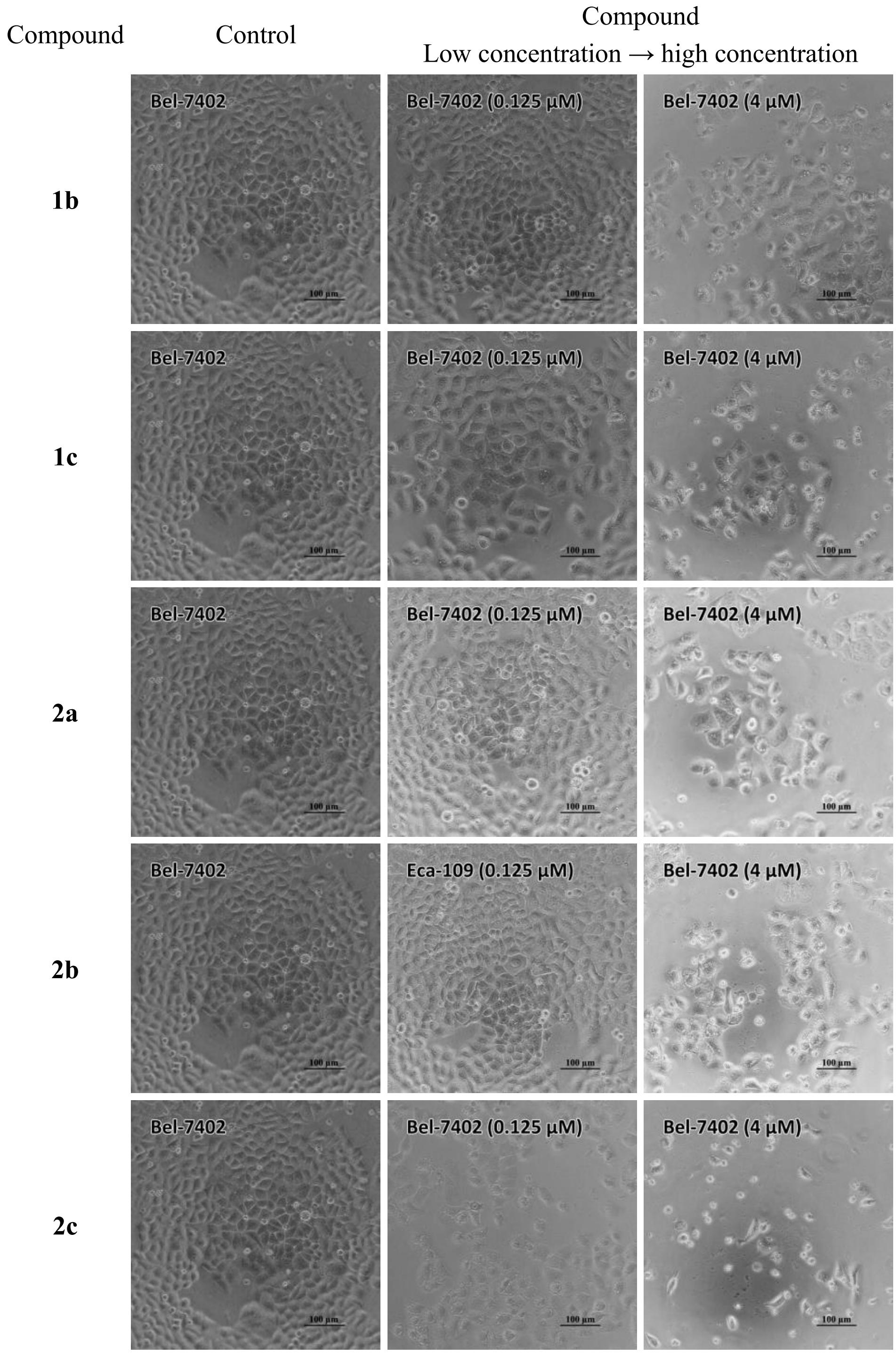

Figure S27 The microscopic images of Bel-7402 cells treated with increased concentrations of the compounds **1b**, **1c** and **2a**–**2c**.

Compound | Control | Compound Low concentration → high concentration

2d

2e

2f

2g

2h

Cisplatin

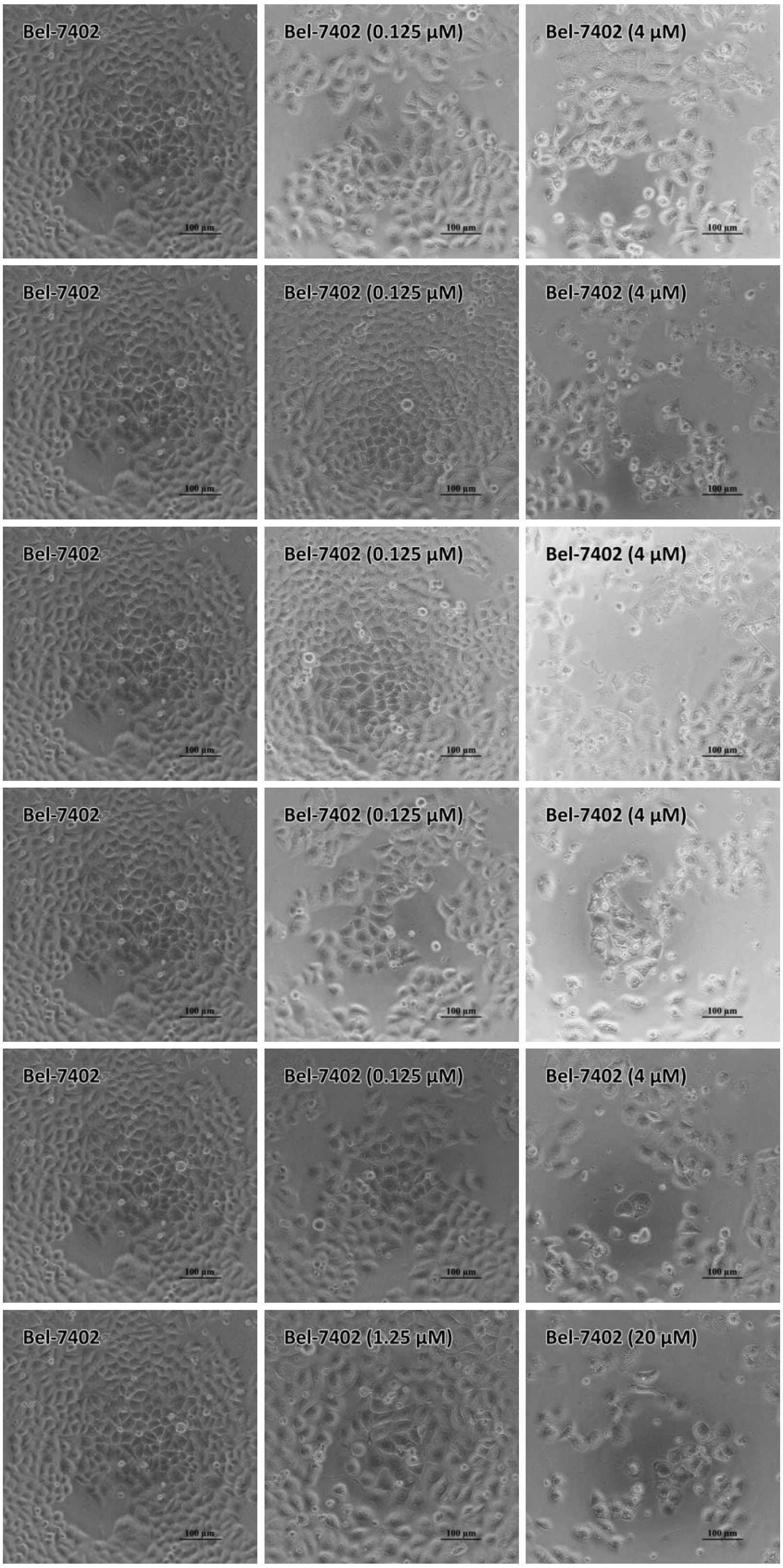

Figure S28 The microscopic images of A549 cells treated with increased concentrations of the compounds **2d**–**2h** and cisplatin.

Compound Control Compound

Low concentration → high concentration

1b

1c

2a

2b

2c

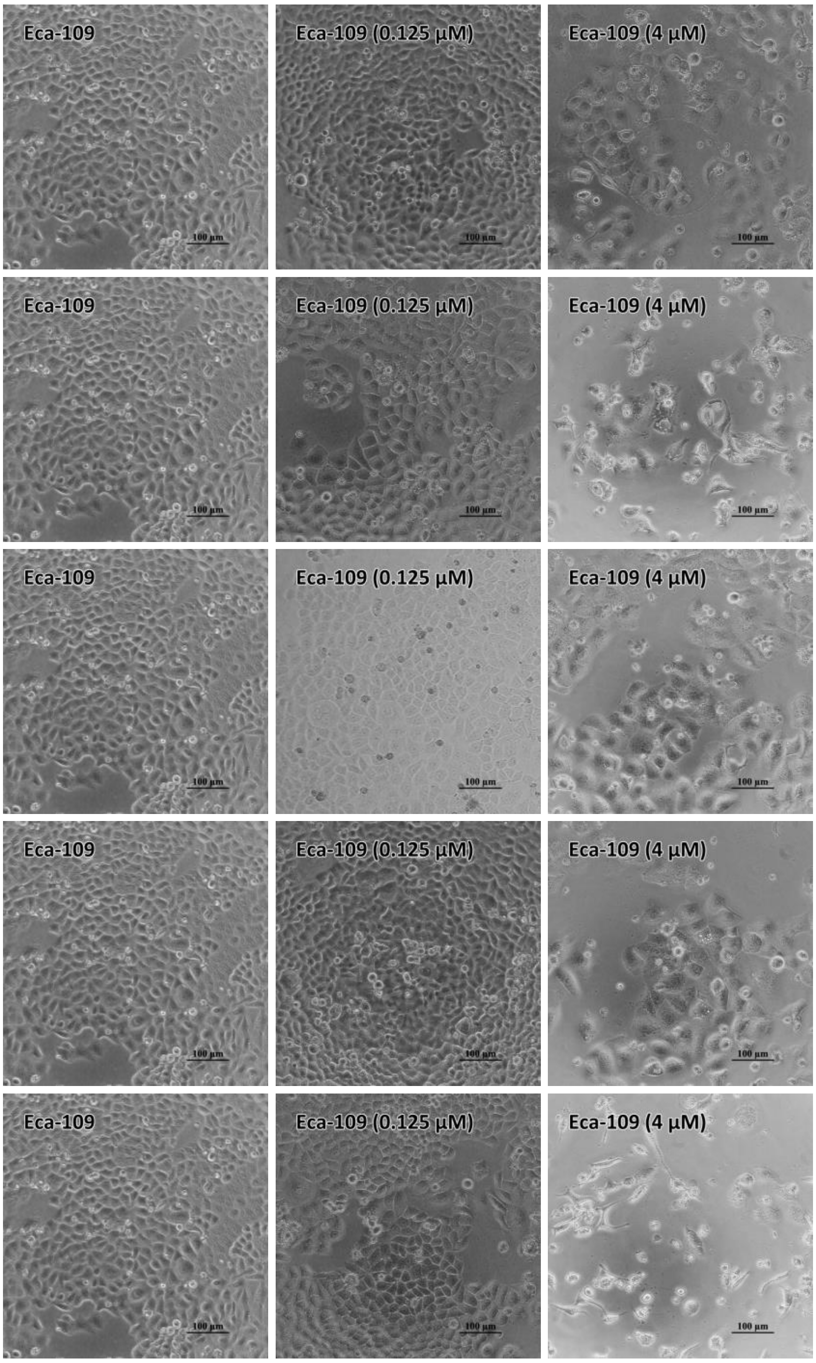

Figure S29 The microscopic images of Eca-109 cells treated with increased concentrations of the compounds **1b**, **1c** and **2a**–**2c**.

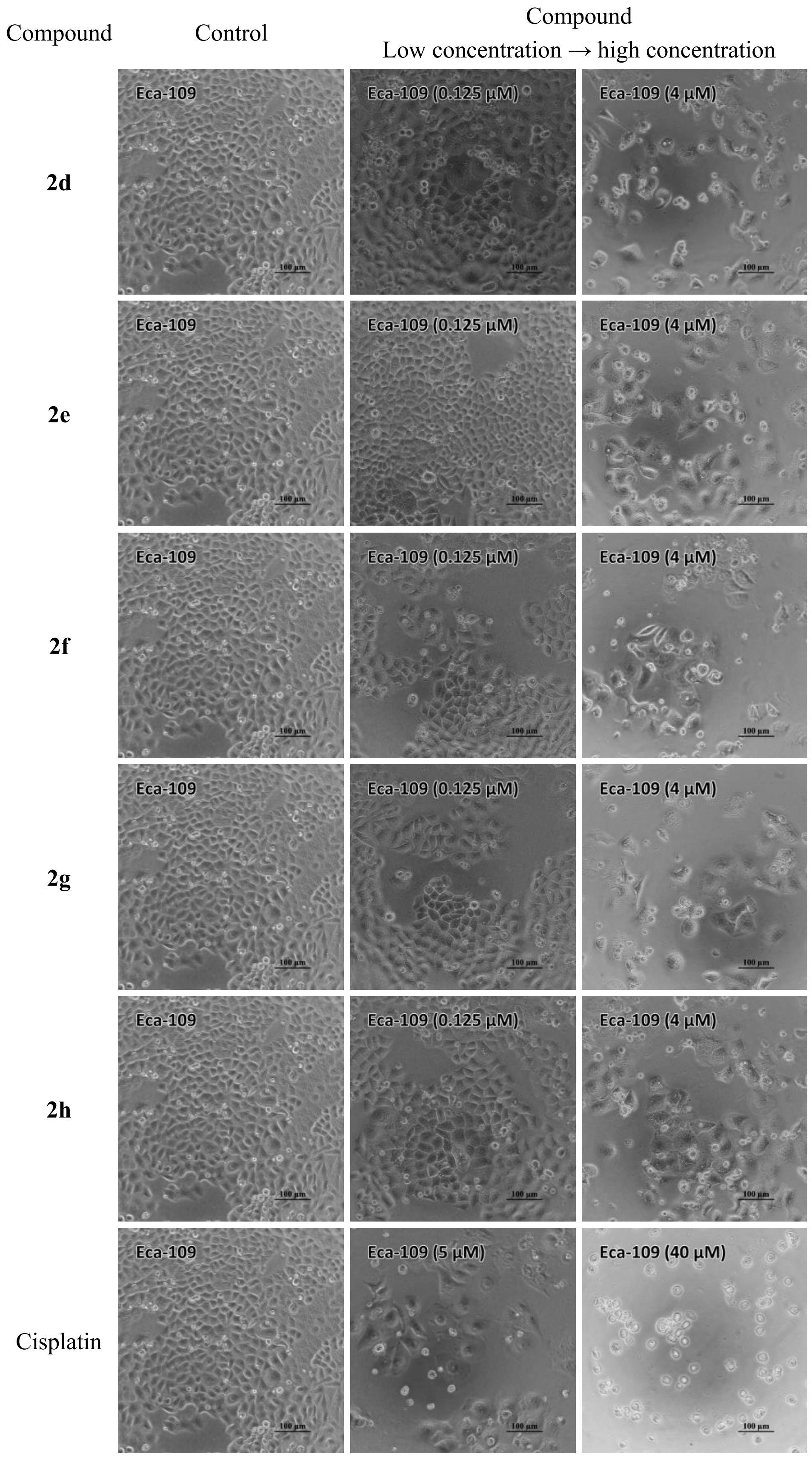

Figure S30 The microscopic images of Eca-109 cells treated with increased concentrations of the compounds **2d**–**2h** and cisplatin.

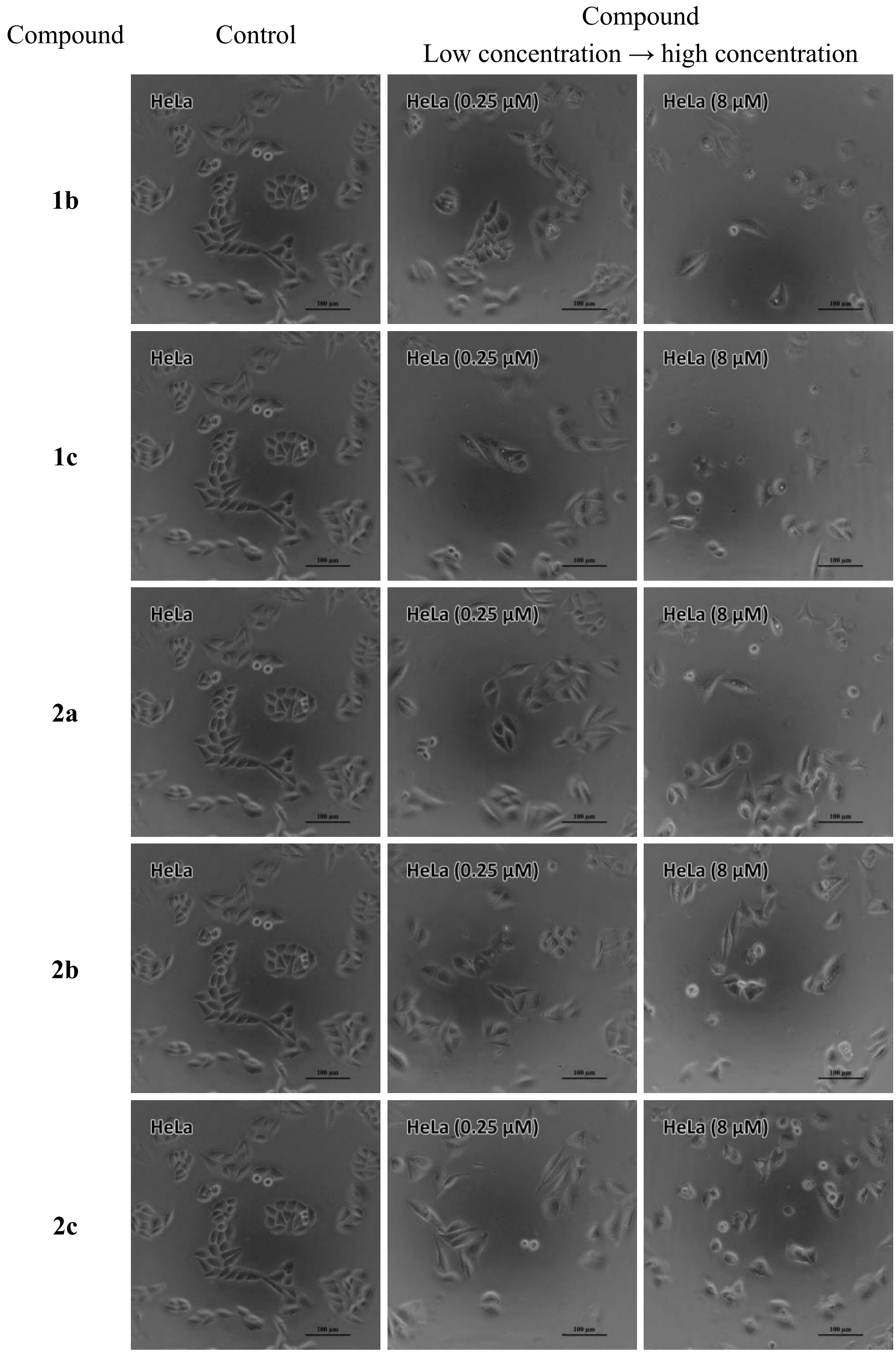

Figure S31 The microscopic images of HeLa cells treated with increased concentrations of the compounds **1b**, **1c** and **2a**–**2c**.

Compound | Control | Compound Low concentration → high concentration

2d

2e

2f

2g

2h

Cisplatin

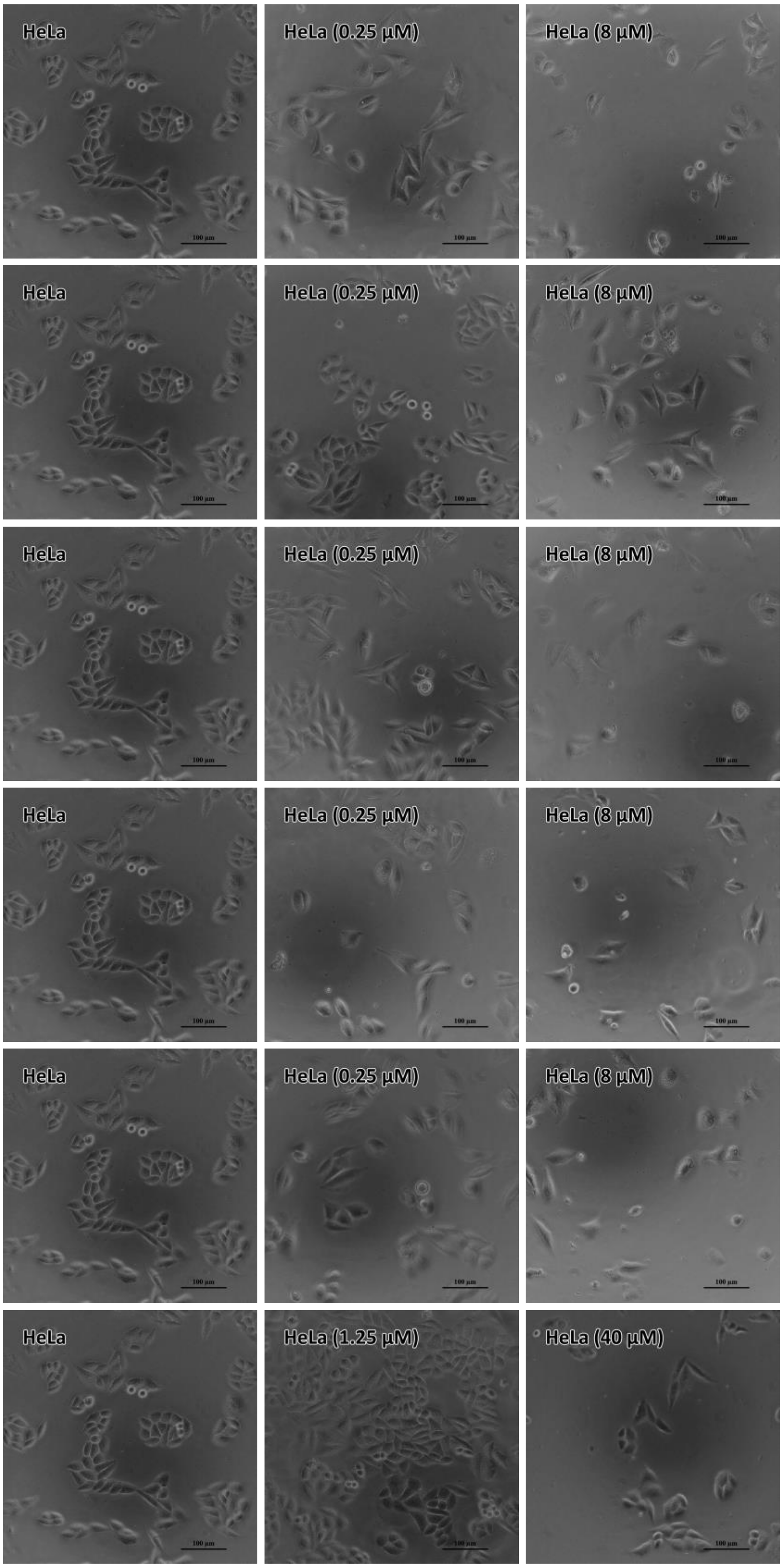

Figure S32 The microscopic images of HeLa cells treated with increased concentrations of the compounds **2d**–**2h** and cisplatin.

Compound | Control | Compound Low concentration → high concentration

1b

1c

2a

2b

2c

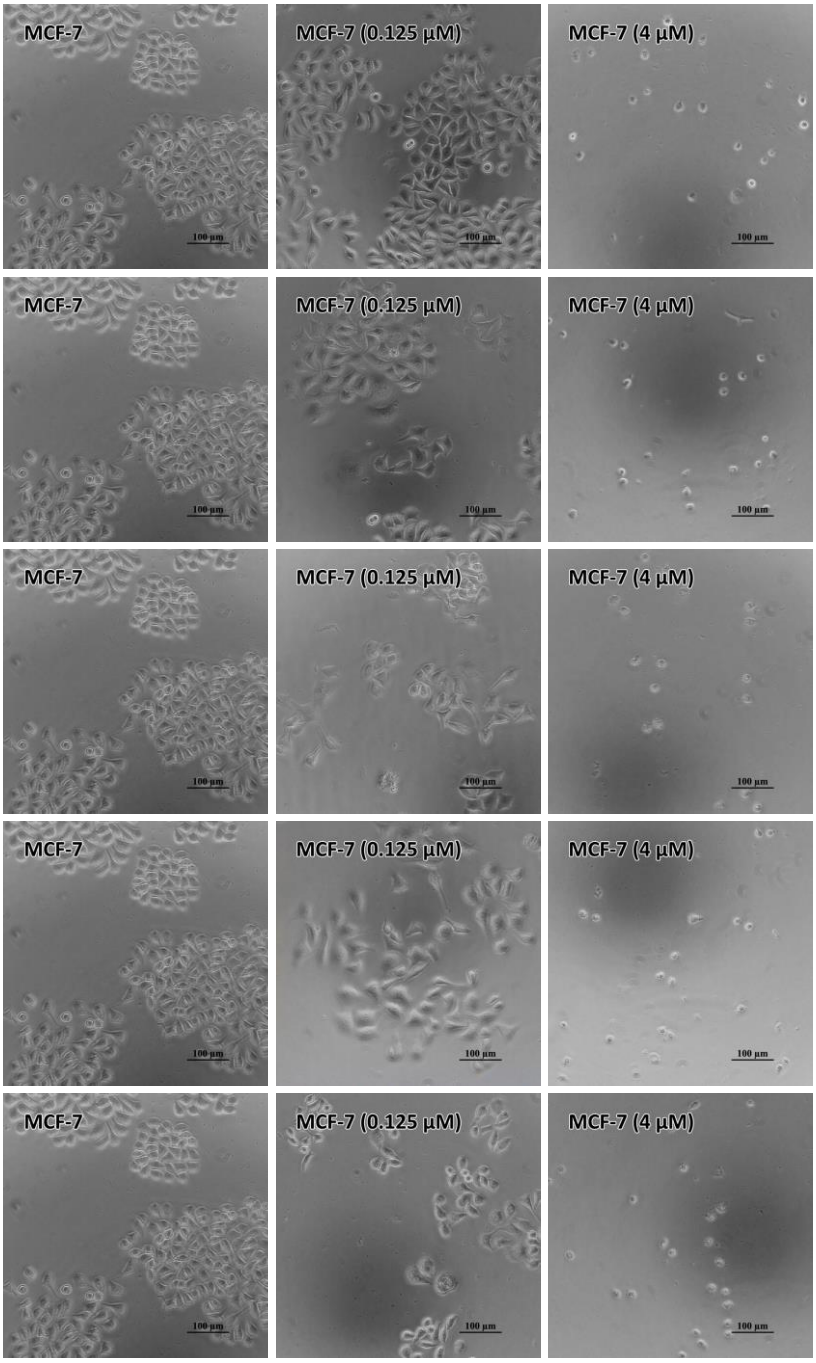

Figure S33 The microscopic images of MCF-7 cells treated with increased concentrations of the compounds **1b**, **1c** and **2a**–**2c**.

Compound | Control | Compound Low concentration → high concentration

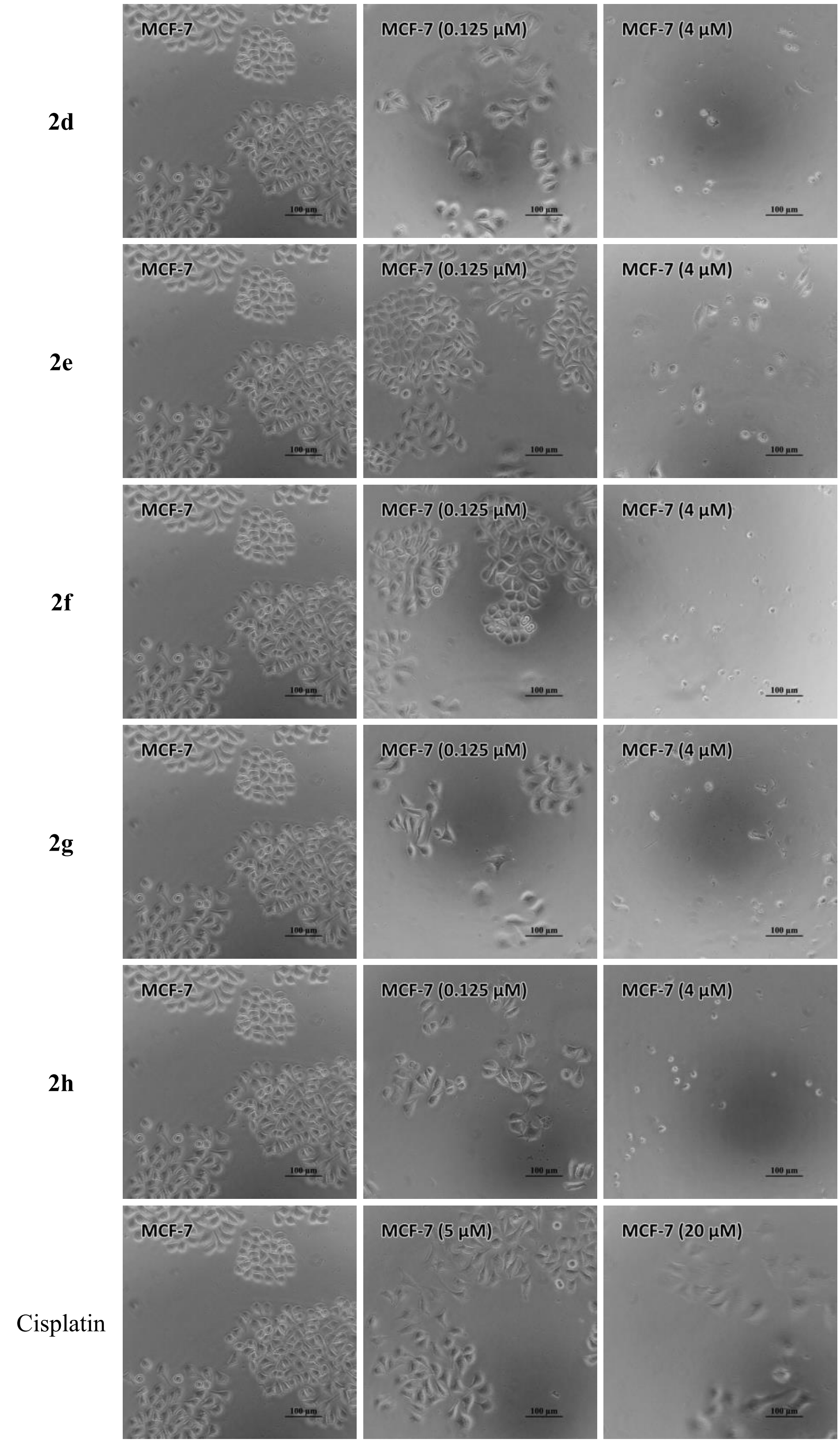

Figure S34 The microscopic images of MCF-7 cells treated with increased concentrations of the compounds **2d**–**2h** and cisplatin.

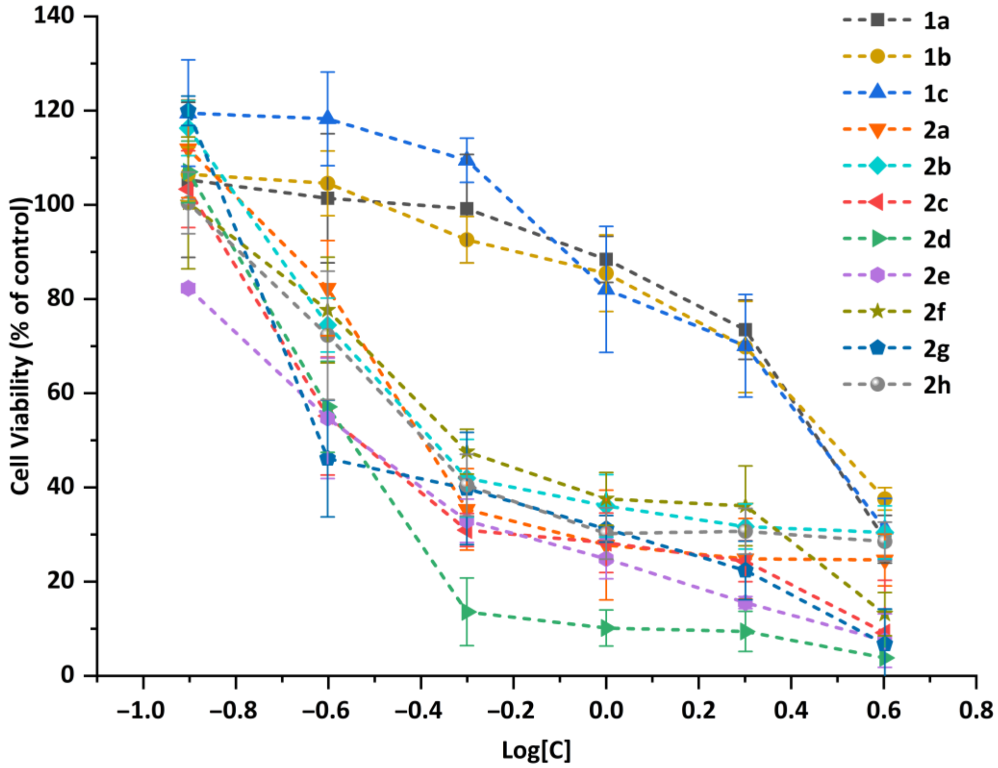

Figure S35 The plots of cell viability *vs.* the concentration of compounds **1a–1c** and **2a–2h** against A549 cells.

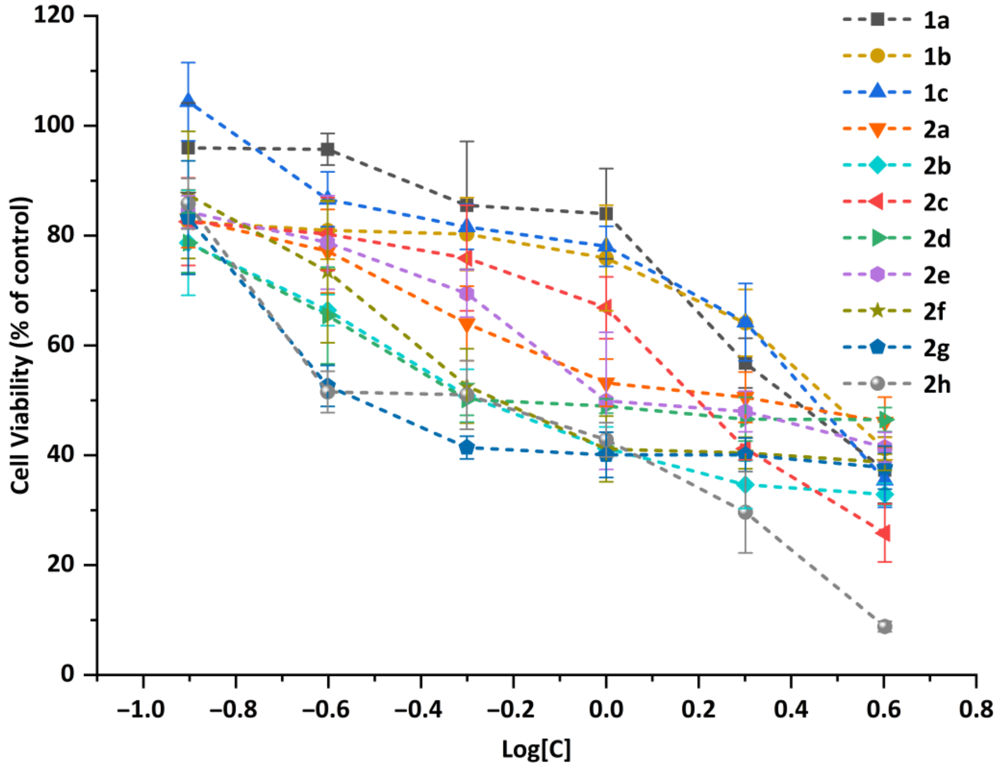

Figure S36 The plots of cell viability *vs.* the concentration of compounds **1a–1c** and **2a–2h** against Bel-7402 cells.

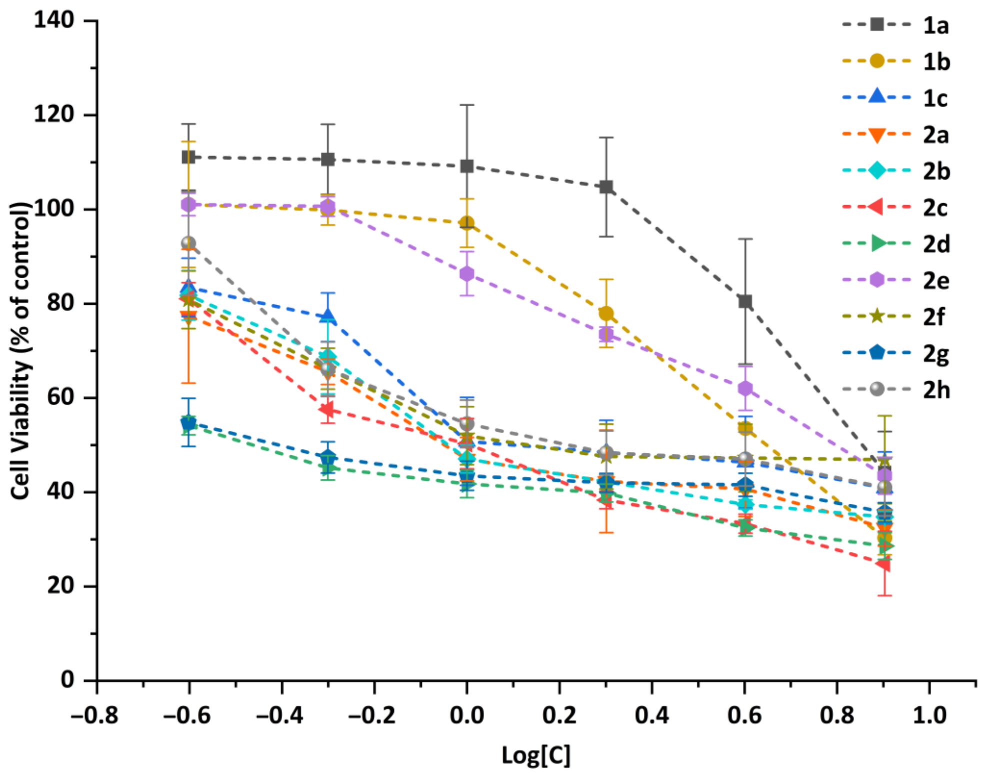

Figure S37 The plots of cell viability *vs.* the concentration of compounds **1a–1c** and **2a–2h** against HeLa cells.

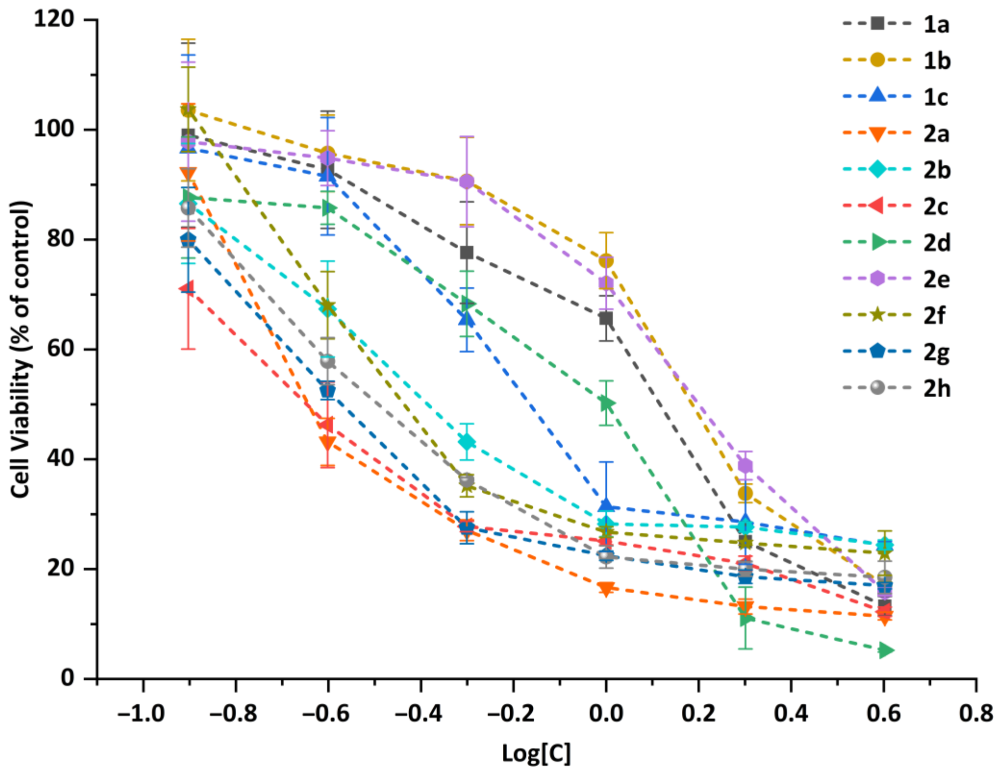

Figure S38 The plots of cell viability *vs.* the concentration of compounds **1a–1c** and **2a–2h** against MCF-7 cells.

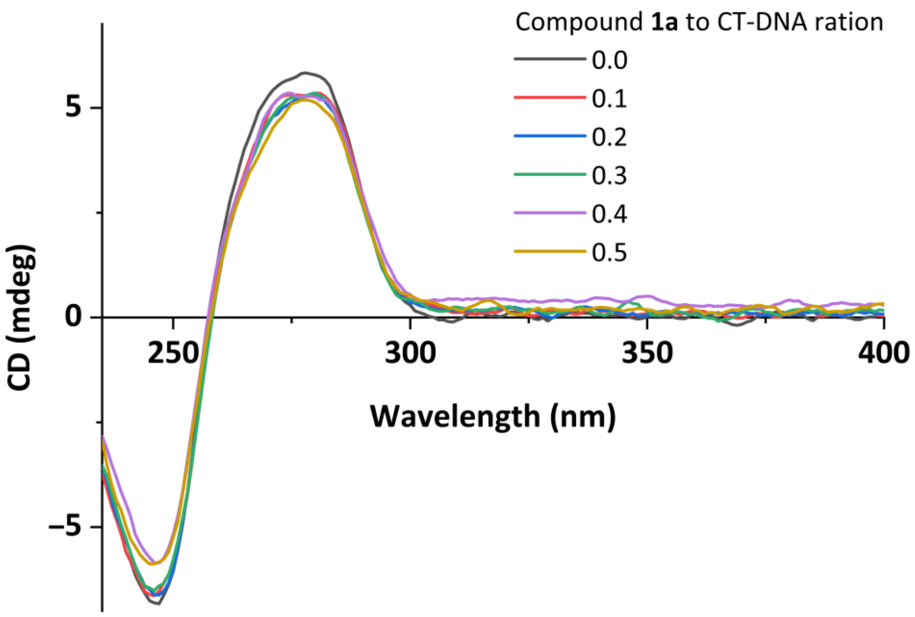

Figure S39 Circular dichroism spectra of CT-DNA (6.0×10^{-4} mol/L) in the presence or absence of compounds **1a** in Tris-HCl buffer (pH 7.2), at 20 °C.

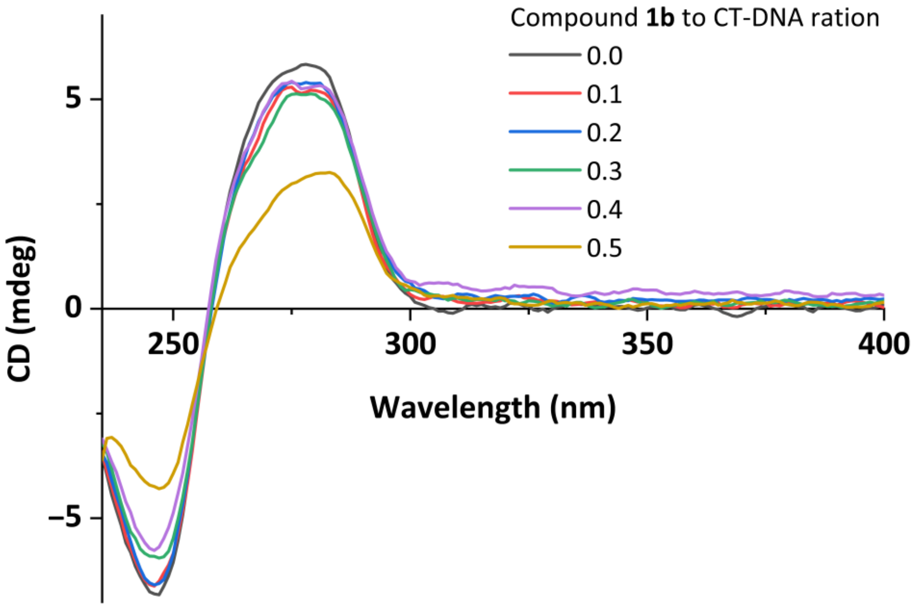

Figure S40 Circular dichroism spectra of CT-DNA (6.0×10^{-4} mol/L) in the presence or absence of compounds **1b** in Tris-HCl buffer (pH 7.2), at 20 °C.

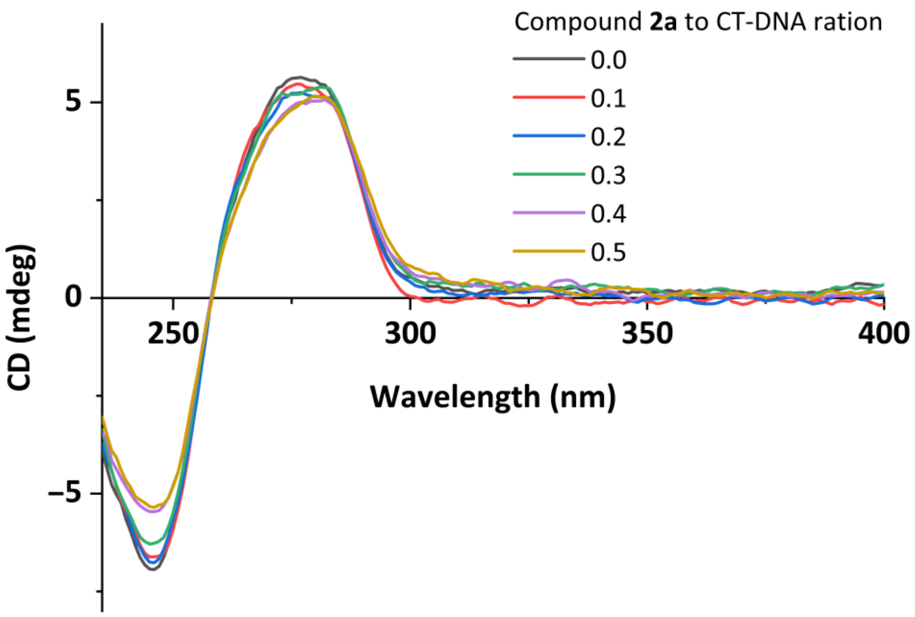

Figure S41 Circular dichroism spectra of CT-DNA (6.0×10^{-4} mol/L) in the presence or absence of compounds **2a** in Tris-HCl buffer (pH 7.2), at 20 °C.

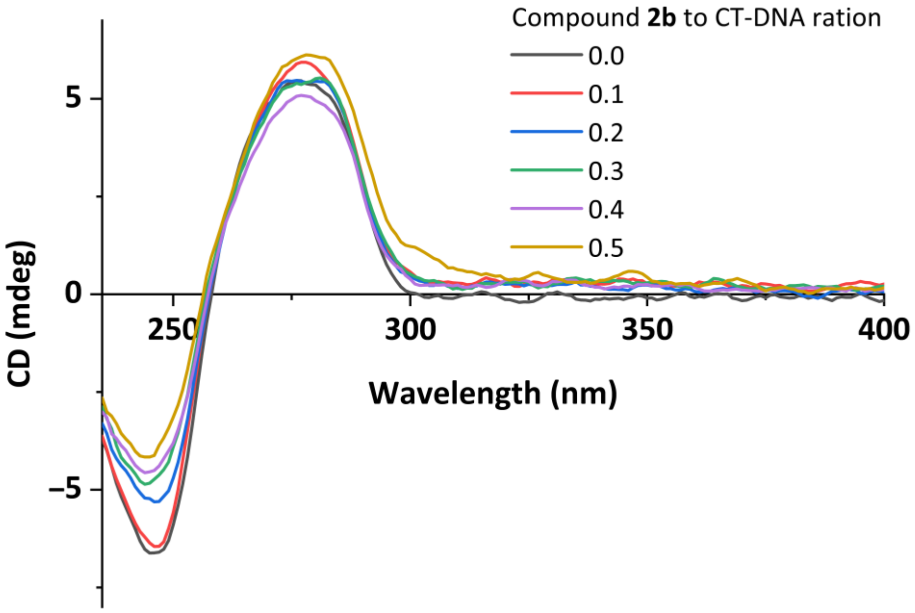

Figure S42 Circular dichroism spectra of CT-DNA (6.0×10^{-4} mol/L) in the presence or absence of compounds **2b** in Tris-HCl buffer (pH 7.2), at 20 °C.

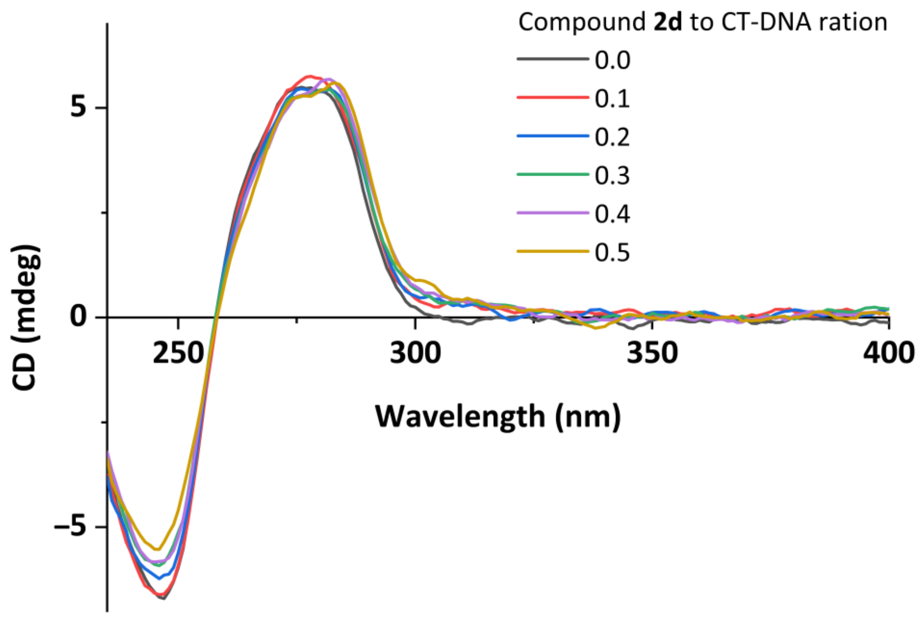

Figure S43 Circular dichroism spectra of CT-DNA (6.0×10^{-4} mol/L) in the presence or absence of compounds **2d** in Tris-HCl buffer (pH 7.2), at 20 °C.

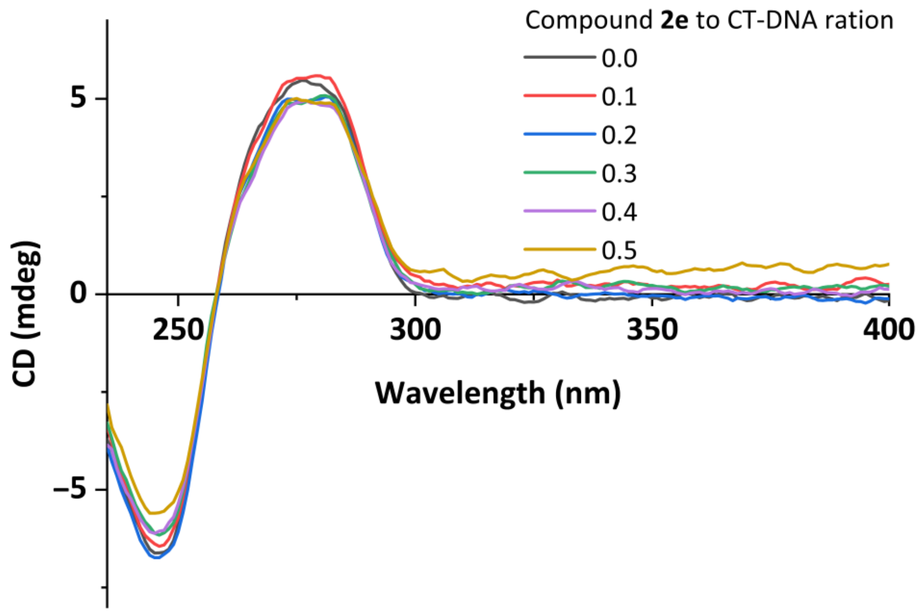

Figure S44 Circular dichroism spectra of CT-DNA (6.0×10^{-4} mol/L) in the presence or absence of compounds **2e** in Tris-HCl buffer (pH 7.2), at 20 °C.

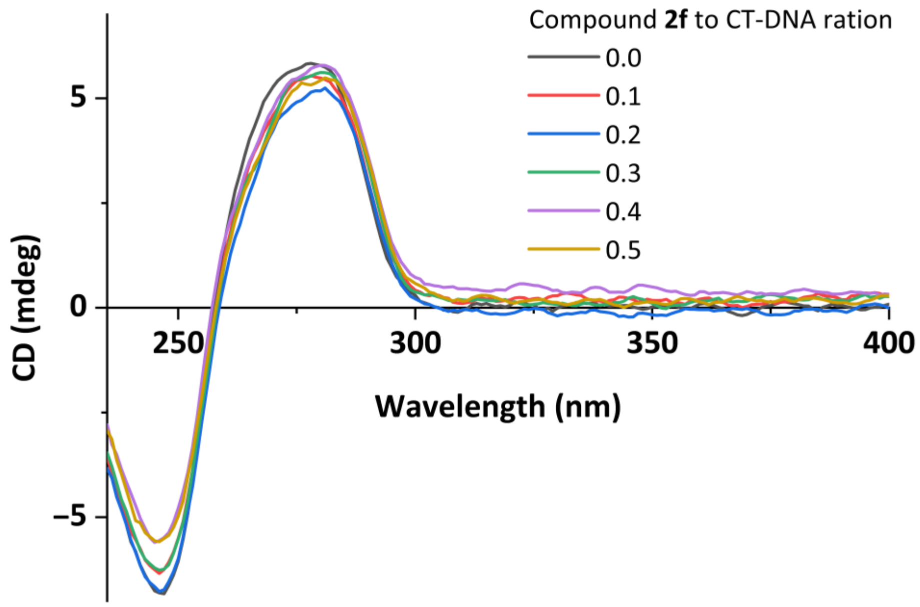

Figure S45 Circular dichroism spectra of CT-DNA (6.0×10^{-4} mol/L) in the presence or absence of compounds **2f** in Tris-HCl buffer (pH 7.2), at 20 °C.

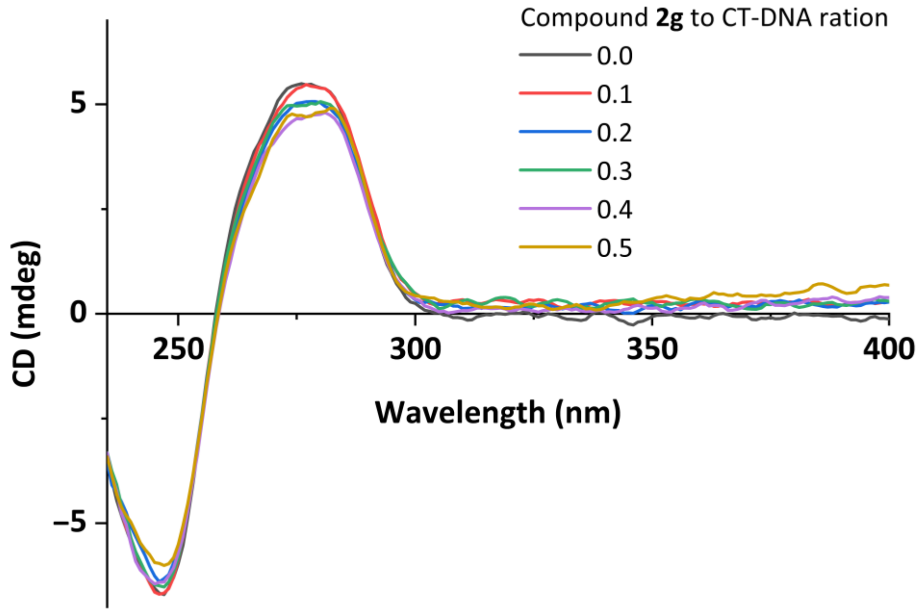

Figure S46 Circular dichroism spectra of CT-DNA (6.0×10^{-4} mol/L) in the presence or absence of compounds **2g** in Tris-HCl buffer (pH 7.2), at 20 °C.

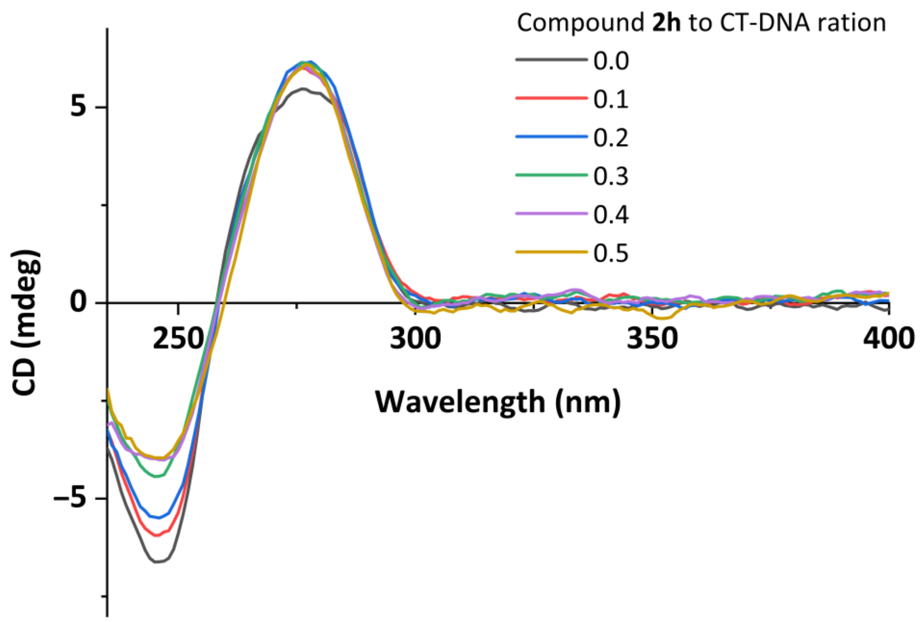

Figure S47 Circular dichroism spectra of CT-DNA (6.0×10^{-4} mol/L) in the presence or absence of compounds **2h** in Tris-HCl buffer (pH 7.2), at 20 °C.

Table S1 The CD spectral bands and change ratios of CT-DNA and CT-DNA with compounds **1a–1c** and **2a–2h**: wavelength λ (degree of ellipticity, Φ)

Conditions	λ/nm (Φ/°)		Change ratio (%)	
	(−) band	(+) band	(–) band	(+) band
600 µM CT-DNA + 60 µM compound **1a**	247(-6.53)	279(5.33)	-4	-3
600 µM CT-DNA + 120 µM compound **1a**	247(-6.62)	279(5.31)	-2	-3
600 µM CT-DNA + 180 µM compound **1a**	246(-6.51)	279(5.33)	-4	-3
600 µM CT-DNA + 240 µM compound **1a**	246(-5.85)	279(5.28)	-14	-3
600 µM CT-DNA + 300 µM compound **1a**	245(-5.88)	280(5.13)	-13	-6
600 µM CT-DNA + 60 µM compound **1b**	247(-6.51)	278(5.40)	-5	-7
600 µM CT-DNA + 120 µM compound **1b**	247(-6.56)	278(5.27)	-4	-9
600 µM CT-DNA + 180 µM compound **1b**	247(-5.95)	278(5.17)	-13	-11
600 µM CT-DNA + 240 µM compound **1b**	247(-5.69)	279(5.13)	-17	-12
600 µM CT-DNA + 300 µM compound **1b**	247(-4.30)	283(3.26)	-37	-44
600 µM CT-DNA + 60 µM compound **1c**	247(-6.45)	280(5.72)	-5	5
600 µM CT-DNA + 120 µM compound **1c**	247(-6.09)	282(5.94)	-10	9
600 µM CT-DNA + 180 µM compound **1c**	247(-5.80)	282(5.96)	-14	9
600 µM CT-DNA + 240 µM compound **1c**	248(-4.70)	283(6.87)	-31	26
600 µM CT-DNA + 300 µM compound **1c**	248(-4.63)	283(6.83)	-32	25
600 µM CT-DNA + 60 µM compound **2a**	246(-6.62)	277(5.46)	-5	-3
600 µM CT-DNA +120 µM compound **2a**	246(-6.77)	277(5.25)	-2	-7
600 µM CT-DNA + 180 µM compound **2a**	246(-6.27)	279(5.32)	-10	-6
600 µM CT-DNA + 240 µM compound **2a**	246(-5.46)	280(5.04)	-21	-11
600 µM CT-DNA + 300 µM compound **2a**	247(-5.35)	280(5.17)	-23	-8
600 µM CT-DNA + 60 µM compound **2b**	247(-6.45)	277(5.93)	-2	8
600 µM CT-DNA + 120 µM compound **2b**	246(-5.31)	278(5.42)	-20	-1
600 µM CT-DNA + 180 µM compound **2b**	245(-4.84)	278(5.42)	-27	-1
600 µM CT-DNA + 240 µM compound **2b**	244(-4.56)	278(5.07)	-31	-7
600 µM CT-DNA + 300 µM compound **2b**	244(-4.17)	278(6.12)	-37	12
600 µM CT-DNA + 60 µM compound **2c**	247(-6.77)	276(5.50)	0	0
600 µM CT-DNA + 120 µM compound **2c**	247(-6.11)	277(4.78)	-10	-13
600 µM CT-DNA + 180 µM compound **2c**	247(-5.19)	278(6.12)	-23	11
600 µM CT-DNA + 240 µM compound **2c**	247(-4.36)	282(5.76)	-36	5
600 µM CT-DNA + 300 µM compound **2c**	247(-2.87)	284(2.97)	-58	-46
600 µM CT-DNA + 60 µM compound **2d**	247(-6.60)	278(5.75)	-3	5
600 µM CT-DNA + 120 µM compound **2d**	246(-6.23)	282(5.45)	-8	0
600 µM CT-DNA + 180 µM compound **2d**	246(-5.92)	282(5.44)	-13	-1
600 µM CT-DNA + 240 µM compound **2d**	246(-5.82)	282(5.68)	-14	4
600 µM CT-DNA + 300 µM compound **2d**	246(-5.53)	283(5.60)	-18	2
600 µM CT-DNA + 60 µM compound **2e**	246(-6.45)	279(5.59)	-5	2
600 µM CT-DNA + 120 µM compound **2e**	246(-6.47)	281(5.05)	-4	-8
600 µM CT-DNA + 180 µM compound **2e**	246(-6.16)	281(5.08)	-9	-7
600 µM CT-DNA + 240 µM compound **2e**	245(-6.12)	282(4.82)	-10	-12
600 µM CT-DNA + 300 µM compound **2e**	245(-5.60)	282(4.89)	-17	-11
600 µM CT-DNA + 60 µM compound **2f**	247(-6.25)	278(5.84)	-8	7
600 µM CT-DNA + 120 µM compound **2f**	247(-6.73)	279(5.18)	-1	-5
600 µM CT-DNA + 180 µM compound **2f**	246(-6.27)	280(5.62)	-7	3
600 µM CT-DNA + 240 µM compound **2f**	246(-5.85)	280(5.26)	-14	-4
600 µM CT-DNA + 300 µM compound **2f**	245(-5.61)	280(5.79)	-17	6
600 µM CT-DNA + 60 µM compound **2g**	246(-6.69)	277(5.47)	-1	0
600 µM CT-DNA + 120 µM compound **2g**	246(-6.40)	279(5.07)	-5	-7
600 µM CT-DNA + 180 µM compound **2g**	246(-6.49)	280(5.07)	-4	-7
600 µM CT-DNA + 240 µM compound **2g**	246(-6.41)	281(4.80)	-5	-12
600 µM CT-DNA + 300 µM compound **2g**	246(-5.96)	282(4.91)	-12	-10

600 μM CT-DNA + 60 μM compound **2h**	246(-5.94)	277(5.99)	-10	10
600 μM CT-DNA + 120 μM compound **2h**	246(-5.49)	278(6.14)	-17	12
600 μM CT-DNA + 180 μM compound **2h**	246(-4.43)	278(6.13)	-33	12
600 μM CT-DNA + 240 μM compound **2h**	246(-4.00)	277(6.03)	-40	10
600 μM CT-DNA + 300 μM compound **2h**	246(-3.96)	277(6.10)	-40	12

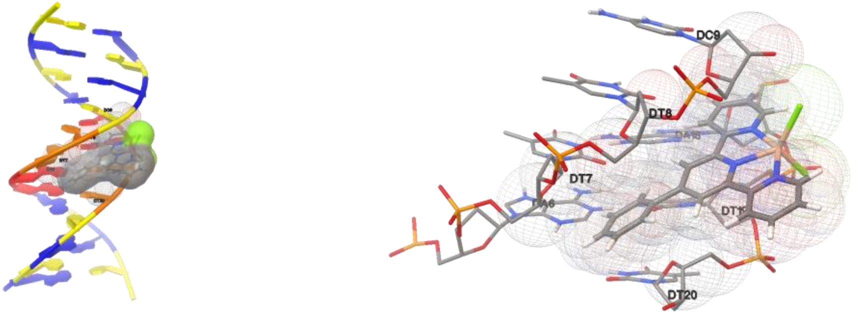

Figure S48 The most favorable orientation of compound **1a** with the minor groove of the B-DNA (PDB ID: 1BNA).

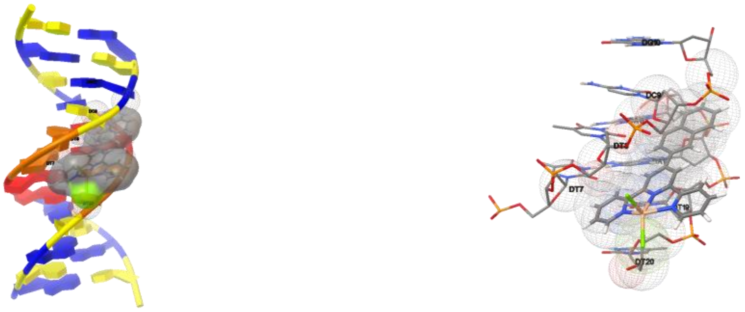

Figure S49 The most favorable orientation of compound **1b** with the minor groove of the B-DNA (PDB ID: 1BNA).

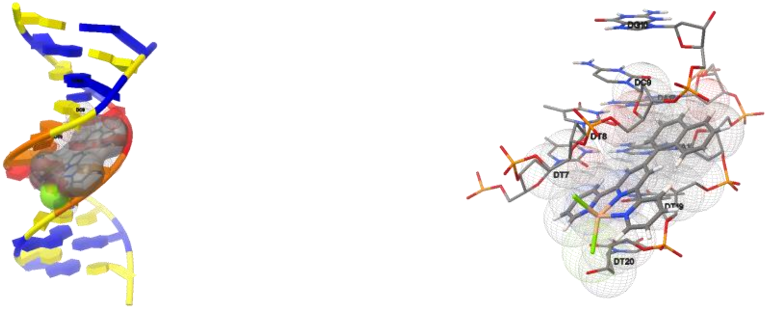

Figure S50 The most favorable orientation of compound **1c** with the minor groove of the B-DNA (PDB ID: 1BNA).

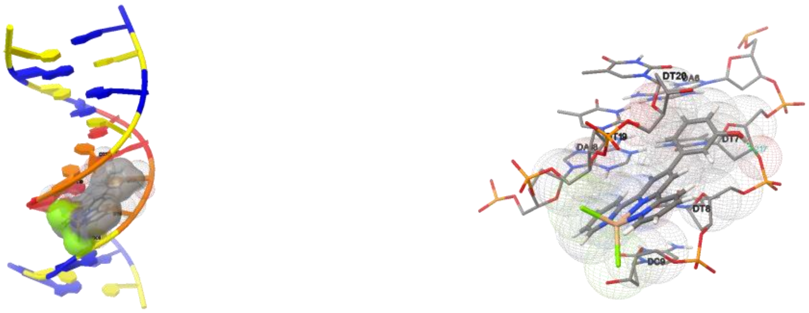

Figure S51 The most favorable orientation of compound **2b** with the minor groove of the B-DNA (PDB ID: 1BNA). The formed hydrogen bond and distance have been marked.

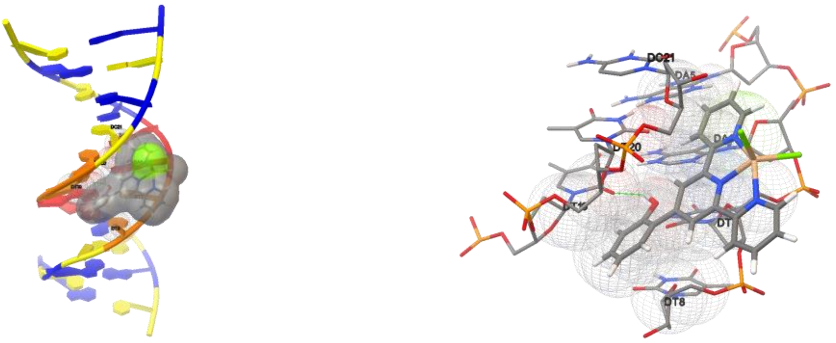

Figure S52 The most favorable orientation of compound **2c** with the minor groove of the B-DNA (PDB ID: 1BNA). The formed hydrogen bond and distance have been marked.

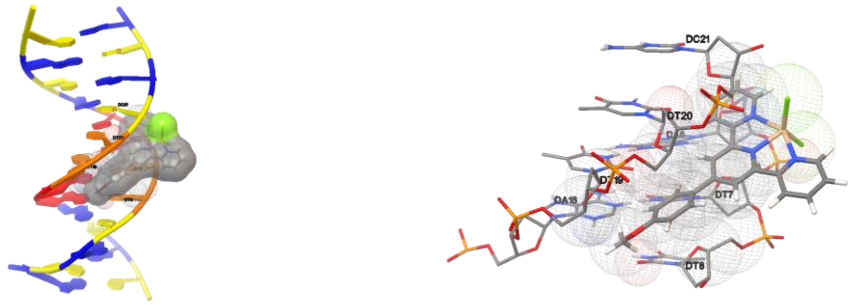

Figure S53 The most favorable orientation of compound **2d** with the minor groove of the B-DNA (PDB ID: 1BNA).

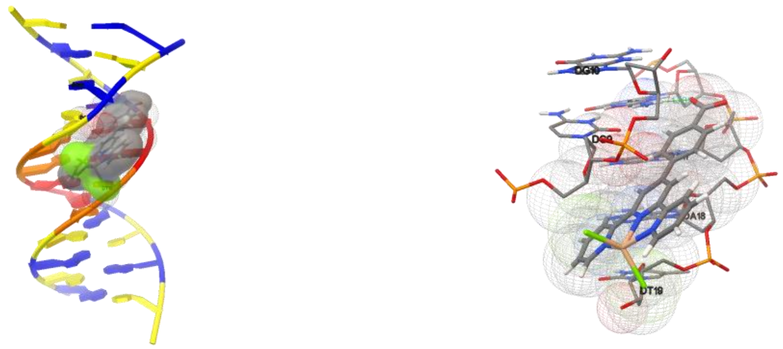

Figure S54 The most favorable orientation of compound **2e** with the minor groove of the B-DNA (PDB ID: 1BNA). The formed hydrogen bond and distance have been marked.

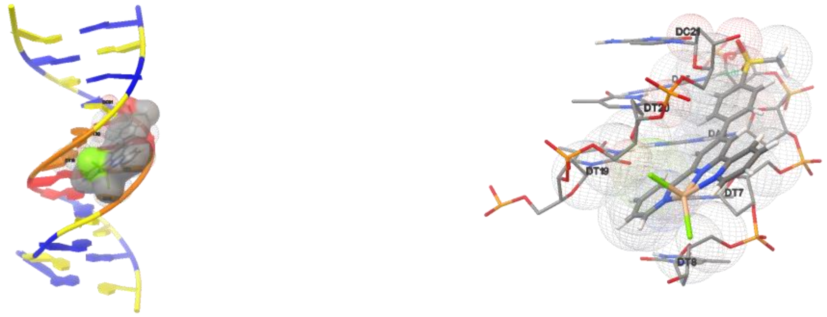

Figure S55 The most favorable orientation of compound **2f** with the minor groove of the B-DNA (PDB ID: 1BNA). The formed hydrogen bond and distance have been marked.

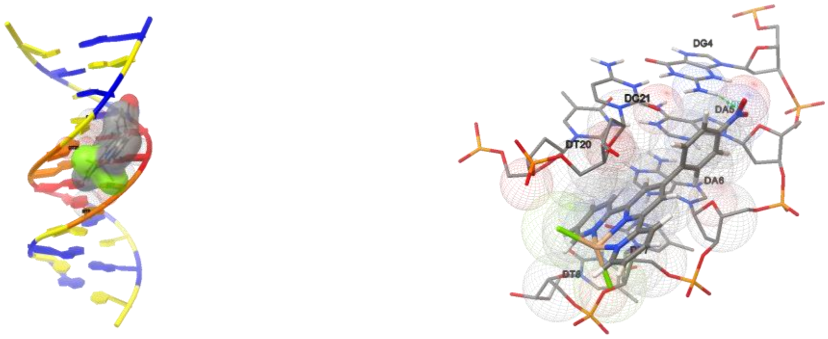

Figure S56 The most favorable orientation of compound **2g** with the minor groove of the B-DNA (PDB ID: 1BNA). The formed hydrogen bond and distance have been marked.

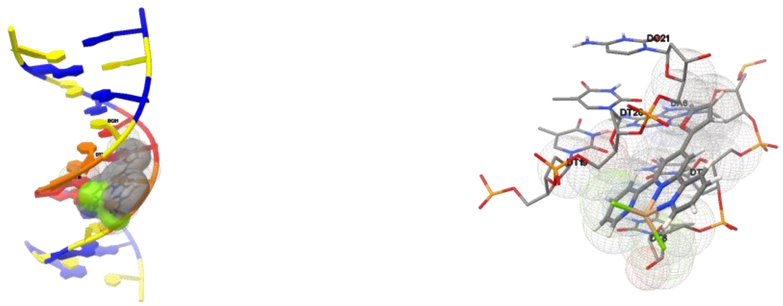

Figure S57 The most favorable orientation of compound **2h** with the minor groove of the B-DNA (PDB ID: 1BNA).

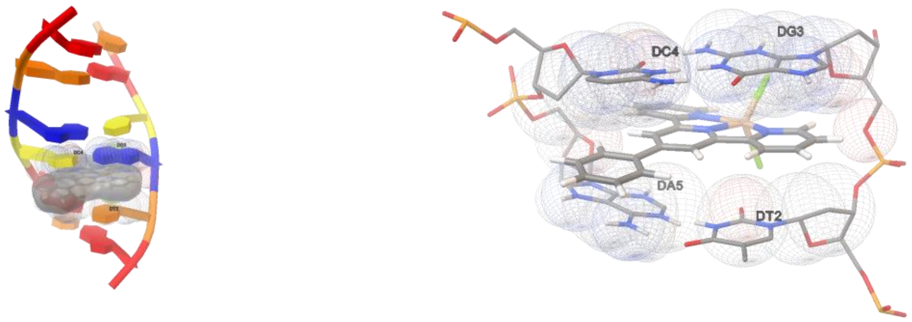

Figure S58 The most favorable orientation of compound **1a** intercalating with the DNA (4JD8).

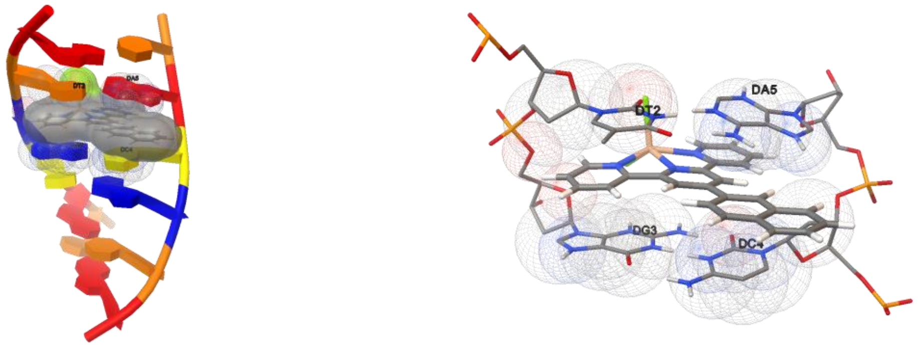

Figure S59 The most favorable orientation of compound **1b** intercalating with the DNA (4JD8).

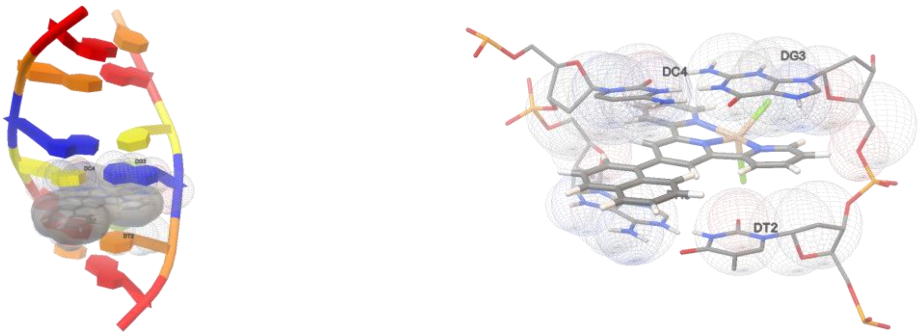

Figure S60 The most favorable orientation of compound **1c** intercalating with the DNA (4JD8).

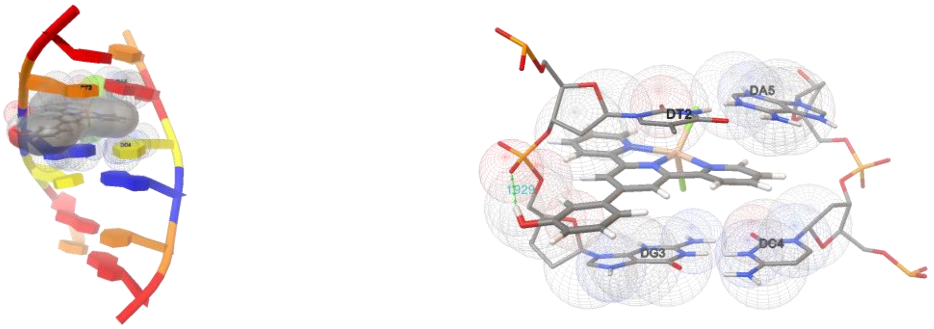

Figure S61 The most favorable orientation of compound **2b** intercalating with the DNA (4JD8). The formed hydrogen bond and distance have been marked.

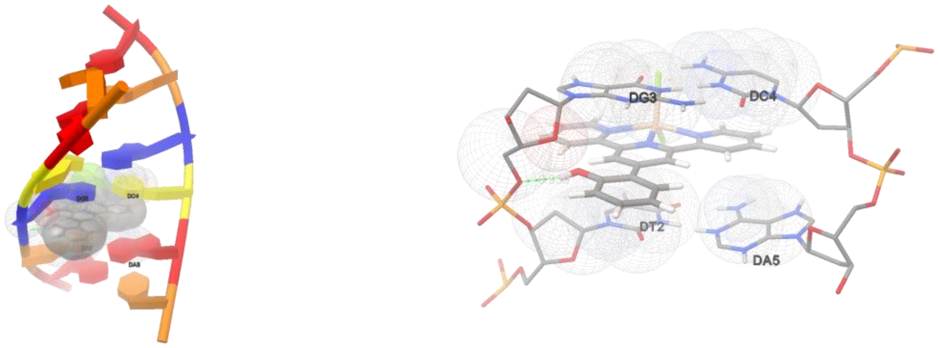

Figure S62 The most favorable orientation of compound **2c** intercalating with the DNA (4JD8). The formed hydrogen bond and distance have been marked.

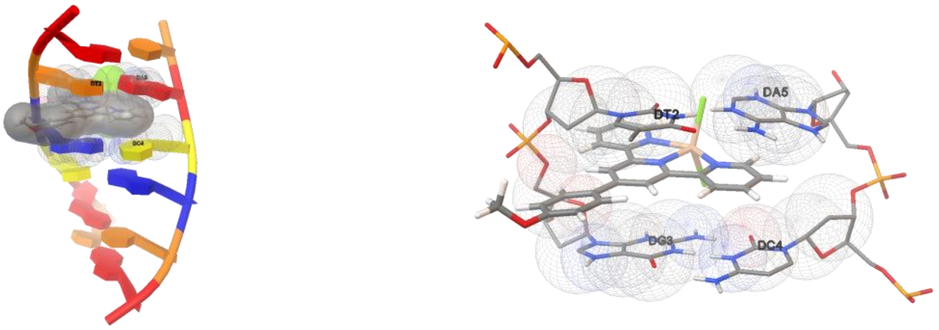

Figure S63 The most favorable orientation of compound **2d** intercalating with the DNA (4JD8).

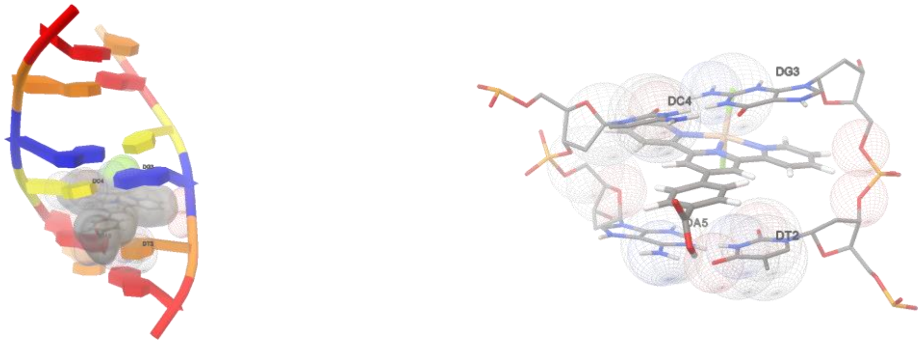

Figure S64 The most favorable orientation of compound **2e** intercalating with the DNA (4JD8).

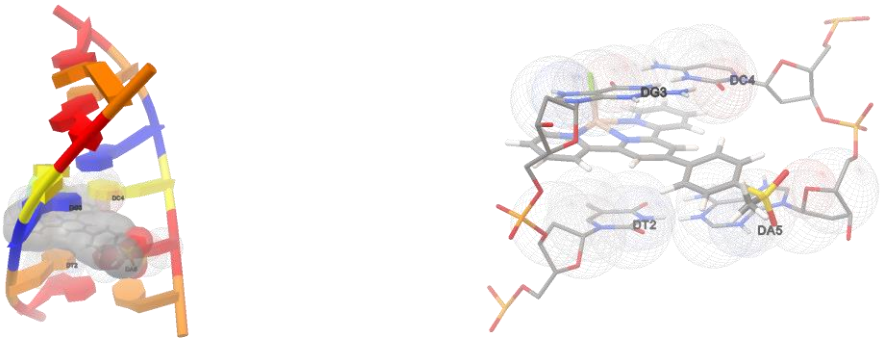

Figure S65 The most favorable orientation of compound **2f** intercalating with the DNA (4JD8).

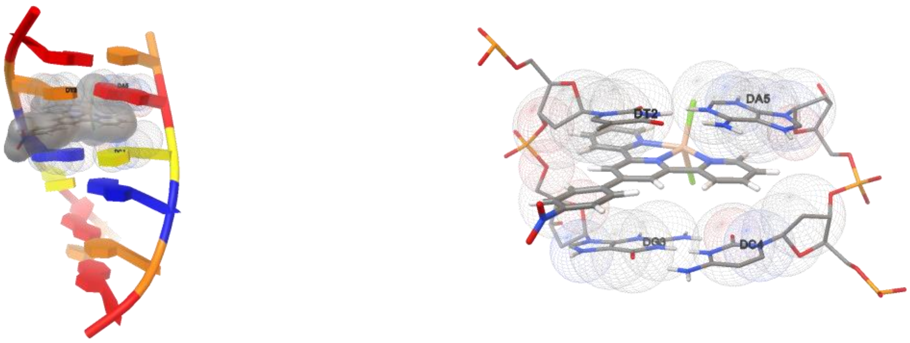

Figure S66 The most favorable orientation of compound **2g** intercalating with the DNA (4JD8).

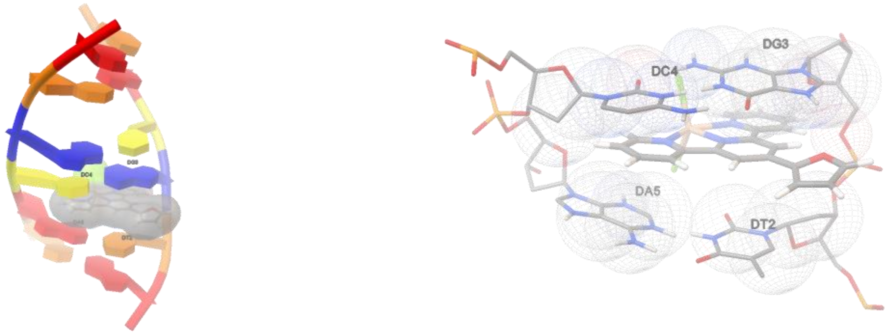

Figure S67 The most favorable orientation of compound **2h** intercalating with the DNA (4JD8).

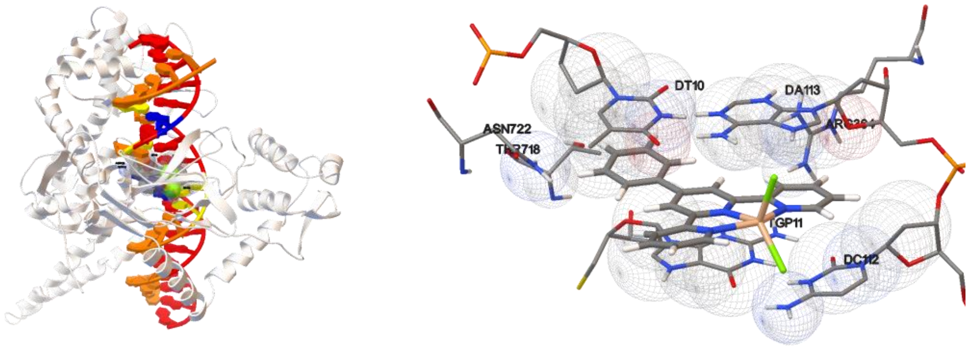

Figure S68 Molecular docking models of compound **1a** in the active site of DNA–Topo I complex (PDB ID: 1SC7).

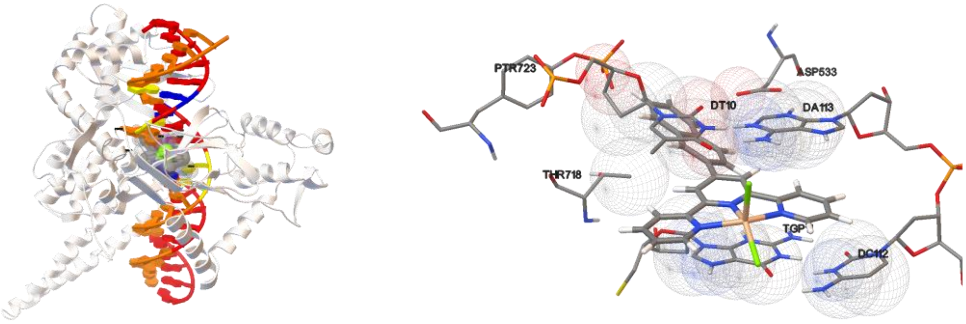

Figure S69 Molecular docking models of compound **1b** in the active site of DNA–Topo I complex (PDB ID: 1SC7).

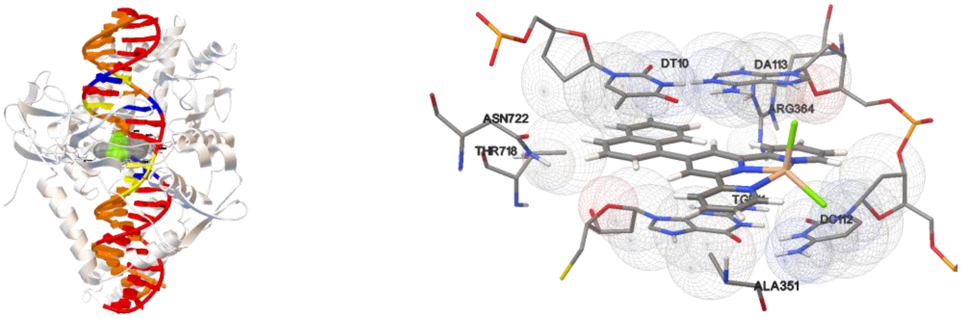

Figure S70 Molecular docking models of compound **1c** in the active site of DNA–Topo I complex (PDB ID: 1SC7).

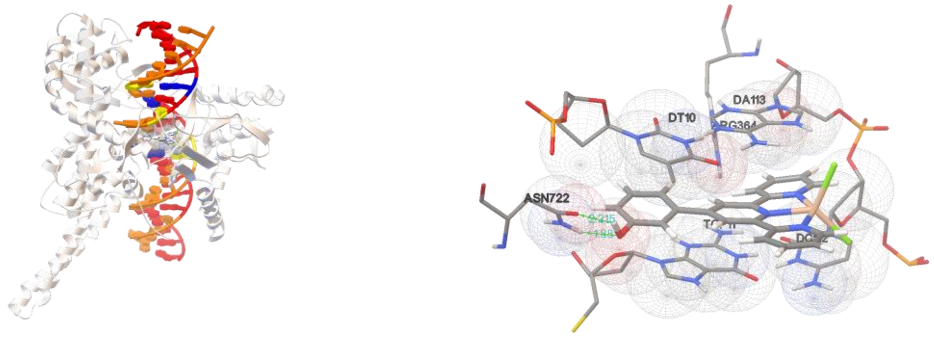

Figure S71 Molecular docking models of compound **2b** in the active site of DNA–Topo I complex (PDB ID: 1SC7). The formed hydrogen bond and distance have been marked.

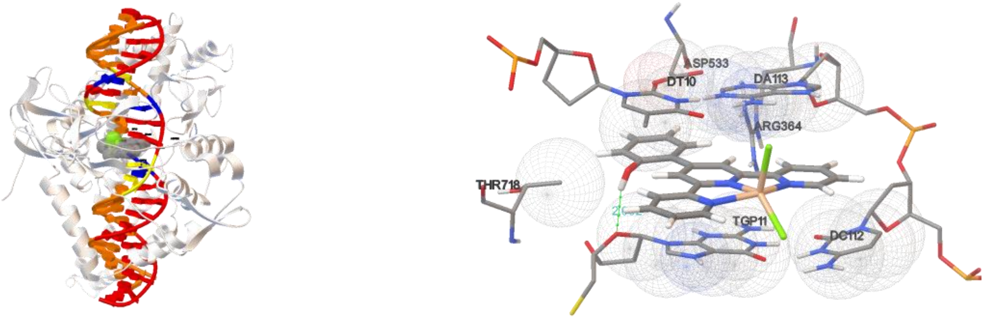

Figure S72 Molecular docking models of compound **2c** in the active site of DNA–Topo I complex (PDB ID: 1SC7). The formed hydrogen bond and distance have been marked.

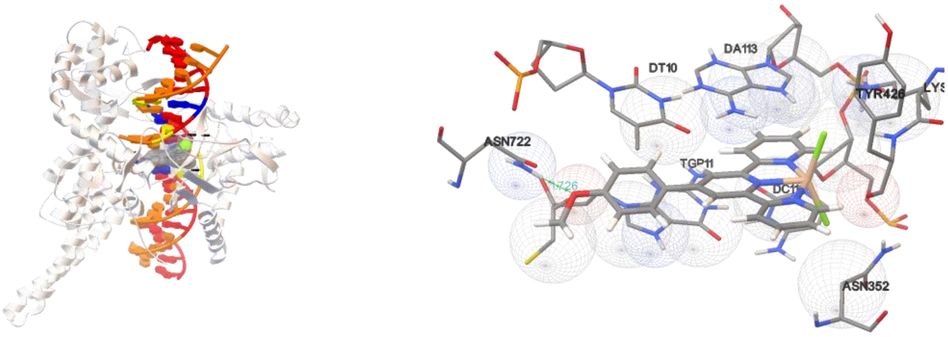

Figure S73 Molecular docking models of compound **2d** in the active site of DNA–Topo I complex (PDB ID: 1SC7). The formed hydrogen bond and distance have been marked.

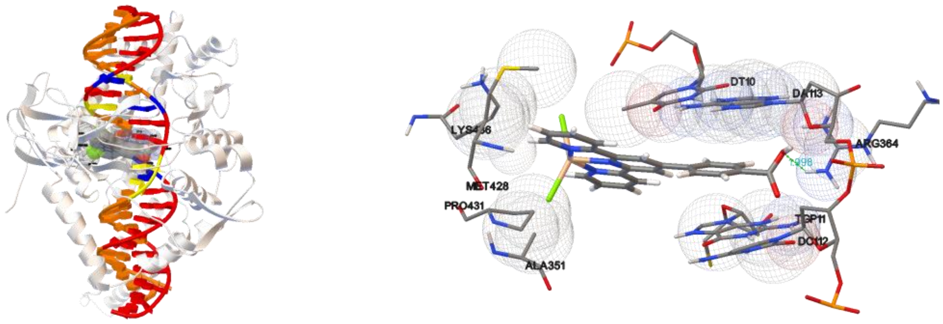

Figure S74 Molecular docking models of compound **2e** in the active site of DNA–Topo I complex (PDB ID: 1SC7). The formed hydrogen bond and distance have been marked.

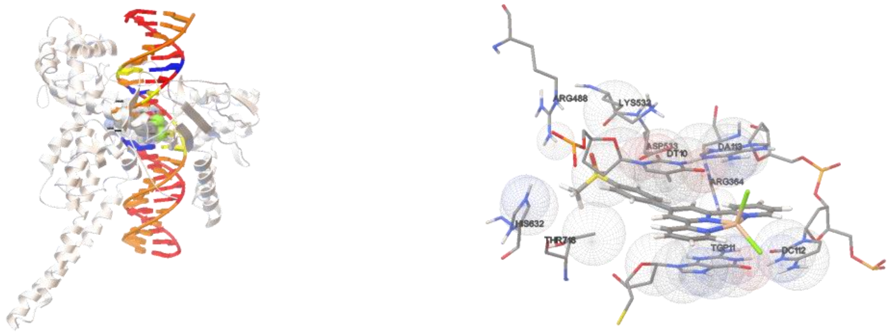

Figure S75 Molecular docking models of compound **2f** in the active site of DNA–Topo I complex (PDB ID: 1SC7).

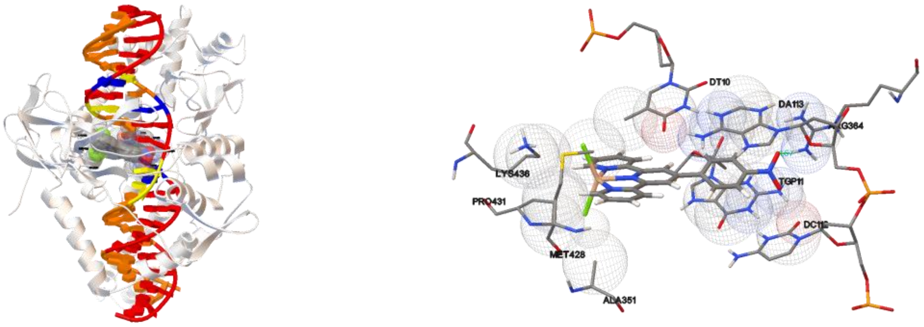

Figure S76 Molecular docking models of compound **2g** in the active site of DNA–Topo I complex (PDB ID: 1SC7). The formed hydrogen bond and distance have been marked.

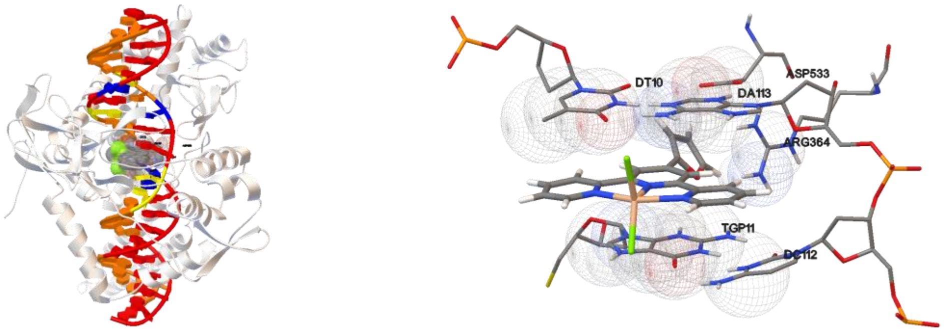

Figure S77 Molecular docking models of compound **2h** in the active site of DNA–Topo I complex (PDB ID: 1SC7).
